# Micro/Nanorobots for Combating Brain Disorders: Challenges, Advances, and Perspectives

**DOI:** 10.1002/advs.202516592

**Published:** 2025-10-17

**Authors:** Qi Zhang, Dong Sun

**Affiliations:** ^1^ Department of Biomedical Engineering City University of Hong Kong Hong Kong SAR 999077 China; ^2^ Hong Kong Center for Cerebro‐Cardiovascular Health Engineering (COCHE) Hong Kong SAR 999077 China

**Keywords:** blood–brain barrier, brain disorders, drug delivery, micro/nanorobots

## Abstract

Brain disorders pose a significant global health burden, underscoring the urgent need for innovative therapeutic strategies. Conventional treatments are often hindered by poor drug penetration, systemic side effects, and the complex biological barriers of the central nervous system. In recent years, micro/nanorobots (MNRs) have emerged as promising platforms to overcome these limitations. Operating at the micro‐ to nanoscale, MNRs can accomplish targeted biomedical tasks through self‐propulsion (chemical or biohybrid) or external actuation (acoustic, optical, electric, or magnetic), thus enabling unprecedented precision in therapeutic delivery. This review systematically outlines the challenges in treating brain disorders, including major disease categories and barriers affecting therapeutic efficacy, and highlights emerging strategies addressing these obstacles. The principles of MNR propulsion and spotlight recent advances in applying MNRs is further summarized for brain disorder therapies, with special emphasis on the crucial roles of image guidance and real‐time tracking in facilitating clinical translation. Finally, it discusses challenges and provides perspectives on future directions. Overall, the rapid development of MNRs holds transformative potential to reshape therapeutic paradigms and accelerate clinical translation for advanced brain disorder treatments.

## Graphical abstract

Micro/nanorobots (MNRs) offer a transformative approach for precision therapy in brain disorders. By overcoming biological barriers and enabling controlled propulsion, targeted delivery, and real‐time imaging/tracking, MNRs pave the way toward effective, minimally invasive treatments. This review outlines the interplay between brain disease challenges, therapeutic barriers, MNR propulsion strategies, and imaging‐guided applications, highlighting their potential to revolutionize the clinical management of neurological disorders.

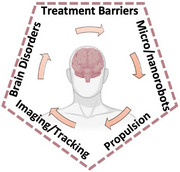

## 1 Introduction

Rapid advancements in medical technologies have significantly enhanced diagnostic and therapeutic outcomes for numerous diseases; however, effectively treating brain disorders remains a substantial clinical challenge. Brain disorders, including cerebrovascular diseases (CVDs), neurodegenerative conditions, primary and metastatic brain tumors, infectious and inflammatory brain diseases, traumatic brain injuries, and psychiatric disorders, have emerged as leading global causes of disability and mortality. With populations aging rapidly across the globe, the prevalence of neurological conditions, such as Alzheimer's disease (AD), Parkinson's disease (PD), and stroke, continues to increase, placing substantial strain on personal health and broader socioeconomic systems. Malignant brain tumors, such as meningiomas and gliomas, further intensify this challenge due to their aggressive nature and poor clinical prognoses. Although significant progress has been achieved in diagnostic techniques and therapeutic approaches, effective treatments for brain disorders remain scarce. This challenge is largely due to the complex structure of the brain, the presence of biological barriers, particularly the blood–brain barrier (BBB) and the blood–brain tumor barrier (BBTB); and the limited efficiency of current drug delivery methods.

In recent years, advances in micro‐ and nanotechnology have presented promising opportunities to overcome persistent barriers in targeted therapies for brain disorders. Micro/nanorobots (MNRs), characterized by their micro‐ and nanoscale dimensions, programmable features, and precise controllability, have emerged as powerful biomedical platforms capable of crossing biological barriers and achieving highly efficient targeted drug delivery. MNRs can be driven via autonomous propulsion mechanisms involving chemical reactions or biohybrid approaches, as well as externally actuated using magnetic fields, optical fields, ultrasound, or electric fields. Micro‐ and nanorobotic systems have shown considerable promise in executing complex tasks at the microscale, particularly in targeted drug delivery across various physiological environments, including the gastrointestinal tract, blood vessels, bladder, and ocular tissues. The integration of MNRs with advanced imaging technologies enables closed‐loop feedback control and precise therapeutic interventions, opening novel avenues toward personalized and intelligent treatment strategies for brain disorders.

**Figure 1:**
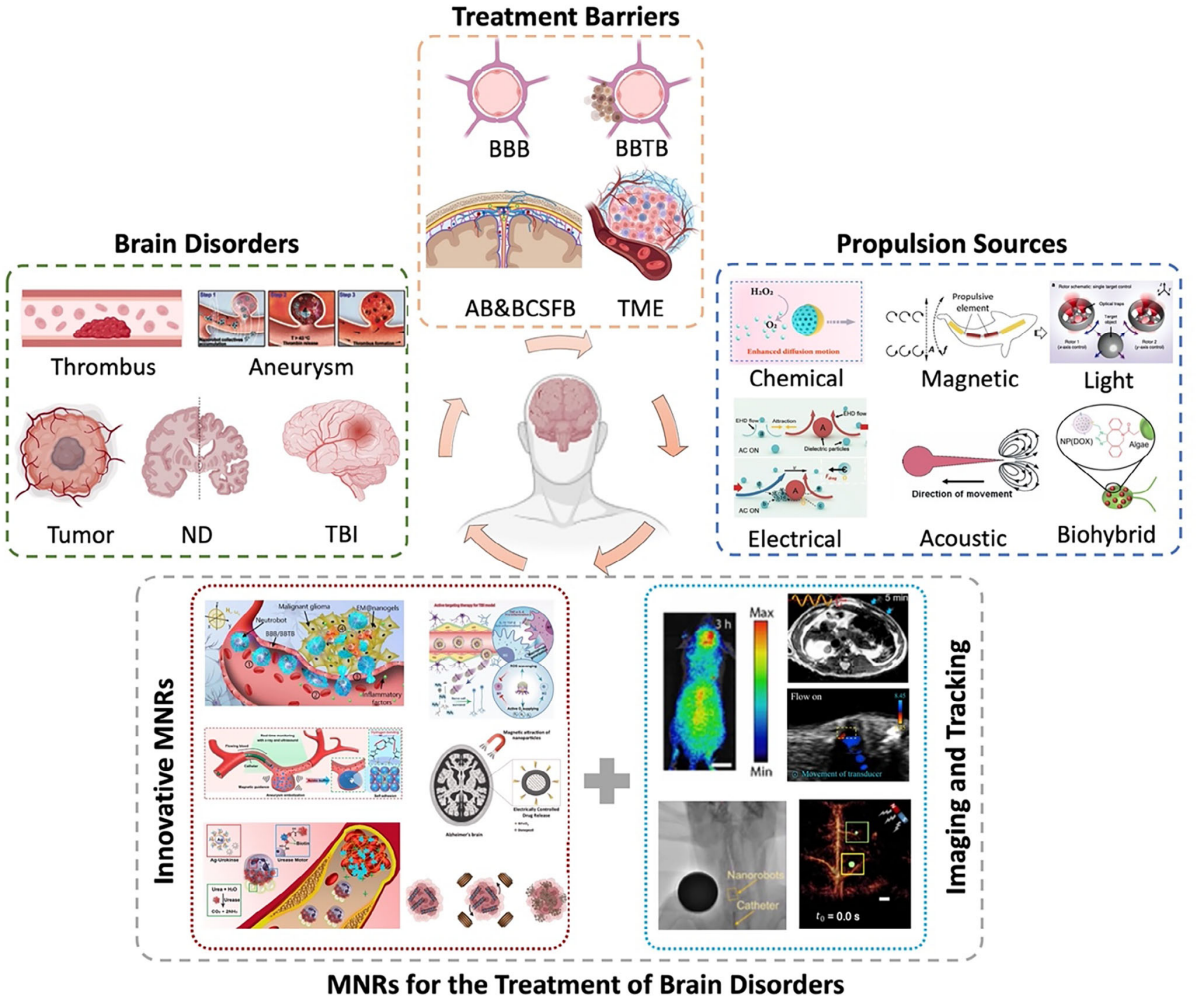
Micro/nanorobots for combating brain disorders, including brain disorders, treatment barriers, propulsion sources of micro/nanorobots, and applications of micro/nanorobots in the treatment of brain disorders. Some of the illustrations were created at BioRender.com.

This review systematically outlines the current challenges in brain disorder therapies and comprehensively summarizes the recent advances and future perspectives of medical micro‐ and nanorobotic technologies (**Figure** **1**). First, this review outlines the wide range of brain disorders and the biological barriers associated with them, followed by an in‐depth analysis of current strategies for targeted brain delivery, with a focus on approaches designed to bypass or cross the BBB. Subsequently, MNRs are systematically classified in accordance with their structural composition (synthetic and biohybrid) and propulsion mechanisms (chemically powered, externally field‐driven, and biohybrid‐driven). Next, the emerging therapeutic applications of MNRs specific to brain disorders are thoroughly examined, highlighting their integration with imaging‐guided navigation and real‐time tracking technologies that are pivotal for precision medicine. Finally, major challenges confronting the future development of MNRs are outlined, and perspectives on promising directions to accelerate the advancement of this rapidly evolving research frontier are provided, ultimately facilitating the clinical translation of MNR‐based therapeutic approaches for brain disorders.

## 2 Challenges of Brain Disorders Treatment

The following sections provide a comprehensive discussion of brain disorders and the challenges associated with delivering therapeutic agents to the central nervous system (CNS). First, major categories are classified—CVDs, neurodegenerative disorders, brain tumors, infectious and inflammatory conditions, traumatic brain injuries, and psychiatric illnesses—and their substantial public health influence is highlighted using key epidemiological indicators such as prevalence, incidence, and mortality rates. Subsequently, the key barriers impeding effective brain drug delivery, covering biological and pharmacological barriers, clinical and methodological barriers, and socioeconomic and ethical barriers are explored, and their implications for therapeutic strategies are discussed. An in‐depth understanding of these challenges is crucial for advancing innovative approaches, notably medical MNRs, aimed at enhancing therapeutic efficacy and patient outcomes.

### 2.1 Classification and Impact of Brain Disorders

The complexity and heterogeneity of brain disorders significantly limit their therapeutic efficacy. Major categories of these disorders include CVDs, neurodegenerative diseases (NDs), brain tumors, infectious and inflammatory brain diseases, traumatic brain injuries (TBIs), and psychiatric disorders.^[^ ^1^ ^]^ The various types of brain disorders are summarized in **Table** **1**.

**Table 1:** Types of brain disorders.

Classification	Examples	Pathological Features	Typical Clinical Manifestations	Impact on Patients and Society
Cerebrovascular diseases	Stroke, transient ischemic attack, cerebral aneurysm, and vascular dementia	Impaired blood supply, infarction, hemorrhage, and vascular degeneration	Acute neurological deficits, paralysis, aphasia, and cognitive impairment	Long‐term disability, high mortality, reduced independence, and economic burden
Neurodegenerative diseases	Alzheimer's disease, Parkinson's disease, amyotrophic lateral sclerosis, and Huntington's disease	Progressive neuronal loss, protein aggregation, and neuroinflammation	Dementia, motor dysfunction, cognitive decline, and speech impairment	Progressive disability, caregiver burden, and significant healthcare costs
Brain tumors	Glioblastoma, astrocytoma, meningioma, and pituitary adenoma	Abnormal cell proliferation, brain tissue compression, and infiltration	Headache, seizures, focal neurological deficits, and cognitive impairment	High morbidity and mortality, impaired quality of life, and complex medical management
Infectious and inflammatory brain diseases	Meningitis, encephalitis, multiple sclerosis, and neurocysticercosis	Inflammation, infection‐induced neuronal damage, and autoimmune processes	Fever, headache, seizures, cognitive deficits, and motor weakness	Acute and chronic neurological impairment, disability, and potential long‐term cognitive dysfunction
Traumatic brain injuries	Concussion, contusion, diffuse axonal injury, and hematoma	Mechanical injury, neuronal damage, diffuse axonal injury, and hemorrhage	Loss of consciousness, memory impairment, headache, and behavioral changes	Chronic cognitive and emotional impairment, decreased productivity, and increased risk of neurodegeneration
Psychiatric disorders	Depression, schizophrenia, anxiety disorders, and bipolar disorder	Neurochemical imbalances, functional brain abnormalities, and impaired neural connectivity	Mood disturbances, hallucinations, anxiety, and impaired social cognition	Reduced life quality, social isolation, high economic cost, and increased suicide risk

CVDs represent a group of disorders resulting from pathological changes in the cerebral blood vessels, typically leading to impaired blood flow in the brain and causing serious neurological damage or even death.^[^ ^2^ ^]^ Major types include cerebral infarction (ischemic stroke), cerebral hemorrhage (hemorrhagic stroke), transient ischemic attack, and cerebral aneurysm.^[^ ^3^ ^]^ The prevalence of CVDs has shown a rising trend in recent years due to accelerated population aging. Globally, more than 15 million individuals have experienced a stroke, and stroke incidence significantly increases with advancing age.^[^ ^4^ ^]^ At present, acute ischemic stroke treatment commonly involves intravenous administration of tissue‐type plasminogen activator (tPA) to dissolve thrombi.^[^ ^5^ ^]^ However, this therapeutic approach suffers from significant limitations, including the requirement for high dosage and strict therapeutic timing (typically optimal within 48 h), and increased risk of complications.^[^ ^6^ ^]^

NDs are characterized by the progressive deterioration of neurons, leading to a gradual decline in their structure and function.^[^ ^7^ ^]^ Common examples include amyotrophic lateral sclerosis, PD, AD, and Huntington's disease.^[^ ^8^, ^9^, ^10^, ^11^, ^12^ ^]^ These disorders cause substantial deterioration of motor functions, memory, and cognitive abilities.^[^ ^13^ ^]^ NDs are currently considered incurable due to irreversible neuronal degeneration.^[^ ^14^ ^]^ An estimated 55 million people worldwide are currently living with dementia, and this figure is expected to increase to 140 million by 2050. This growing prevalence places significant burdens on families and healthcare systems, particularly in low‐ and middle‐income countries.^[^ ^15^, ^16^ ^]^

Brain tumors, including primary brain tumors (such as glioblastoma (GBM), astrocytoma, and meningioma) and metastatic brain tumors originating from malignancies in other organs, profoundly affect patients’ quality of life.^[^ ^17^ ^]^ Each year, ≈330000 new cases of brain and CNS tumors are diagnosed globally, accounting for ≈ 1.8%–2.5% of all cancer cases.^[^ ^18^ ^]^ Among these, GBM accounts for 45%–50% of all brain tumors and is characterized by an alarmingly low 5‐year survival rate of 6%–10%, with a median survival of 12–18 months only, making it a critical focus of ongoing research.^[^ ^19^, ^20^, ^21^ ^]^ Brain metastases develop in ≈20% of cancer patients with cancer, commonly those with lung, breast, colon, or kidney cancer, and significantly worsen both prognosis and quality of life in individuals with advanced‐stage disease.^[^ ^22^ ^]^

Infectious and inflammatory brain diseases involve direct infection or inflammation of brain tissues triggered by pathogenic microorganisms or autoimmune reactions.^[^ ^23^ ^]^ Common examples include meningitis, encephalitis, and multiple sclerosis. Annually, bacterial meningitis accounts for ≈2.5 million cases globally. Moreover, encephalitis affects ≈4.2 million individuals each year, with viral encephalitis (caused by pathogens such as herpes simplex virus and Japanese encephalitis virus) being the most prevalent form.^[^ ^24^ ^]^

The treatment of traumatic brain injuries (e.g., concussion) and psychiatric disorders (e.g., schizophrenia and post‐traumatic stress disorder) typically necessitates multidisciplinary collaboration among neurologists, psychologists, and psychiatrists.^[^ ^25^, ^26^ ^]^ Given that approximately one in five adults experiences psychiatric disorders, mental health issues warrant increased attention and research investment.^[^ ^27^ ^]^

A major clinical challenge common to all brain disorders is the difficulty of delivering drugs effectively to the brain. This challenge underscores an urgent and unmet need for advanced therapeutic strategies that can ensure precise and targeted treatment.

### 2.2 Barriers to Therapeutic Access in the Brain

Effective treatment of brain disorders remains a significant clinical challenge, primarily due to intrinsic and extrinsic barriers restricting drug delivery and therapeutic efficacy. Understanding these challenges is crucial for advancing innovative approaches, such as micro‐ and nanorobotics, that aim to achieve precise and efficient drug delivery to the brain. This section discusses barriers to therapeutic access in the brain from three major perspectives: biological and pharmacological barriers, clinical and methodological barriers, and socioeconomic and ethical barriers.

**Figure 2:**
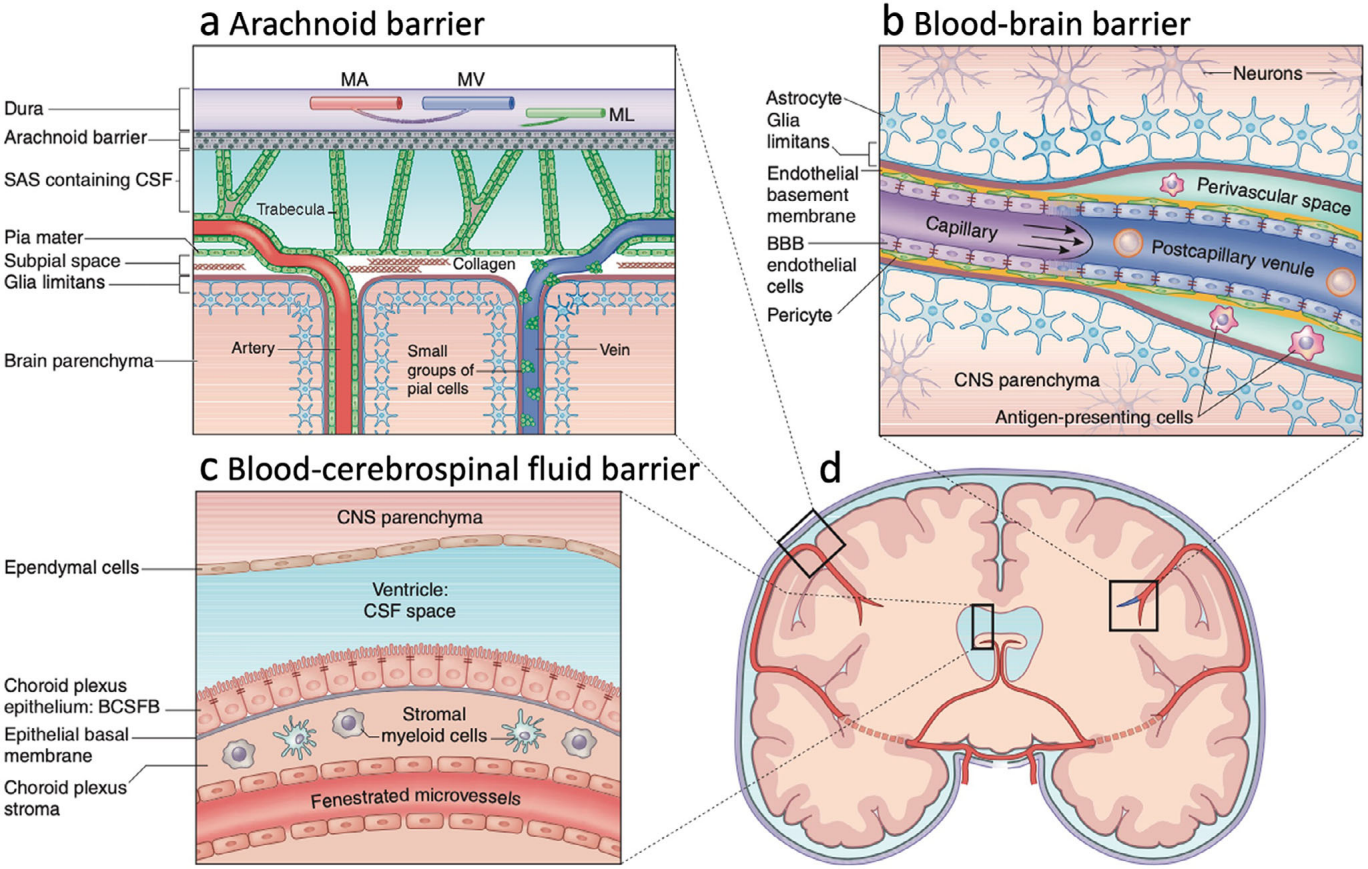
Biological barriers in the brain. a) AB. b) BBB. c) BCSFB. d) Coronal section of the brain depicting the location of brain barrier. Reproduced with permission from ref. [31] Copyright 2017, Springer Nature.

The biological and pharmacological barriers in the CNS significantly restrict drug penetration, distribution, and therapeutic efficacy. Among these barriers, the BBB, blood–cerebrospinal fluid barrier (BCSFB), and arachnoid barrier (AB, **Figure** **2**) serve as the primary physiological obstacles.^[^ ^28^, ^29^ ^]^ The BBB, composed of tightly interconnected endothelial cells, junctions, astrocyte end‐feet, pericytes, and the basal lamina, strictly regulates molecular transport, effectively restricting ≈98% of small‐molecule therapeutics and nearly all macromolecular agents from entering the brain parenchyma.^[^ ^30^ ^]^ Similarly, the BCSFB, primarily located at the choroid plexus epithelium, selectively controls the exchange of substances between the bloodstream and cerebrospinal fluid (CSF), significantly influencing the delivery of large therapeutic agents, including proteins, antibodies, and gene therapies.^[^ ^31^ ^]^ Formed by tight junctions within the arachnoid mater, the AB prevents free diffusion between the bloodstream and the subarachnoid CSF, further limiting drug access to the CNS.^[^ ^32^ ^]^

**Figure 3:**
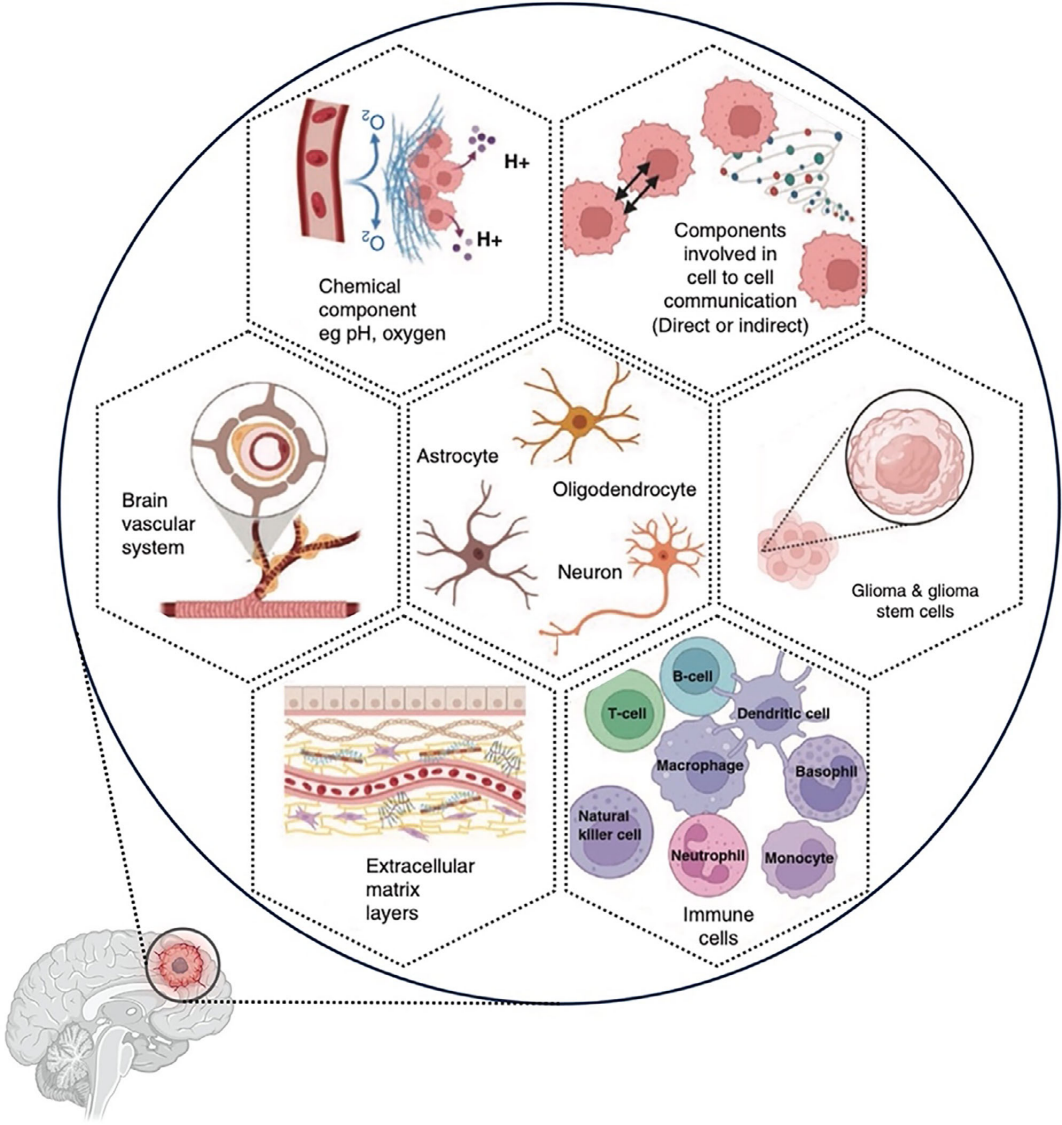
Changes in brain TME. Reproduced with permission from ref. [34] Copyright 2023, Oxford University Press.

In brain tumors, the compromised integrity of the BBB leads to the formation of the BBTB, characterized by abnormal vasculature, disrupted endothelial tight junctions, and heterogeneous permeability.^[^ ^33^ ^]^ Although the BBTB exhibits higher permeability than an intact BBB, it continues to significantly restrict drug accumulation in tumor tissues, thereby reducing therapeutic efficacy.^[^ ^28^ ^]^ Furthermore, the brain tumor microenvironment (TME) represents a complex and dynamically evolving ecosystem, characterized by distinct cellular and extracellular modifications that facilitate tumor growth, invasion, and resistance to therapy, as shown in **Figure** **3**.^[^ ^34^ ^]^ Key changes within TME include aberrations in chemical conditions such as decreased extracellular pH and altered oxygen concentration (hypoxia), which significantly influence tumor cell metabolism and therapeutic responsiveness.^[^ ^21^ ^]^ The intercellular communication within TME, mediated through direct cell‐cell contacts or indirect signaling via secreted factors, further promotes tumor progression and immune evasion.^[^ ^35^ ^]^ At the cellular level, TME comprises diverse populations, including malignant glioma cells, glioma stem cells, neurons, astrocytes, oligodendrocytes, and various immune cells (e.g., T cells, B cells, natural killer cells, dendritic cells, macrophages, neutrophils, basophils, and monocytes), each contributing unique and interactive roles in tumor biology.^[^ ^36^ ^]^ Structural components, including the brain vasculature and extracellular matrix (ECM) layers, undergo remarkable remodeling, resulting in compromised vascular integrity, altered permeability, and increased ECM stiffness, collectively facilitating tumor cell migration and invasion.^[^ ^37^ ^]^ Taken together, these multifaceted alterations in cellular composition, biochemical environment, and architectural framework modulate tumor behavior, thus influencing glioma progression and therapeutic responses.

The physicochemical properties of therapeutic agents (e.g., molecular size, hydrophilicity/lipophilicity balance, charge, and plasma half‐life) significantly influence their ability to traverse biological barriers, thereby critically affecting pharmacokinetics, pharmacodynamics, and ultimately their therapeutic efficacy.^[^ ^28^ ^]^

Significant practical and methodological challenges hinder the clinical translation of brain‐targeted therapies. The conventional delivery methods, such as systemic injection or oral administration, often suffer from poor brain specificity, limited therapeutic efficacy, and systemic toxicity. By contrast, invasive approaches like intrathecal or intracerebral injections can bypass the BBB but carry significant risks, including infection and tissue damage. Achieving precise spatial, temporal, and dosage control is complicated by disease heterogeneity, limitations in current imaging and guidance technologies, and inadequate preclinical models, all of which contribute to suboptimal treatment outcomes and hinder translational success. Promising therapeutic platforms often encounter additional challenges related to scalability, reproducibility, and long‐term safety, and the stringent requirements of regulatory approval.

Socioeconomic and ethical considerations also critically impact the clinical implementation of advanced brain‐targeted therapies. High costs associated with the development and deployment of MNR systems and other nanotechnology‐based approaches may limit accessibility, particularly in resource‐limited settings, thus widening global health disparities. Ethical and regulatory concerns, including patient safety, long‐term biocompatibility, uncertainties regarding biodegradation, and underdeveloped oversight frameworks, further delay clinical adoption. Public skepticism, limited awareness, and inadequate education about emerging technologies can hinder patient acceptance and stakeholder engagement, creating additional barriers to the widespread clinical integration of these technologies.

In summary, overcoming these multifaceted barriers requires comprehensive insights into biological, clinical, and societal challenges. Successfully addressing key biological constraints such as the BBB, the BCSFB, the BBTB, efflux transporters, enzymatic degradation, and TME, combined with optimized clinical methodologies and proactive management of socioeconomic and ethical considerations, could be instrumental in translating MNR‐based therapeutic platforms into clinically viable strategies for brain‐targeted therapies.

### 2.3 Pathways to Access the Brain

**Figure 4:**
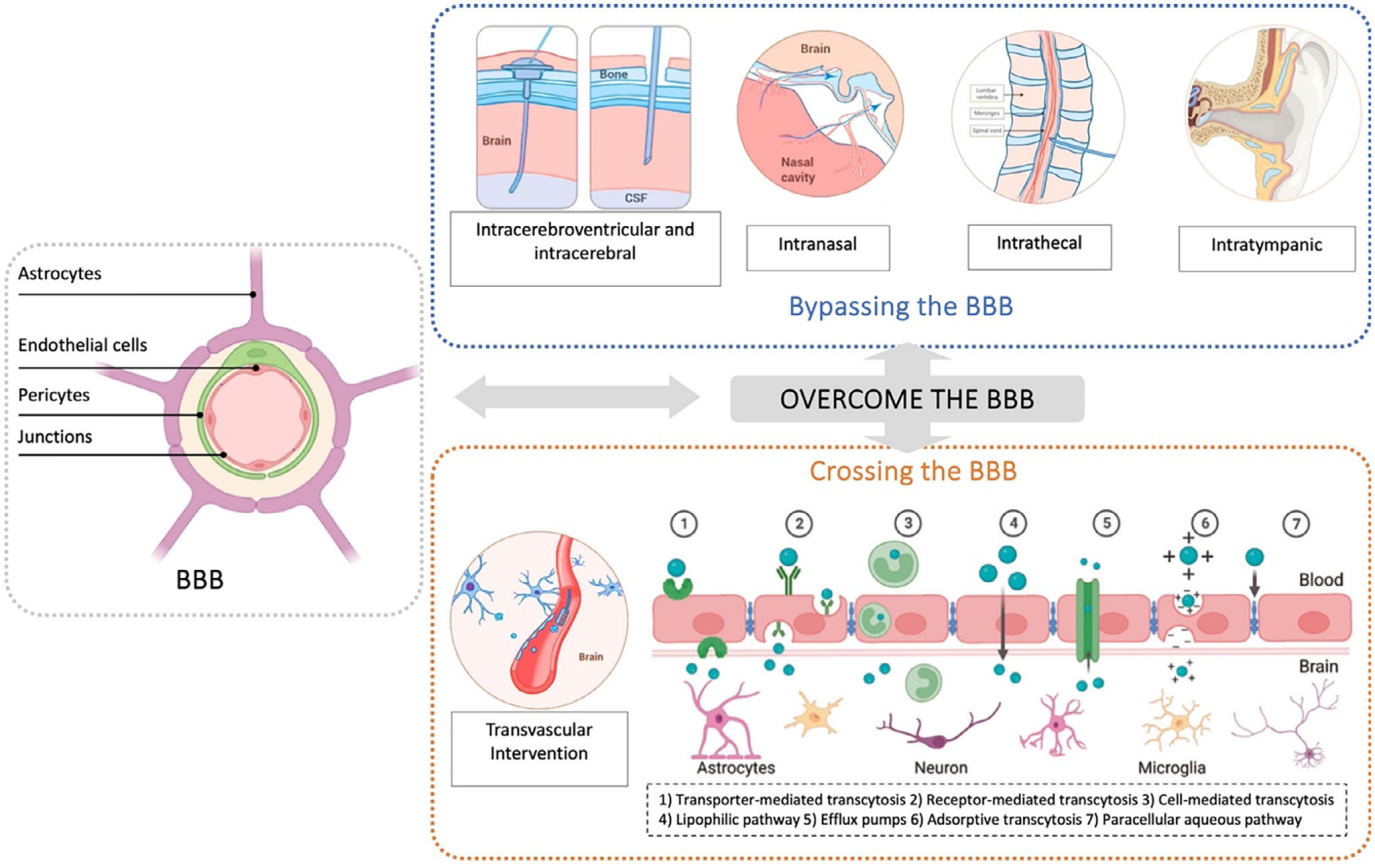
BBB structure and different mechanisms of overcoming the BBB, including bypassing BBB and crossing it. Reproduced with permission from the refs. [30, 38] and Copyright 2024, ScienceDirect, and Copyright 2023, Springer Nature. Some of the illustrations were created at BioRender.com.

The BBB is a dynamic and selectively permeable interface formed by specialized endothelial cells and tight junctions. While the BBB protects the CNS by preventing the entry of harmful substances from the bloodstream and maintaining neural homeostasis, it simultaneously poses a significant obstacle to the effective delivery of therapeutic agents to the brain. To overcome this challenge, various drug delivery strategies have been developed to overcome this challenge, broadly categorized into two principal approaches: bypassing the BBB and crossing it.^[^ ^32^ ^]^ The present review systematically summarizes the routes by which therapeutic agents access the CNS from two perspectives, as illustrated in **Figure** **4**.^[^ ^30^, ^38^ ^]^

Bypassing the BBB typically involves invasive administration methods that deliver therapeutic agents directly to the brain, regardless of the BBB's permeability mechanisms. Common bypass strategies include intracerebral administration (e.g., intraparenchymal injection), intraventricular injection, and peripheral routes such as intrathecal, intranasal, or intratympanic delivery.^[^ ^30^ ^]^ Although these methods are inherently invasive and may induce tissue damage, they have demonstrated significant clinical value in treating conditions, such as malignant brain tumors, subarachnoid hemorrhage (SAH), and PD, that require surgical intervention.

With the rapid advancements in materials science and nanotechnology and the growing understanding of BBB transport mechanisms, researchers have developed a range of strategies to modulate BBB permeability.^[^ ^28^ ^]^ These advancements have facilitated the development of diverse brain‐targeted drug delivery systems designed to facilitate effective transport across the BBB. Mechanisms leveraged by drug molecules to traverse the BBB include paracellular aqueous pathway, receptor‐mediated transcytosis, cell‐mediated transcytosis, transporter‐mediated transcytosis, lipophilic pathway, efflux pumps, and adsorptive transcytosis.^[^ ^35^ ^]^ Innovative brain‐targeted delivery platforms have been constructed using diverse materials, including organic carriers (e.g., liposomes, hydrogels),^[^ ^39^, ^40^ ^]^ inorganic nanoparticles (e.g., metallic particles, silica‐based nanoparticles),^[^ ^41^ ^]^ and biologically derived materials (e.g., exosomes and bacteria).^[^ ^42^, ^43^ ^]^ These delivery systems offer several advantages, including non‐invasive administration, high drug‐loading capacity, favorable biocompatibility, prolonged systemic circulation, and enhanced brain‐targeting capabilities.^[^ ^35^ ^]^ Collectively, these advancements represent significant progress in enhancing the efficacy and safety of brain‐targeted drug delivery, thereby opening promising avenues for the treatment of CNS disorders. The recent development and biomedical application of MNRs provide an additional strategy for targeted therapy of brain disorders, offering a compelling approach to overcome current therapeutic challenges.

## 3 MNR Categorization

As robotic sizes shrink to millimeter scales, volumetric forces become negligible compared with surface forces, and the integration of onboard batteries and sensors becomes challenging, significantly differentiating MNR design from conventional robotics.^[^ ^44^ ^]^ Specifically designed for biomedical applications, MNRs typically operate in liquid‐dominated environments, where their motion is predominantly influenced by fluid dynamics and external fields. Under these conditions, the Reynolds number (*Re*), defined as *Re* = ρ*Lv*/µ, where ρ refers to fluid density, µ denotes dynamic viscosity, *L* means characteristic length, and *v* indicates relative velocity, is typically below unity due to the robots’ small dimensions (10^−9^–10^−5^ m) and relatively low speeds.^[^ ^45^ ^]^ Consequently, viscous forces dominate, and fluid drag can be accurately approximated by Stokes’ law (*F_d_* = 3πµ*Lv*). External fields influencing robot motion include gravitational and driving fields, and gravity induces boundary interactions, such as friction (facilitating torque‐driven rolling) and electrostatic adhesion (hindering detachment from surfaces). Diverse driving fields, such as acoustic, optical, electric, and magnetic fields, provide propulsion, enabling MNRs to overcome viscous drag and boundary constraints, thus circumventing limitations dictated by the scallop theorem.^[^ ^46^, ^47^ ^]^ Propulsion via chemical reactions and biological microorganisms enables MNRs to harvest energy directly from their surroundings. Effective MNR design integrates these propulsion strategies to enable controlled locomotion in the presence of fluidic resistance and surface forces.

### 3.1 Chemical Propulsion

**Figure 5:**
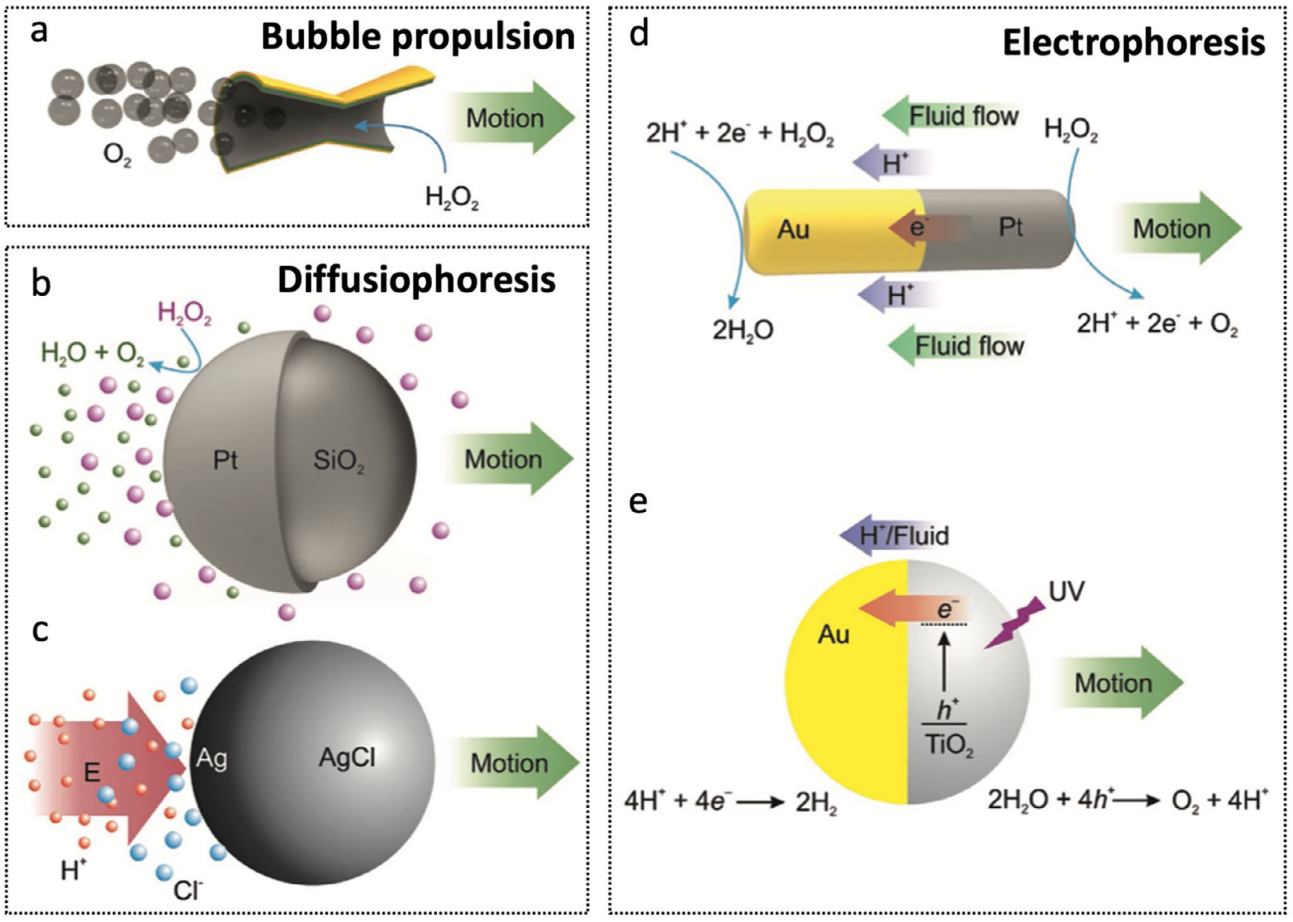
Chemical propulsion mechanisms, including bubble propulsion, diffusiophoresis, and electrophoresis. Reproduced with permission from ref. [48] Copyright 2018, Wiley‐VCH.

The chemical energy present in the surrounding environment can generate ion gradients and bubbles through reactions, providing the necessary mechanical energy for MNRs. As shown in **Figure** **5**, it includes three chemical driving principles: bubble propulsion (Figure 5a), diffusiophoresis (Figure 5b,c), and electrophoresis (Figure 5d,e).^[^ ^48^ ^]^

**Figure 6:**
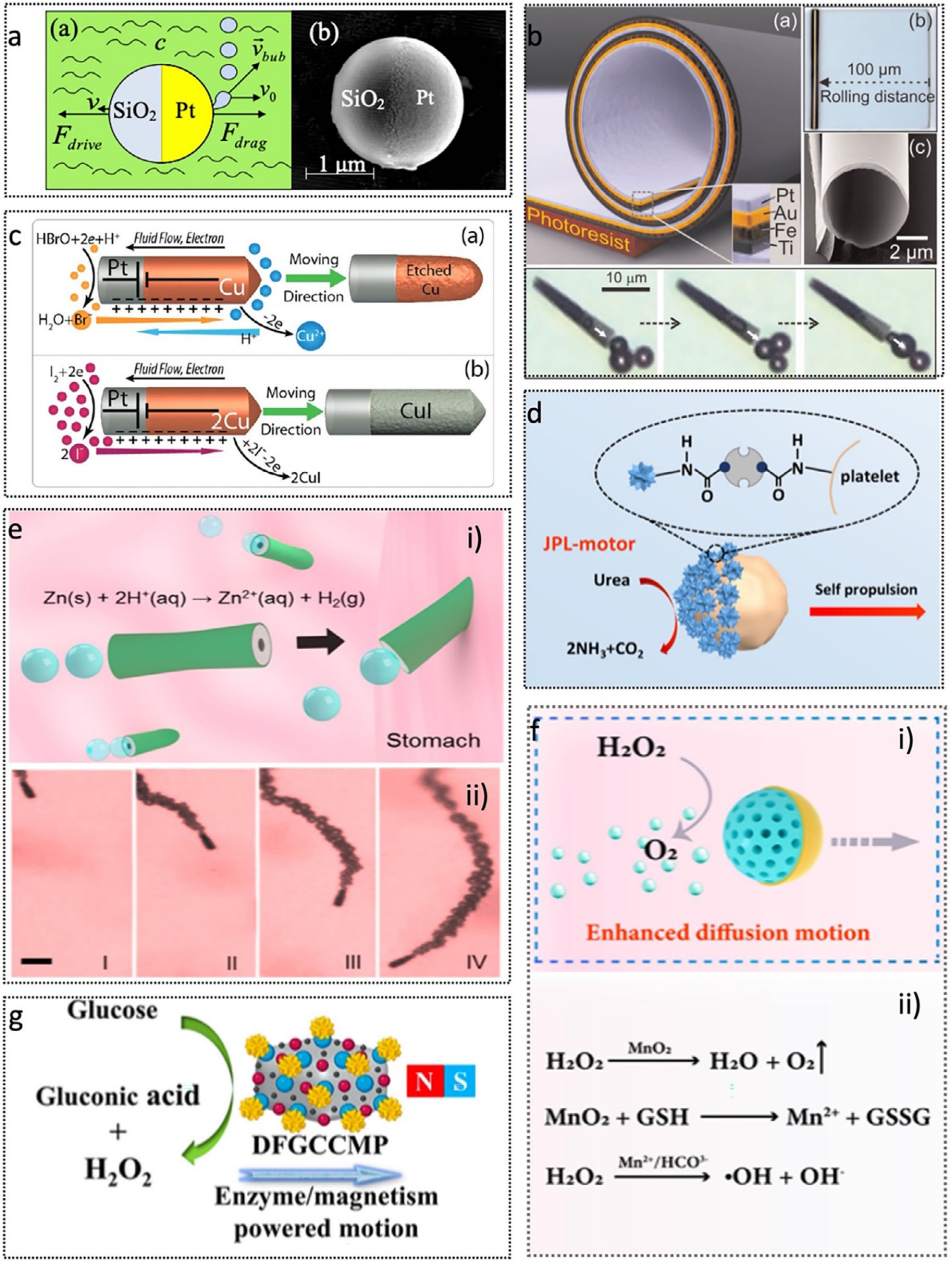
Chemical‐driven micro/nanorobots. a) Bubble‐propelled nanomotor driven by catalytic decomposition of H_2_O_2_ and momentum from detaching O_2_ bubbles. Reproduced with permission from ref. [52] Copyright 2009, American Institute of Physics Publishing. b) Diagram of a tubular microrobot leveraging catalytic bubble generation for directed release and ultra‐fast propulsion. Reproduced with permission from ref. [53] Copyright 2009, Wiley‐VCH. c) Bubble‐free Cu‐Pt segmented nanoscale motor functioning as a nanobattery in dilute Br_2_ or I_2_ solutions. Reproduced with permission from ref. [51] Copyright 2011, American Chemical Society. d) JPL‐motor fabricated by asymmetric urease immobilization on native platelet cells. Reproduced with permission from ref. [61] Copyright 2020, American Association for the Advancement of Science. e) PEDOT/Zn micromotor propelled by gastric acid: i) in vivo propulsion and tissue penetration in a mouse stomach and ii) time‐lapse images of propulsion in gastric acid at physiological temperature. Scale bar, 20 µm. Reproduced with permission from ref. [65] Copyright 2015, American Chemical Society. f) Self‐propelled biodegradable hollow MnO_2_ nanomotor driven by endogenous hydrogen peroxide, highlighting (i) enhanced diffusion motion and (ii) reaction mechanism. Reproduced with permission from ref. [66] Copyright 2021, American Chemical Society. g) Enzyme/magnetic micromotor designed for synergistic cancer therapy. Reproduced with permission from ref. [59] Copyright 2021, American Chemical Society.

These MNRs primarily exist in three morphological forms: microspheres, microtubes, and nanowires.^[^ ^49^, ^50^, ^51^, ^52^, ^53^, ^54^, ^55^ ^]^ These structures predominantly appear as microspheres, microtubes, or nanowires. Among them, Janus microspheres employ various propulsion mechanisms, notably bubble propulsion and self‐diffusiophoresis. In self‐diffusiophoresis, motion is driven by concentration gradients of solutes generated through the catalytic decomposition of hydrogen peroxide (H_2_O_2_). By contrast, bubble propulsion arises from the recoil force produced by the asymmetric release of oxygen bubbles during H_2_O_2_ decomposition. As shown in **Figure** **6**a, a catalytic nanomotor demonstrates bubble‐driven propulsion, where its movement results from momentum transfer associated with the detachment of O_2_ bubbles from the catalyst surface.^[^ ^52^ ^]^ This mechanism overcomes the limitations of ion‐gradient‐based propulsion, making it more suitable for use in biological fluids. Figure 6b illustrates the fabrication process and delayed motion images of tubular MNRs, which effectively utilize bubbles generated from catalytic reactions and linearly release them, thereby enabling ultrafast motion.^[^ ^53^ ^]^ In 2004, the first self‐electrophoretic Au/Pt bimetallic nanowire motor was reported.^[^ ^55^ ^]^ Subsequently, other fuel sources such as hydrazine and dilute solutions of I_2_ and Br_2_ (Figure 6c), were developed to achieve self‐propulsion of nanowires.^[^ ^51^ ^]^ However, chemically powered MNRs utilizing the above fuels present toxicity concerns and potential side effects when employed within biological tissues.^[^ ^56^ ^]^ Researchers have developed MNRs based on biocompatible and biodegradable materials to enhance their suitability for in vivo applications, and these MNRs can utilize biological fluids such as gastric acid, intestinal fluids, and enzymes as fuel sources.^[^ ^57^, ^58^, ^59^, ^60^, ^61^, ^62^, ^63^, ^64^ ^]^

Tang et al. developed an endogenously enzyme‐driven Janus platelet micromotor (JPL‐motor), as illustrated in Figure 6d.^[^ ^61^ ^]^ The micromotor was fabricated by selectively immobilizing urease onto one side of natural platelet cells, enabling asymmetric enzymatic decomposition of urea present in biological fluids. This enzymatic reaction generated enhanced chemotactic propulsion while notably preserving the intrinsic biological properties of platelets, including their inherent targeting capabilities toward cancer cells and bacteria. Gao et al. introduced a PEDOT/Zn‐based micromotor (Figure 6e) exhibiting efficient acid‐driven propulsion within the gastric environment.^[^ ^65^ ^]^ This propulsion mechanism facilitated improved micromotor adhesion and prolonged retention on gastric mucosal surfaces, along with its therapeutic payload. Significantly, the micromotor's body gradually dissolved upon exposure to gastric acid, autonomously releasing its cargo without generating toxic residues. As depicted in Figure 6f, researchers successfully fabricated biodegradable, self‐propelled nanomotors on the basis of hollow MnO_2_ nanoparticles, utilizing endogenous hydrogen peroxide as fuel for autonomous movement.^[^ ^66^ ^]^ Upon cellular internalization, the nanomotor consumes intracellular overexpressed glutathione (GSH), leading to the generation of toxic Fenton‐like Mn^2^⁺ ions. Considering that GSH plays a crucial role in scavenging hydroxyl radicals (·OH), its depletion results in an enhanced cancer chemodynamic therapy (CDT). As depicted in Figure 6g, researchers have developed an enzyme/magnetic micromotor for synergistic cancer therapy.^[^ ^59^ ^]^ The enzymatic reaction catalyzed by glucose oxidase employs glucose as a fuel to achieve autonomous propulsion through a chemical engine, whereas Fe_3_O_4_ nanoparticles serve as magnetic actuators to enhance kinetic performance and enable directional control. Despite significant advancements in chemically propelled MNRs for biomedical applications, several critical challenges persist. These include immune responses in the bloodstream, the inhibitory effects of high ionic strength on propulsion, the high fuel consumption requirements, toxicity concerns, and the elimination of byproducts.^[^ ^67^ ^]^ Additionally, the impact of localized fuel depletion on the physiological environment must be carefully considered.^[^ ^68^ ^]^

### 3.2 External‐Field Propulsion

#### 3.2.1 Acoustic Field

The frequency range of ultrasound spans from 20 kHz to 10 MHz. Ultrasound represents a non‐contact, wireless, and safe actuation method for MNRs owing to its strong penetrability and excellent biocompatibility, offering promising clinical applications that complement magnetic field‐based approaches.^[^ ^69^ ^]^ Acoustic fields are generally classified into standing waves and traveling waves, both of which have been extensively employed in the development of acoustic MNRs for biomedical applications.^[^ ^70^, ^71^, ^72^, ^73^ ^]^ Standing waves are formed by the interference of two opposite waves and appear stationary with fixed nodes and antinodes, whereas traveling waves move through a medium, continuously transferring energy from one point to another.^[^ ^74^ ^]^

**Figure 7:**
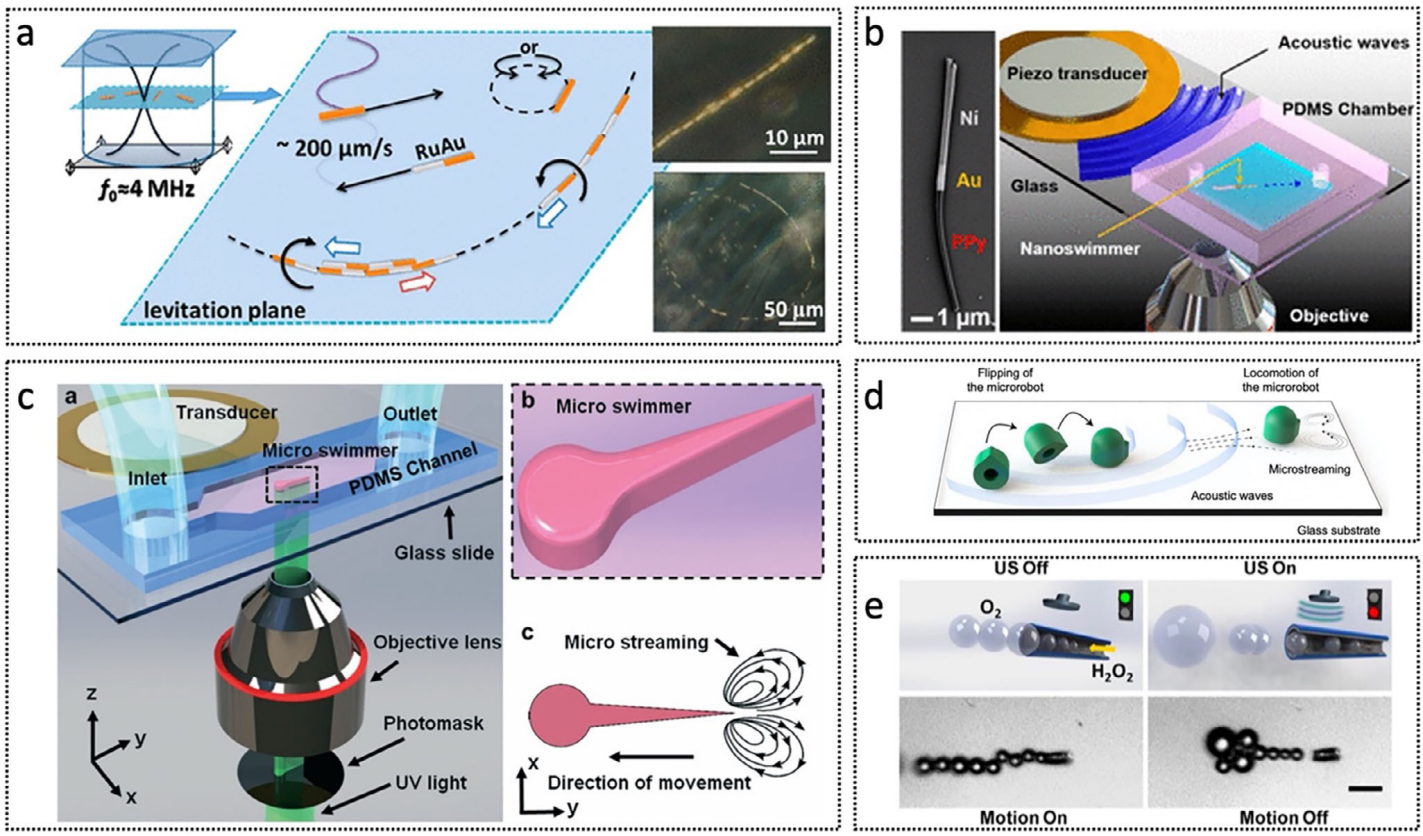
Acoustic‐driven micro/nanorobots. a) Acoustic metallic microrods aligning and self‐assembling into dynamic spinning chains with characteristic patterns, including ring and streak patterns. Reproduced with permission from ref. [75] Copyright, 2012. American Chemical Society. b) Nanoswimmer with a rigid bimetallic head and a flexible tail propelled by acoustically activated flagella. Reproduced with permission from ref. [78] Copyright, 2016. American Chemical Society. c) Fabrication and acoustic actuation of microswimmers. Reproduced with permission from ref. [79] Copyright, 2016. Royal Society of Chemistry. d) Acoustically driven bullet‐shaped microrobot. Reproduced with permission from ref. [81] Copyright, 2020. National Academy of Sciences. e) Ultrasound‐modulated bubble propulsion of chemically powered microengines. Scale bar, 20 µm. Reproduced with permission from ref. [82] Copyright, 2014. American Chemical Society.

Considering that the forces exerted by standing waves are significantly stronger than those generated by traveling waves, many studies have focused on utilizing standing wave fields for MNR actuation.^[^ ^74^, ^75^, ^76^, ^77^ ^]^ Wang et al. demonstrated that ultrasonic standing waves can effectively levitate, propel, and precisely assemble metallic microrods in water and solutions with high ionic strength, addressing limitations inherent to conventional phoretic micromotors.^[^ ^75^ ^]^ The microrods exhibit fast, directional motion driven by a proposed self‐acoustophoresis mechanism arising from their structural asymmetry. The rods align and self‐assemble into dynamic spinning chains with characteristic patterns, including ring and streak patterns (**Figure** **7**a). MNRs driven by standing waves rely on precisely designed standing wave acoustic fields, which are difficult to achieve in vivo. Therefore, traveling waves are widely used to propel micro/nanomotors. A nanoswimmer comprising a rigid bimetallic head and a flexible tail (Figure 7b) attained efficient propulsion through tail oscillations under standing and traveling acoustic wave conditions.^[^ ^78^ ^]^ Unlike conventional designs, it exploits structural resonance to enhance thrust, making it particularly promising for in vivo applications where establishing standing waves can be challenging. Building on this concept, artificial flagella, as shown in Figure 7c, have been engineered to enable translational and rotational motion.^[^ ^79^ ^]^ When the flagellum and body are symmetric, oscillations produce directional movement along the axis of symmetry. Breaking this symmetry along the midline yields rotational swimmers, pre‐programmed to rotate clockwise or counterclockwise depending on the flagellum's placement.

In addition to propulsion mechanisms driven by standing and traveling acoustic waves, MNRs can be actuated through acoustic droplet vaporization, enabling exceptionally high velocities of up to 6.3 m s^−1^.^[^ ^80^ ^]^ Another type of acoustically propelled bullet‐shaped microrobot (Figure 7d) incorporates magnetic fields to enable high‐speed, directional locomotion across planar and curved surfaces, achieving velocities of up to 90 body lengths per second.^[^ ^81^ ^]^ Propelled by oscillating bubbles within an internal cavity and guided by magnetic steering, these microrobots exhibit precise surface locomotion in confined environments. Moreover, ultrasound has been employed in conjunction with chemically propelled MNRs to optimize their motion behavior, enabling controllable and tunable propulsion modes (Figure 7e).^[^ ^82^ ^]^ However, geometry‐dependent motion, high power requirements, and the potential risk of tissue damage limit the biomedical applications of acoustic‐based MNRs. These challenges remain critical issues that must be addressed before their practical implementation.

#### 3.2.2 Light Field

Recent progress in light‐driven MNRs has significantly advanced the development of untethered microsystems, enabling the precise execution of complex tasks with superior spatial and temporal control.^[^ ^83^ ^]^ On the basis of their fabrication methodologies, these MNRs can be classified into three distinct categories: hard, soft, and nonpolymeric robots.^[^ ^84^ ^]^ Each category offers specific advantages in terms of manufacturing processes, functional properties, and potential applications.

**Figure 8:**
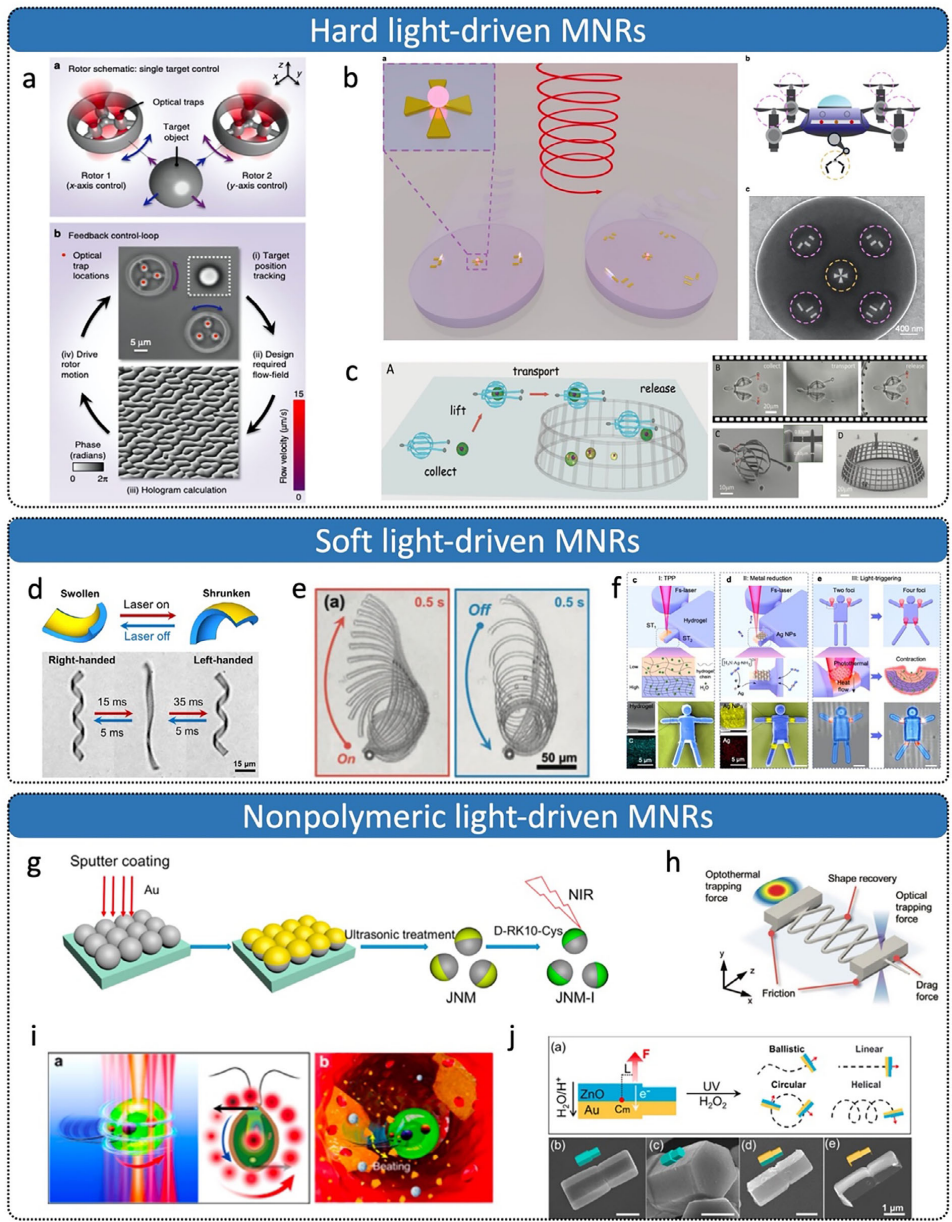
Light‐driven micro/nanorobots. a) Light‐driven microrotors using indirect optical trapping for reconfigurable hydrodynamic manipulation. Reproduced with permission from ref. [90] Copyright 2019, Springer Nature. b) Light‐driven plasmonic microrobot for nanoparticle manipulation. Reproduced with permission from ref. [91] Copyright 2025, Springer Nature. c) Single‐cell collection with optically actuated microstructure. Reproduced with permission from ref. [89] Copyright 2024, Wiley‐VCH. d) Helical microgel ribbons. Reproduced with permission from ref. [99] Copyright 2017, American Chemical Society. e) Laser‐driven ribbon‐like microrobot. Reproduced with permission from ref. [100] Copyright 2019, Wiley‐VCH. f) Light‐triggered multi‐joint microactuator. Reproduced with permission from ref. [101] Copyright 2023, Springer Nature. g) Near‐infrared light‐powered Janus nanomotor. Reproduced with permission from ref. [106] Copyright 2020, American Chemical Society. h) Laser‐controlled microrobot based on nickel‐titanium shape memory alloy. Reproduced with permission from ref. [103] Copyright 2019, Wiley‐VCH. i) Optically controlled living micromotor. Reproduced with permission from ref. [104] Copyright 2020, American Chemical Society. j) Light‐active ZnO/Au twinned rods. Reproduced with permission from ref. [105] Copyright 2020, American Chemical Society.

Hard MNRs are typically fabricated via two‐photon polymerization (2PP) using direct laser writing (DLW), which enables the creation of high‐resolution, complex 3D nanoscale structures.^[^ ^85^ ^]^ These robots are rigid and non‐deformable, with their geometry and function defined during fabrication. They are commonly manipulated using optical trapping techniques, such as optical or optoelectronic tweezers, allowing precise multi‐degree‐of‐freedom control.^[^ ^86^, ^87^, ^88^ ^]^ This class is particularly suited for tasks requiring high precision, such as directional manipulation in microfluidics or single‐cell handling.^[^ ^89^ ^]^ For example, a light‐driven micro‐rotor (**Figure** **8**a) employing indirect optical trapping achieved nanoscale control of aqueous particles through hydrodynamic interactions, avoiding direct laser exposure on biological specimens.^[^ ^90^ ^]^ Qin et al. integrated a plasmonic nano‐tweezer into a microrobot (Figure 8b), enabling all‐optical trapping and delivery of individual nanoparticles in solution, with potential applications in targeted drug delivery and cell manipulation.^[^ ^91^ ^]^ Gergely et al. proposed a series of optically deformable cell micromanipulators (Figure 8c) capable of securing cells without biochemical functionalization.^[^ ^89^ ^]^ However, the serial nature of 3D printing limits scalability, and its use is often confined to well‐controlled laboratory conditions.

By contrast, soft MNRs consist of stimuli‐responsive materials, such as hydrogels or liquid crystal elastomers, capable of reversible shape transformations upon exposure to light.^[^ ^92^, ^93^, ^94^, ^95^, ^96^, ^97^, ^98^ ^]^ These materials enable autonomous, dynamic motion through self‐actuation, mimicking the motion of biological systems. Light‐induced deformation is typically triggered via photothermal effects, allowing diverse movements such as crawling, swimming, or grasping.^[^ ^93^ ^]^ For instance, a PNIPAM‐based hydrogel strip coated with a thin gold film (Figure 8d) exhibited helical curling and reversal of direction in response to temperature‐induced volume transitions.^[^ ^99^ ^]^ Another 2D helical structure rotates via non‐reciprocal deformation (Figure 8e).^[^ ^100^ ^]^ Inspired by human joints, a hydrogel–metal nanoparticle hybrid micro‐joint (Figure 8f) exhibited multimodal deformation with a fast response time (∼30 ms) and low power consumption (<10 mW).^[^ ^101^ ^]^ The adaptability and biocompatibility of soft robots position them as promising candidates for biomedical applications, particularly in minimally invasive surgical procedures and targeted therapeutic interventions.^[^ ^102^ ^]^ However, challenges remain in achieving sufficient mechanical output and reliable performance in complex biological environments.

Nonpolymeric MNRs encompass a diverse range of light‐responsive systems, including Janus particles, metal‐based microswimmers, and biohybrid constructs.^[^ ^103^, ^104^, ^105^, ^106^, ^107^ ^]^ Unlike polymeric robots, these systems rely on asymmetric surface properties or photocatalytic reactions for propulsion. For example, a near‐infrared (NIR)‐driven Janus nanomotor (JNM‐I) functionalized with inhibitors (Figure 8g) was designed to modulate amyloid‐β aggregation associated with AD.^[^ ^106^ ^]^ Janus structures offer simple geometry and scalability but often lack precise controllability. Other examples include a shape‐memory alloy‐based microrobot (Figure 8h) actuated by laser scanning[104] and ZnO/Au rods exhibiting a transition from ballistic to rotational motion under UV light as the fuel concentration or light intensity increases (Figure 8i).^[^ ^104^ ^]^ Additionally, a biohybrid micromotor employing Chlamydomonas reinhardtii (Figure 8j) exhibited precise, light‐guided locomotion across various biological media, including culture medium, saliva, serum, blood, and bone marrow.^[^ ^105^ ^]^

In summary, the development of light‐driven MNRs involves a wide range of material systems and actuation strategies. Specific application requirements should guide the choice among hard, soft, or nonpolymeric designs. Ongoing interdisciplinary efforts aim to integrate the strengths of each category while addressing current limitations, advancing the practical deployment of these systems.

#### 3.2.3 Electric Field

Electric actuation, as an emerging external‐field‐driven approach, has attracted increasing attention owing to its advantages, including low cost, contact‐free manipulation, and ease of integration.^[^ ^85^ ^]^ Similar to magnetic actuation, which relies on the magnetic properties inherent to materials, electric actuation leverages the inherent charges or polarization of materials to achieve controllable motion under external electric fields.^[^ ^108^ ^]^ Depending on the type of electric field and the underlying propulsion mechanism, electrically driven MNRs can utilize various modes, including electrophoresis, electroosmosis, dielectrophoresis, and electrically induced chemical reactions.^[^ ^109^, ^110^, ^111^, ^112^, ^113^, ^114^, ^115^, ^116^, ^117^ ^]^ These mechanisms can be activated by direct current (DC) or alternating current (AC) electric fields. Among these, AC electric fields offer richer control possibilities and enhanced adaptability, making them particularly suitable for micro/nano‐manipulation in complex biofluid environments.^[^ ^70^ ^]^

**Figure 9:**
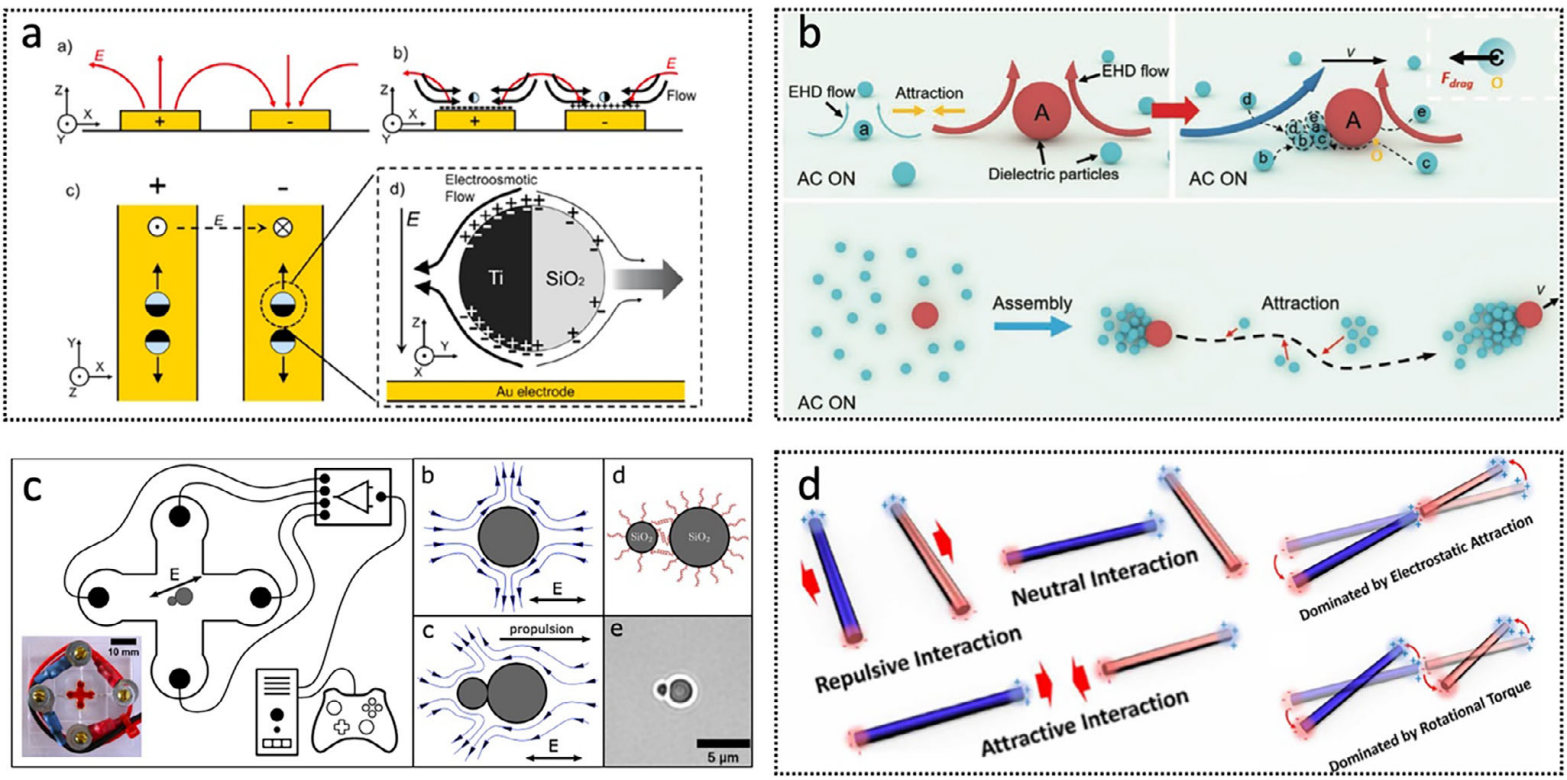
Electric‐driven micro/nanorobots. a) Metallodielectric Janus micromotors under AC electric fields. Reproduced with permission from ref. [114] Copyright 2019, American Chemical Society. b) Hierarchical microswarms with leader‐follower structures. Reproduced with permission from ref. [113] Copyright 2020, Wiley‐VCH. c) Microrobots powered by concentration polarization electrophoresis (CPEP). Reproduced with permission from ref. [111] Copyright 2023, Springer Nature. d) System composed of optoelectronic semiconductor nanorods can rapidly deform into three distinct modes under electric field light stimulation: network formation, collective rotation enhancement, and droplet‐like clustering. Reproduced with permission from ref. [110] Copyright 2023, American Association for the Advancement of Science.

MNRs actuated by AC electric fields demonstrate diverse propulsion behaviors. For instance, Janus particles (**Figure** **9**a) exhibit motion perpendicular to the direction of the applied electric field through induced charge electrophoresis, offering more sophisticated manipulation capabilities.^[^ ^114^ ^]^ Researchers observed that dielectric particles of varying sizes can self‐organize into micro‐swarms with leader–follower structures via electrohydrodynamic interactions under AC electric fields, indicating the potential of electric actuation in swarm intelligence (Figure 9b).^[^ ^113^ ^]^ In 2023, Katzmeier et al. developed asymmetric particle dimers (Figure 9c), whose movement can be precisely controlled by concentration polarization electroosmosis along the electric field lines.^[^ ^111^ ^]^ Under low‐ionic‐strength media and AC electric fields, symmetric fluid flows develop around these asymmetric particle dimers. Due to orientation torques induced by dipoles and fluid flows, dimers align with the external electric field and are propelled by electroosmotic flows around them. Liang et al. (Figure 9d) demonstrated the assembly and disassembly of micromotors via the combination of electrorotation and near‐field electrostatic forces.^[^ ^110^ ^]^ Electric actuation can be integrated with other external fields (such as light, magnetic, or biological fuels) to expand functionality further. For example, graphene‐based helical microrobots not only move under electric fields but also enable drug release via electrochemical stimulation, providing new avenues for precision medicine.^[^ ^118^ ^]^

However, current research on electrically driven MNRs remains restricted to in vitro studies.^[^ ^119^ ^]^ In physiological environments with high ionic strength, such as blood, electric fields are readily screened, substantially reducing propulsion efficiency. High voltages may cause adverse effects such as medium heating and electrolysis, thus restricting practical in vivo applications. Therefore, future studies should focus on optimizing actuation mechanisms, electric‐field design, electric‐material coupling strategies, and adaptability to microenvironments, thus advancing electrically driven MNRs toward clinical applications.

#### 3.2.4 Magnetic Field

The fundamental principle underlying magnetic actuation involves exerting forces or torques on magnetized micro‐ and nanoscale objects.^[^ ^46^, ^70^ ^]^ When placed in an external magnetic field ***B*** [*T*], a magnetized object experiences a torque ***T*** *_m_* [*N* · *m*], calculated by ***T*** *_m_* = *V* ***M*** × ***B***, that aligns its magnetization vector ***M*** [*A* · *m* ^−1^] with the applied field direction. The magnetic force ***F*** *_m_* [*N*] exerted on the object is expressed as **F**_m_ = *V*(***M*** ∇) ***B***, where *V* [*m* ^3^] represents the volume of the magnetic object.

**Figure 10:**
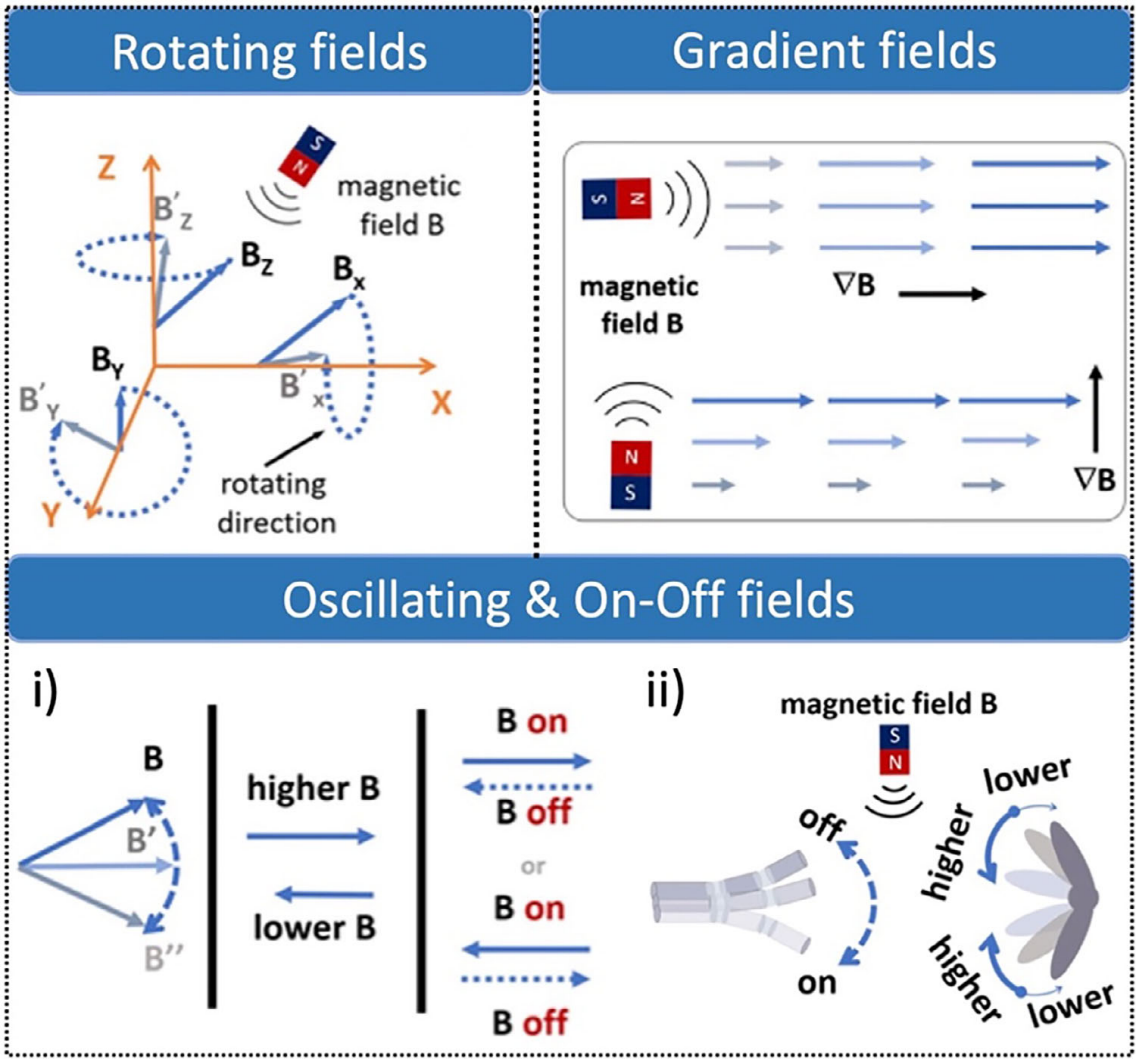
Actuation methods via rotating magnetic fields, gradient magnetic fields, and oscillating and pulsed (on–off) magnetic fields. Reproduced with permission from ref. [83] Copyright 2023, Royal Society of Chemistry.

Furthermore, an object may exhibit permanent magnetization or display soft magnetic, paramagnetic, or superparamagnetic characteristics, becoming magnetized only when an externally applied magnetic field is present. Consequently, the magnetization ***M*** can be constant for hard magnetic materials or vary depending on the applied magnetic field and the object's geometry. In a uniform magnetic field, a micro‐object experiences no net force but rather a torque, causing alignment of ***M*** with the magnetic field ***B***. Once aligned, the torque ***T*** *_m_* becomes zero, and the micro‐object remains stationary. The applied magnetic field must either (1) vary spatially, creating a magnetic field gradient, or (2) vary temporally, through rotation, oscillation, or periodic switching, to sustain continuous actuation, as shown in **Figure** **10**.

**Figure 11:**
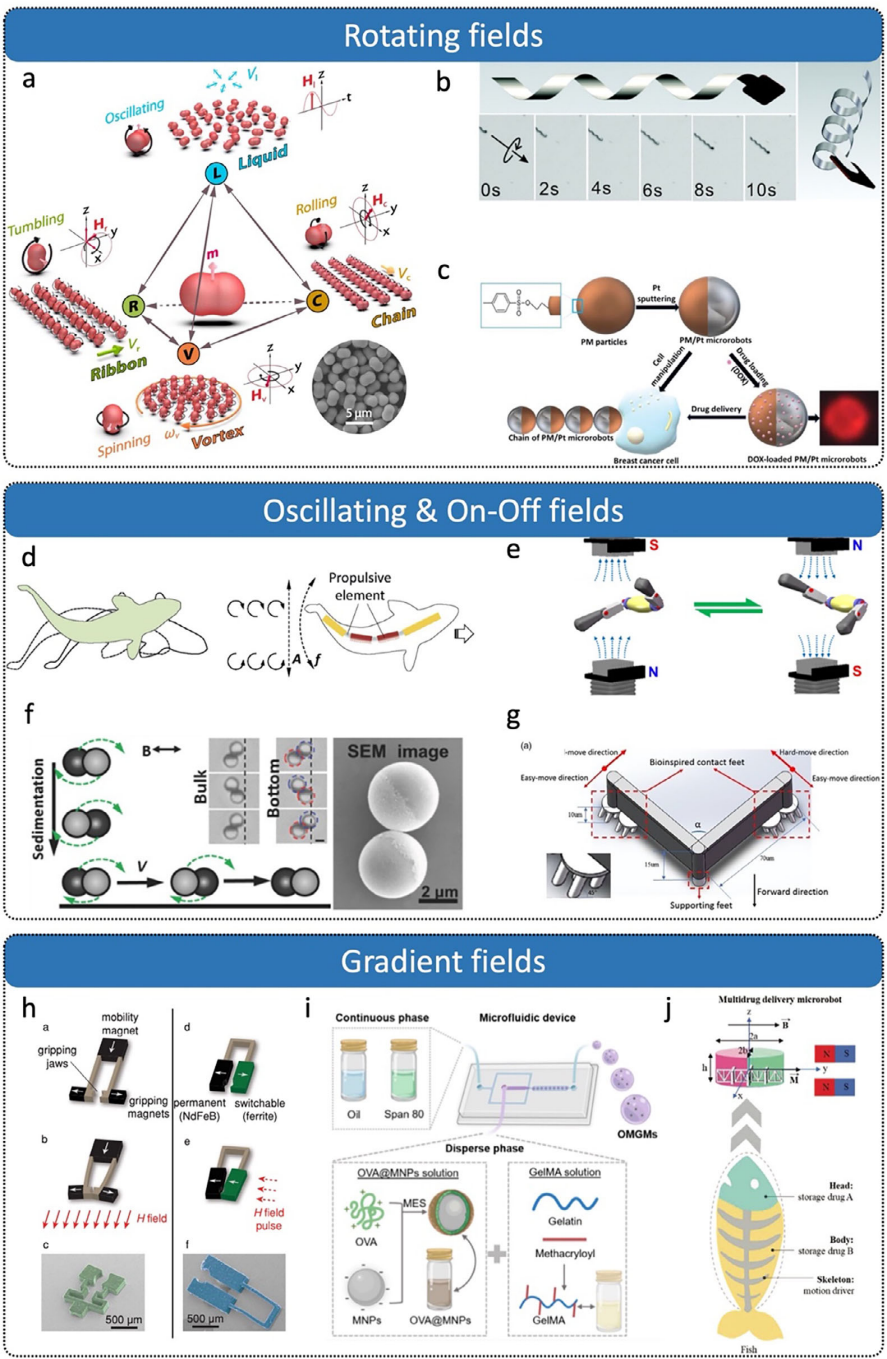
Magnetic‐driven micro/nanorobots. a) Swarm of peanut‐shaped microrobots transforms under different magnetic fields. Reproduced with permission from ref. [121] Copyright 2019, American Association for the Advancement of Science. b) Bio‐inspired rotating artificial bacterial flagella. Reproduced with permission from ref. [120] Copyright 2009, American Institute of Physics Publishing. c) PM/Pt magnetic rotating microrobots. Reproduced with permission from ref. [122] Copyright 2018, Wiley‐VCH. d) Segmented magnetically actuated nanoswimmer mimicking fish locomotion. Reproduced with permission from ref. [128] Copyright 2016, Wiley‐VCH. e) Symmetric multilinked two‐arm nanoswimmer. Reproduced with permission from ref.[131] Copyright 2017, American Chemical Society. f) Janus microdimer surface walkers. Reproduced with permission from ref. [133] Copyright 2018, Wiley‐VCH. g) Magnet‐driven microwalker in surface motion based on frictional anisotropy. Reproduced with permission from ref. [132] Copyright 2022, Wiley‐VCH. h) Untethered magnetic robotic micro‐gripper. Reproduced with permission from ref. [134] Copyright 2014, Wiley‐VCH. i) Magnetically driven biodegradable antigen delivery microsphere. Reproduced with permission from ref. [135] Copyright 2024, American Chemical Society. j) Elliptical multidrug delivery magnetic microrobot. Reproduced with permission from ref. [136] Copyright 2023, Wiley‐VCH.

First, researchers have developed various MNRs driven by rotating magnetic fields (RMFs), including peanut‐shaped, bio‐inspired helical, and Janus spherical designs.^[^ ^120^, ^121^, ^122^, ^123^, ^124^, ^125^ ^]^ Magnetic micro/nano‐objects can move under appropriate magnetic fields even in non‐Newtonian fluids or near boundaries.^[^ ^45^ ^]^ As shown in **Figure** **11**a, peanut‐shaped swimming microrobots can dynamically reconfigure their collective formations under different RMFs, enabling navigation through complex narrow environments and cooperative cargo delivery.^[^ ^121^ ^]^ Another extensively studied asymmetric design inspired by bacterial flagella features a helical structure (Figure 11b), consisting of a thin, soft magnetic head and a helical tail.^[^ ^120^ ^]^ Under RMFs, these structures rotate helically around their long axis, generating propulsion perpendicular to the rotation plane. The propulsion force increases with the applied magnetic field frequency, enabling 3D swimming once sufficient thrust is achieved.^[^ ^126^, ^127^ ^]^ Additionally, Janus spherical structures have become a popular choice for fabricating magnetic MNRs. For instance, multifunctional spherical superparamagnetic/catalytic microrobots (termed PM/Pt magnetic microrobots, Figure 11c) have been developed for targeted delivery tasks under RMFs.^[^ ^122^ ^]^

Second, oscillating or switching magnetic fields have been widely employed to actuate various MNRs, such as multilink swimmers and Janus hybrid peanut‐shaped structures.^[^ ^128^, ^129^, ^130^, ^131^, ^132^ ^]^ As shown in Figure 11d, a multilink artificial microswimmer propels forward by generating backward‐traveling waves through undulatory motion.^[^ ^128^ ^]^ The swimmer was constructed from two gold segments, which function as the head and tail fins, and two nickel segments that form the central body. Three flexible silver hinges interconnect these components. When subjected to oscillating magnetic fields, the periodic bending of nickel segments generates a wave‐like propulsion. Figure 11e demonstrates a symmetric, dual‐arm multilink nanoswimmer capable of efficient “freestyle” swimming under low Reynolds number conditions.^[^ ^131^ ^]^ Driven by planar oscillating magnetic fields, the cooperative non‐planar strokes of its two magnetic arms yield propulsion speeds up to 12 body lengths per second. Ni‐SiO_2_ Janus microsphere dimers (Figure 11f) roll across surfaces under oscillating magnetic fields.^[^ ^133^ ^]^ Additionally, a surface‐walking microrobot leveraging friction anisotropy through locally patterned contact interfaces (Figure 11g) can adapt to varying environments, such as steep inclines, under oscillating magnetic fields.^[^ ^132^ ^]^

Third, gradient magnetic fields are another popular choice owing to their relatively simple structural requirements, suitable for spherical, elliptical, or specially assembled micro/nanostructures.^[^ ^134^, ^135^, ^136^, ^137^, ^138^, ^139^, ^140^ ^]^ For instance, flexible patterned magnetic materials driven internally by gradient fields have been developed into mobile microgrippers (Figure 11h).^[^ ^134^ ^]^ Mass‐produced biodegradable magnetic microspheres (Figure 11i) have been successfully applied in the delivery of subunit antigen vaccines.^[^ ^135^ ^]^ Elliptical, fishbone‐shaped microrobots fabricated via 3D printing (Figure 11j) can perform drug delivery tasks under a gradient magnetic field.^[^ ^136^ ^]^

### 3.3 Biohybrid Propulsion

In recent years, biohybrid MNRs have garnered significant attention as a promising approach to addressing the limitations of conventional synthetic microrobots, particularly in terms of biocompatibility, biodegradability, and autonomous motion control.^[^ ^141^, ^142^ ^]^ These systems integrate artificially fabricated micro/nanostructures with the intrinsic properties of biological entities, utilizing the natural motility; biocompatibility; and sensing capabilities of cells,^[^ ^143^, ^144^, ^145^, ^146^ ^]^ bacteria,^[^ ^147^, ^148^ ^]^ sperm,^[^ ^149^, ^150^, ^151^ ^]^ and microalgae^[^ ^104^, ^152^, ^153^, ^154^, ^155^, ^156^, ^157^ ^]^ to achieve more efficient navigation and functional execution. Specifically, biohybrid MNRs are formed by coupling biological components with artificial materials through physical entrapment, covalent or noncovalent interactions, or cellular internalization.^[^ ^158^ ^]^ For instance, natural propulsion and sensing mechanisms such as sperm flagellar beating, bacterial chemotaxis, and algal phototaxis endow these robots with excellent adaptability and responsiveness to biological environments.^[^ ^159^, ^160^ ^]^

**Figure 12:**
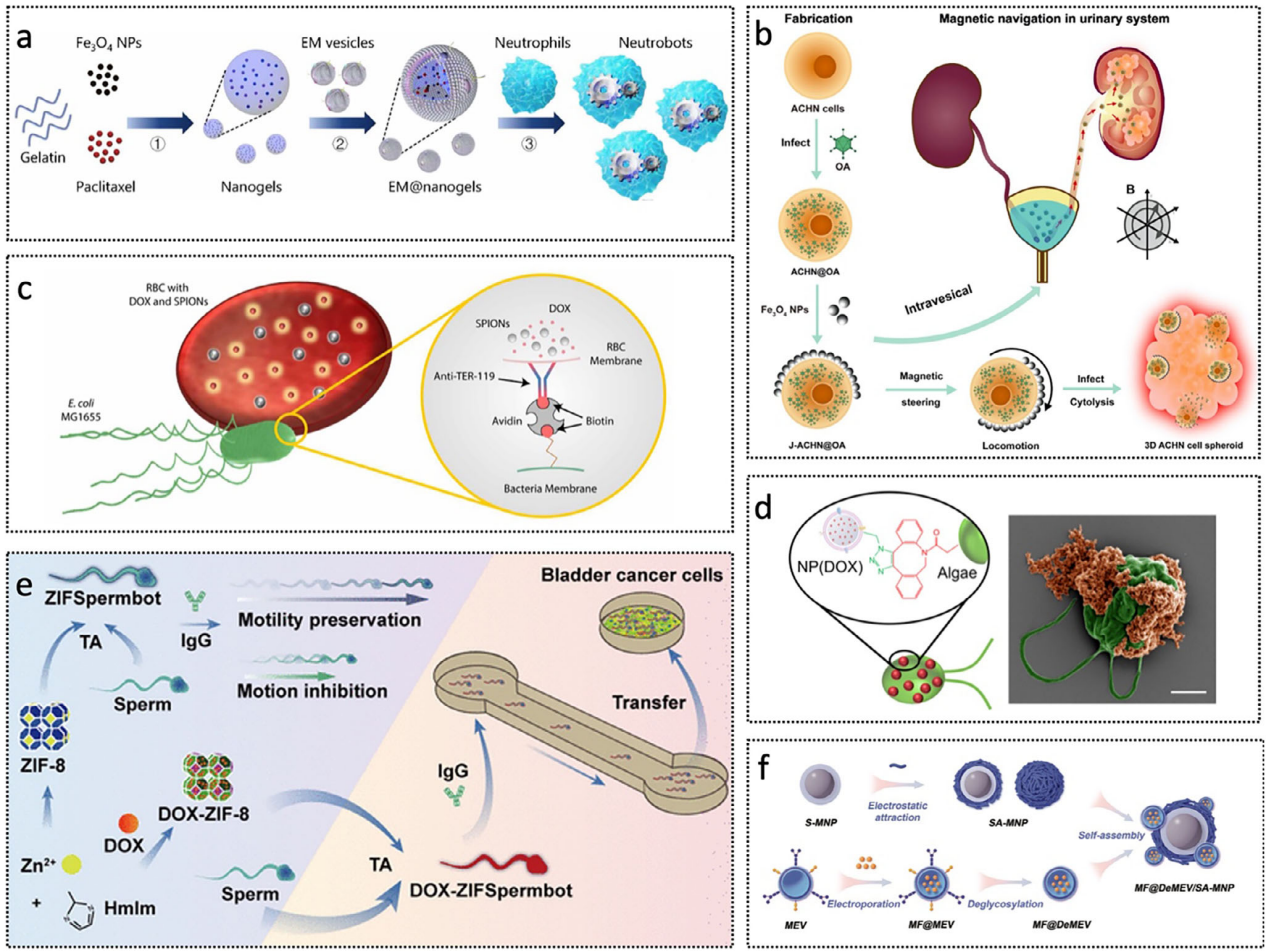
Biohybrid micro/nanorobots. a) Fabrication steps of the proposed neutrobots. Reproduced with permission from ref. [145] Copyright 2021, American Association for the Advancement of Science. b) OA‐loaded Janus tumor cell robot for active and specific tumor killing and magnetic navigation in the urinary system. Reproduced with permission from ref. [143] Copyright 2023, Elsevier. c) RBC microswimmer consists of RBCs loaded with drug molecules and SPIONs. Reproduced with permission from ref. [147] Copyright 2018, American Association for the Advancement of Science. d) Algae‐NP (DOX)‐robot, where NP (DOX) is covalently bound to the algae via click chemistry. Pseudo‐color scanning microscopy image of the robot with algae in green and NP (DOX) in orange. Scale bar, 2 µm. Reproduced with permission from ref. [154] Copyright 2024, American Association for the Advancement of Science. e) Manufacturing of ZIFSpermbots. Reproduced with permission from ref. [150] Copyright 2021, American Chemical Society. f) Construction process of the biohybrid nanorobotic platform (MF@DeMEV/SA‐MNP). Reproduced with permission from ref. [161] Copyright 2024, Wiley‐VCH.

Several representative biohybrid MNRs have been successfully developed, demonstrating significant potential across various biomedical applications. For instance, as illustrated in **Figure** **12**a, a neutrophil‐based microrobot was engineered by loading drug‐encapsulated magnetic nanogels cloaked with *Escherichia coli* membranes into natural neutrophils.^[^ ^145^ ^]^ The bacterial membrane disguise enhanced phagocytic uptake while preventing premature drug leakage within the neutrophils. Zhang et al. reported a macrophage–Janus magnesium microparticle hybrid robot capable of rapid locomotion and efficient endotoxin clearance in vivo.^[^ ^146^ ^]^ Similarly, Xie et al. developed a Janus tumor cell microrobot by asymmetrically coating tumor cells infected with oncolytic adenovirus with Fe_3_O_4_ nanoparticles (Figure 12b).^[^ ^143^ ^]^ This biohybrid system enables homotypic targeting, active oncolytic therapy, and magnetically guided navigation through complex tissue architectures. Alapan et al. reported a multifunctional biohybrid microswimmer (Figure 12c), which integrates engineered motile *E. coli* MG1655 with red blood cells loaded with doxorubicin (DOX) and superparamagnetic iron oxide nanoparticles (SPIONs), assembled through a biotin–avidin–biotin linkage.^[^ ^147^ ^]^ This system enabled active and targeted drug delivery under magnetic control.

Zhang et al. developed a biohybrid microrobot system, referred to as the algae–NP(DOX)–robot (Figure 12d), which integrates green microalgae with red blood cell membrane‐coated nanoparticles loaded with the chemotherapeutic agent DOX.^[^ ^154^ ^]^ This platform enabled autonomous propulsion within the lungs, enhancing the dispersion and release of drugs for antimetastatic lung cancer therapy. In addition, sperm cells have also inspired novel biohybrid microrobotic designs. For example, the ZIFSpermbot (Figure 12e) encapsulates sperm cells within a nanostructured matrix composed of metal–organic frameworks and zeolitic imidazolate framework‐8 nanoparticles, enabling active drug delivery and protection against anti‐sperm antibodies.^[^ ^150^ ^]^ Yan et al. developed a multifunctional biohybrid nanorobot platform (Figure 12f), which integrates magnetically responsive, tissue‐penetrating synthetic modules with glycosylated, drug‐loaded extracellular vesicles.^[^ ^161^ ^]^ This system addressesed delivery inefficiencies and significantly enhanced diabetic wound healing by improving cellular uptake and transdermal penetration, and facilitating multi‐step therapeutic interventions.

Although these biohybrid MNRs have demonstrated promising biocompatibility and functional performance in in vitro studies, their potential long‐term immunogenicity remains a critical concern. In the brain microenvironment, the long‐term stability of biological components such as neutrophils, bacteria, or sperm is uncertain, as they may undergo apoptosis, senescence, or rapid clearance by resident immune cells. Moreover, repeated or sustained exposure to foreign cellular elements could trigger adaptive immune responses, thereby limiting the therapeutic effect. To reduce these risks, several strategies have been proposed, including: i) surface coating with autologous cell membranes for camouflage; ii) genetic engineering to reduce pathogenicity while maintaining motility; iii) using transiently activated or self‐degrading biohybrid systems to minimize their long‐term presence in neural tissue. Future research should therefore focus on systematically evaluating the immune tolerance and biodistribution of biohybrid MNRs in brain‐like environments, which will be essential for ensuring their safe clinical translation.^[^ ^153^, ^162^ ^]^

Although significant advances have been made in the chemical, external‐field, and biohybrid propulsion of MNRs, their applicability in the complex brain environment and in treating brain disorders remains insufficiently understood. The effectiveness of different propulsion modes depends on brain tissue barriers, hemodynamics, and lesion characteristics such as depth and size. Magnetic propulsion, with its strong penetration and remote controllability, appears promising for deep‐seated tumors or vascular lesions. Chemical propulsion may support localized drug delivery or microenvironment‐responsive therapy but raises concerns about metabolic byproducts. Light‐driven propulsion offers high spatial resolution yet is restricted by the skull's optical window, making it more suitable for superficial or intraoperative applications. Ultrasound propulsion provides both penetration and relative safety, potentially benefiting stroke or diffuse lesions. Overall, future research should match propulsion strategies with disease type and lesion features, weighing penetration depth, navigation precision, biocompatibility, and clinical feasibility to clarify the suitability of single or multimodal approaches.

## 4 MNRs in Brain Disorder Treatment

MNRs have emerged as promising platforms for the diagnosis and treatment of brain disorders, as summarized in **Table** **2**. Recent advances demonstrate their potential in treating CVDs (e.g., thrombus removal and aneurysm repair), brain tumor therapy via BBB‐bypassing and BBB‐crossing strategies, and targeted interventions in neurodegenerative diseases, such as AD. These miniaturized robots show growing applicability in addressing other brain conditions, including neuroinflammation and traumatic brain injury.

**Table 2:** Summary of typical MNRs for combating brain disorders.

MNRs	Components	Brain Disorder	Size	External Source	Imaging	Stage	Refs.
UM‐NEs	Urease motor and Ag‐UK NPs	Thrombus	Microrobot	Chemical	Fluorescence/ Ultrasound	In vivo	Zheng et al.^[^ ^163^ ^]^
uTBMs	Microspheres capped with MNPs and cilia dual‐layer	Thrombus	Microrobot	Magnetic	Laser speckle imaging	In vitro/in vivo	Bao et al.^[^ ^164^ ^]^
tPA‐nbots	tPA‐anchored Fe_3_O_4_@mSiO_2_ nanorobots	Thrombus	Nanorobot	Magnetic	Fluoroscopy	In vitro/in vivo	Wang et al.^[^ ^165^ ^]^
HPB‐NRs	Magnetic beads elaborately grafted with heparinoid‐polymer brushes	Thrombus	Nanorobot	Magnetic	Laser speckle imaging	In vitro/in vivo	Yang et al.^[^ ^166^ ^]^
Magnetic colloidal collectives	225.9 ± 15 nm magnetic Fe_3_O_4_ colloids	Thrombus	Nanorobot	Magnetic	Ultrasound	In vitro/in vivo	Wu et al.^[^ ^167^ ^]^
MNS	Fe_3_O_4_‐DA nanoparticles with tPA	Thrombus	Nanorobot	Magnetic	Ultrasound	In vitro/in vivo	Wang et al.^[^ ^168^ ^]^
Self‐adhesive microgels	A pH‐responsive self‐healing hydrogel matrix, magnetic NPs, and imaging agents	Aneurysm	Microrobot	Magnetic	Fluoroscopy/Ultrasound	In vitro/ex vivo	Jin et al.^[^ ^169^ ^]^
SMP robot	Core: magnetic and RF dual‐responsive SMP; shell: chitosan and Fe_3_O_4_ NPs.	Aneurysm	Microrobot	Magnetic	N/A	In vitro	Liu et al.^[^ ^170^ ^]^
FTP nanorobots	Phase‐change material‐coated magnetite‐thrombin FTP nanorobots	Aneurysm	Nanorobot	Magnetic	Digital subtraction angiography	In vitro/in vivo	Wang et al.^[^ ^171^ ^]^
Microfiberbots	Thermal drawing magnetic soft composite into microfibers	Aneurysm	Microrobot	Magnetic	Fluoroscopy	In vitro/in vivo	Liu et al.^[^ ^172^ ^]^
BBHFs	The patient's own blood mixed with a small amount of magnetic particles	Glioblastoma	Microrobot	Magnetic	Fluoroscopy	In vitro/ex vivo	Wang et al.^[^ ^173^ ^]^
MCR‐based microrobot	THPP‐loaded DMs, TMZ‐loaded DMs and a magnetic continuum robot	Glioblastoma	Microrobot	Magnetic	Ultrasound	In vitro	Li et al.^[^ ^174^ ^]^
Nanorobot‐based marsupial robotic system	A marsupial robotic system constructed by integrating chemical/magnetic hybrid nanorobots with a miniature magnetic continuum robot	Glioblastoma	Nanorobot	Magnetic	Ultrasound	In vitro/ex vivo	Wu et al.^[^ ^175^ ^]^
wFIONs	Drug‐loaded micelles and water‐dispersible ferrimagnetic iron oxide nanocubes	Glioblastoma	Nanorobot	Magnetic	Fluorescence	In vivo	Kang et al.^[^ ^176^ ^]^
neutrobot	Phagocytosis of *Escherichia coli* membrane‐enveloped, drug‐loaded magnetic nanogels by natural neutrophils	Glioblastoma	Microrobot	Magnetic	Fluorescence/MRI	In vitro/in vivo	Zhang et al.^[^ ^145^ ^]^
PAMSe nanomotors	Loaded with brain endothelial cell targeting agent angiopep‐2 and anti‐tumor drug	Glioblastoma	Nanorobot	Chemical	Fluorescence	In vitro/in vivo	Chen et al.^[^ ^36^ ^]^
BMPNs	The membrane from the fusion of platelets and M1 macrophage membranes, resulting in fused membrane camouflaged PPy@Fe_3_O_4_ NPs to produce BMPNs	Glioblastoma	Nanorobot	Magnetic	Fluorescence	In vitro/in vivo	Song et al.^[^ ^177^ ^]^
MNP@BQR@ANG‐EXO‐siGPX4	A Fe_3_O_4_ core–mesoporous silica shell nanoparticle conjugated with CD63 antibody targets EVs from hMSCs. ANG is incorporated into the exosome membrane (ANG‐EXO), and GPX4 siRNA is loaded via electroporation	Glioblastoma	Nanorobot	Magnetic	Fluorescence	In vitro/in vivo	Li et al.^[^ ^178^ ^]^
MDNs	PEG‐IONPs and DOX	N/A	Nanorobot	Magnetic	Fluorescence/MRI	In vivo	Wang et al.^[^ ^179^ ^]^
BiVO_4_ micromotors	A straightforward surfactant‐free hydrothermal method based on Bi(NO_3_)_3_·5H_2_O and NaVO_3_ as Bi^3+^ and V^5+^ precursors	Neurodegenerative Diseases	Microrobot	Light	N/A	In vitro	Mayorga‐Burrezo et al.^[^ ^180^ ^]^
MOHR	A core–shell Si/SiO_2_/Ni/Au nanowire MIS junction	Alzheimer's disease	Microrobot	Magnetic/ Optoelectronic	Photoacoustic	In vitro/in vivo	Gao et al.^[^ ^181^ ^]^
PVA/BiFeO_3_/DO nanoparticles	Synthesis by co‐precipitation method using PVA, BiFeO_3_ and DO	Alzheimer's disease	Nanorobot	Magnetic/Electrical	N/A	N/A	Cesur et al.^[^ ^182^ ^]^
MSC‐IONPs	Iron oxide nanoparticle (IONP)‐incorporated human Wharton's jelly‐derived MSCs (MSC‐IONPs)	Alzheimer's disease	Nanorobot	Magnetic	Fluorescence	In vitro/in vivo	Jung et al.^[^ ^11^ ^]^
MM@MnO_2_‐Au‐mSiO_2_@Cur	Asymmetric structures with a mesoporous SiO_2_ head and multiple MnO_2_ tentacles	Traumatic brain injury	Nanorobot	Chemical	Fluorescence	In vitro/in vivo	Ye et al.^[^ ^183^ ^]^
JCNs	Constructed via plasma‐induced alloying technology and sputtering‐caused half‐coating strategy	Traumatic brain injury	Nanorobot	Chemical	Fluorescence	In vitro/in vivo	Wang et al.^[^ ^184^ ^]^

### 4.1 CVD Treatment

CVDs, including ischemic and hemorrhagic strokes, remain the leading causes of mortality and long‐term disability globally. These disorders primarily arise from thrombotic events or aneurysmal rupture. The following sections focus on recent advancements in MNR systems specifically designed to address these two pathological mechanisms.

#### 4.1.1 Thrombus

Thrombus formation, resulting from the abnormal aggregation of blood components within vessels, is a significant contributor to cardiovascular and cerebrovascular events. Its detachment can lead to embolization and downstream vascular occlusion, triggering conditions such as stroke or myocardial infarction.^[^ ^185^ ^]^

**Figure 13:**
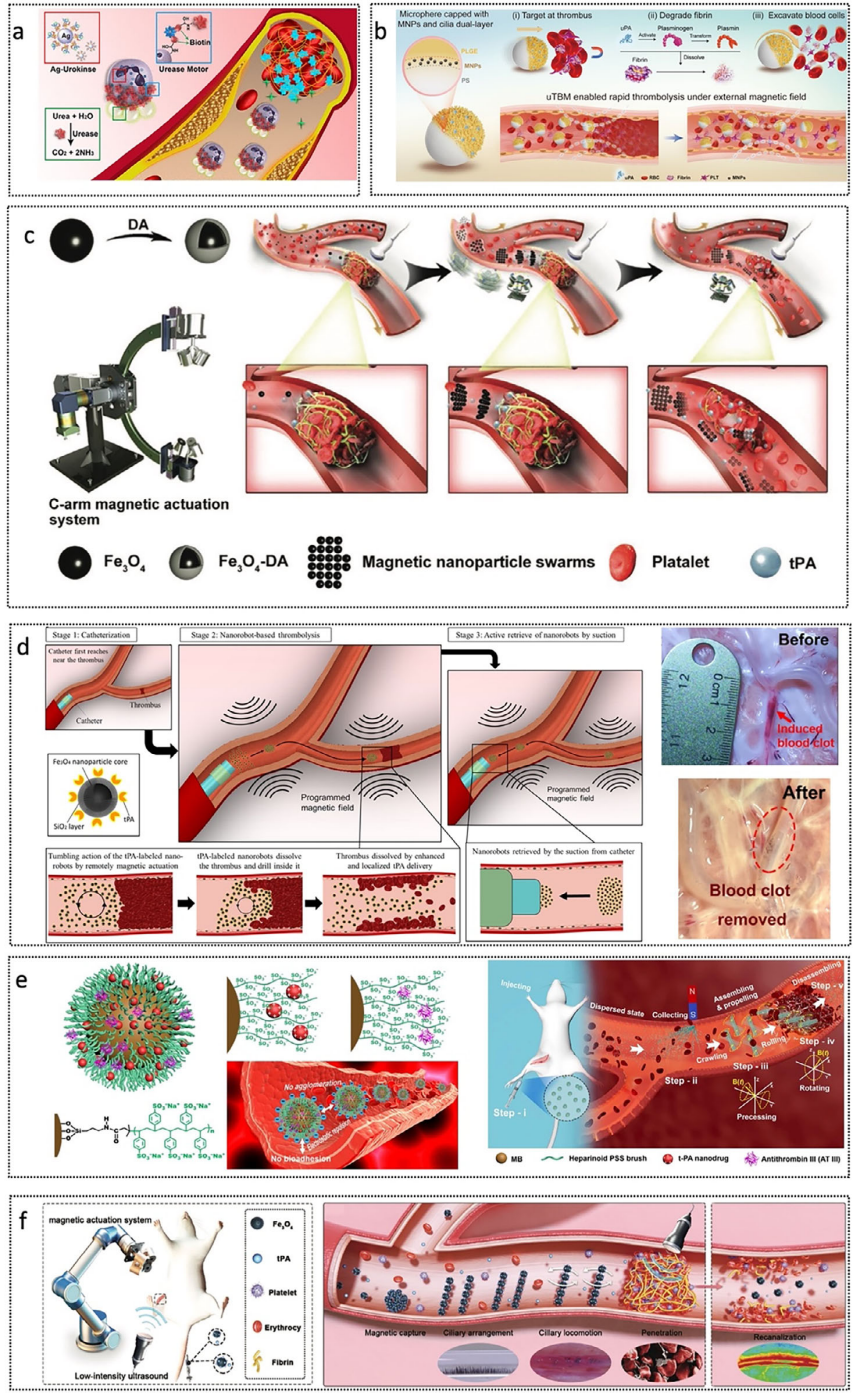
Micro/nanorobots for combating thrombus. a) Enzyme catalysis biomotor engineering of neutrophils. Reproduced with permission from ref. [163] Copyright 2022, American Chemical Society. b) uTBMs enabled rapid thrombolysis under the magnetic field manipulation. Reproduced with permission from ref. [164] Copyright 2025, Wiley‐VCH. c) Nanorobots actuated by a C‐shaped magnetic actuation system for enhanced thrombolysis in vivo. Reproduced with permission from ref. [168] Copyright 2021, Wiley‐VCH. d) tPA‐anchored nanorobots. Reproduced with permission from ref. [165] Copyright 2024, American Association for the Advancement of Science. d) Swarming HPB‐NRs based on MB@PSS NPs. Reproduced with permission from ref. [166] Copyright 2023, American Association for the Advancement of Science. e) Treatment using magnetically actuated colloids and low‐intensity ultrasound. Reproduced with permission from ref. [167] Copyright 2025, Wiley‐VCH.

Recent advances have enabled the development of sophisticated micro‐ and nanorobotic systems that couple biochemical catalysis with mechanical propulsion to enhance thrombus targeting and dissolution. As illustrated in **Figure** **13**a, a representative example is the urease micromotor‐driven neutrophil system (UM‐NEs), which is engineered to navigate and resolve thrombi actively.^[^ ^163^ ^]^ This design leverages neutrophils’ intrinsic chemotaxis toward inflammatory and thrombotic microenvironments. By catalyzing endogenous urea via urease, the system generates ammonia and carbon dioxide, producing thrust to propel neutrophils toward the lesion site. Upon arrival, inflammatory cues trigger the formation of neutrophil extracellular traps, facilitating the co‐release of urokinase‐conjugated silver nanoparticles to initiate thrombolysis and vascular recanalization in a synergistic manner. In vivo studies in murine models demonstrate rapid targeting, enhanced thrombolytic efficacy, prolonged circulation, reduced hemorrhagic risk, and favorable biocompatibility. However, the system's complex fabrication and the stringent requirements for enzyme immobilization present translational challenges.

Inspired by tunnel boring machines (TBMs), a next‐generation thrombus‐boring microrobot (uTBM) was introduced in 2025 to improve further thrombolysis in cardiovascular and cerebrovascular conditions (Figure 13b).^[^ ^164^ ^]^ Fabricated via a one‐step phase separation and interfacial self‐assembly process, uTBM features a cilia‐like surface architecture functionalized with urokinase (uPA) and magnetic nanoparticles (MNPs). Under remote magnetic actuation, the MNPs enable precise navigation and rotational drilling through fibrin networks, whereas the uPA‐functionalized cilia simultaneously facilitate biochemical degradation. Compared with conventional uPA‐based thrombolysis, this approach enhances thrombolytic efficiency by ≈8.5‐fold. uTBM exhibits rapid thrombus clearance, high drug utilization, mechanical potency, simplicity in design, and scalability. However, concerns over potential in vivo retention of MNPs warrant further investigation.

In 2021, Wang et al. proposed a general drug delivery strategy based on mass transportation theory, in which rotating magnetic fields drive magnetic nanoparticle swarms to achieve efficient and targeted transport and diffusion of the thrombolytic drug (tPA) under conditions of interrupted blood flow (Figure 13c).^[^ ^168^ ^]^ In parallel, they independently developed a C‐shaped magnetic actuation system, which was validated in a rabbit carotid artery thrombosis model, demonstrating its feasibility in rapidly establishing a blood flow channel and significantly enhancing thrombolytic efficiency. This approach requires no modification of the clinical drug dosage and holds strong potential for clinical translation. Further, Wang et al. developed a catheter‐assisted magnetic navigation platform integrating tPA‐anchored magnetic nanorobots (tPA‐nbots) and real‐time imaging via colloidal magnetic agent fluorescence imaging and steering to achieve precise intravascular navigation and thrombus‐resolving therapy (Figure 13d).^[^ ^165^ ^]^ This system enabled sub‐millimeter‐scale localization and controlled thrombolysis, combining mechanical agitation with enzymatic degradation at the target site. The enzymatic activity of tPA‐nbots is comparable to that of free tPA, enabling rapid thrombus lysis within 20–30 min in in vitro and in vivo settings. Notably, ≈80% of the nanorobots were retrievable post‐treatment, significantly reducing systemic drug exposure, mitigating off‐target effects, and preventing long‐term accumulation of non‐degradable materials.

Advancing the control of microrobot collective behavior, Yang et al. introduced a biocompatible swarm of magnetic nanorobots modified with heparin‐mimicking polymer brushes (HPBs, Figure 13e).^[^ ^166^ ^]^ Under alternating magnetic fields, these MNRs exhibited coordinated clustering, enabling mechanical thrombus disruption and localized drug release. HPB functionalization imparts superior hemocompatibility, including intrinsic anticoagulant properties, reversible dispersion, low hemolysis, and strong resistance to nonspecific adhesion. In vitro and in vivo experiments confirmed the rapid localization of thrombus, deep penetration, and complete lysis within ≈4 h. Post‐treatment, the MNRs degraded into nanoscale fragments that are immuno‐clearable, minimizing toxicity and eliminating organ damage.

Wu et al. proposed a magneto‐acoustic synergistic strategy, combining torque‐force vortex magnetic field‐driven cilia‐mimetic colloidal assemblies with low‐intensity ultrasound to further enhance thrombolytic performance (Figure 13f).^[^ ^167^ ^]^ This system mimics the helical motion of biological cilia, generating a vertical vortex that enhances fluidic agitation and drug diffusion, while improving the fidelity of ultrasound imaging. The addition of ultrasound facilitated clot softening and red blood cell deformation, promoting deeper drug penetration. Compared with tPA monotherapy, this hybrid approach achieved up to a 16‐fold increase in thrombolytic efficiency, accelerated recanalization, and significantly reduced drug dosage and off‐target effects.

Despite notable progress of MNRs in thrombus therapy, different designs show distinct strengths and limitations: UM‐NEs provide precise targeting but are difficult to fabricate, uTBMs improve thrombolytic efficiency with simpler structures yet raise concerns about particle retention, and HPB‐modified swarms combine hemocompatibility with biodegradability. Cross‐modal approaches such as magneto‐acoustic synergy further enhance efficacy and imaging integration. However, clinical translation is still limited by complex navigation systems, reliance on high‐resolution imaging, and insufficient long‐term safety data. Future work should emphasize multi‐physics actuation, biodegradable materials, and integration with clinical imaging, while addressing unmet needs in navigation simplification, safety validation, and cost control.

#### 4.1.2 Aneurysm

Cerebral aneurysms, characterized by localized saccular dilations arising from weakened arterial walls, pose significant clinical risks because their rupture can cause lethal SAH. Advances in minimally invasive interventions have increasingly integrated emerging micro‐ and nanorobotic platforms and smart materials into precise aneurysm embolization therapies, demonstrating significant technological advantages and clinical potential.

**Figure 14:**
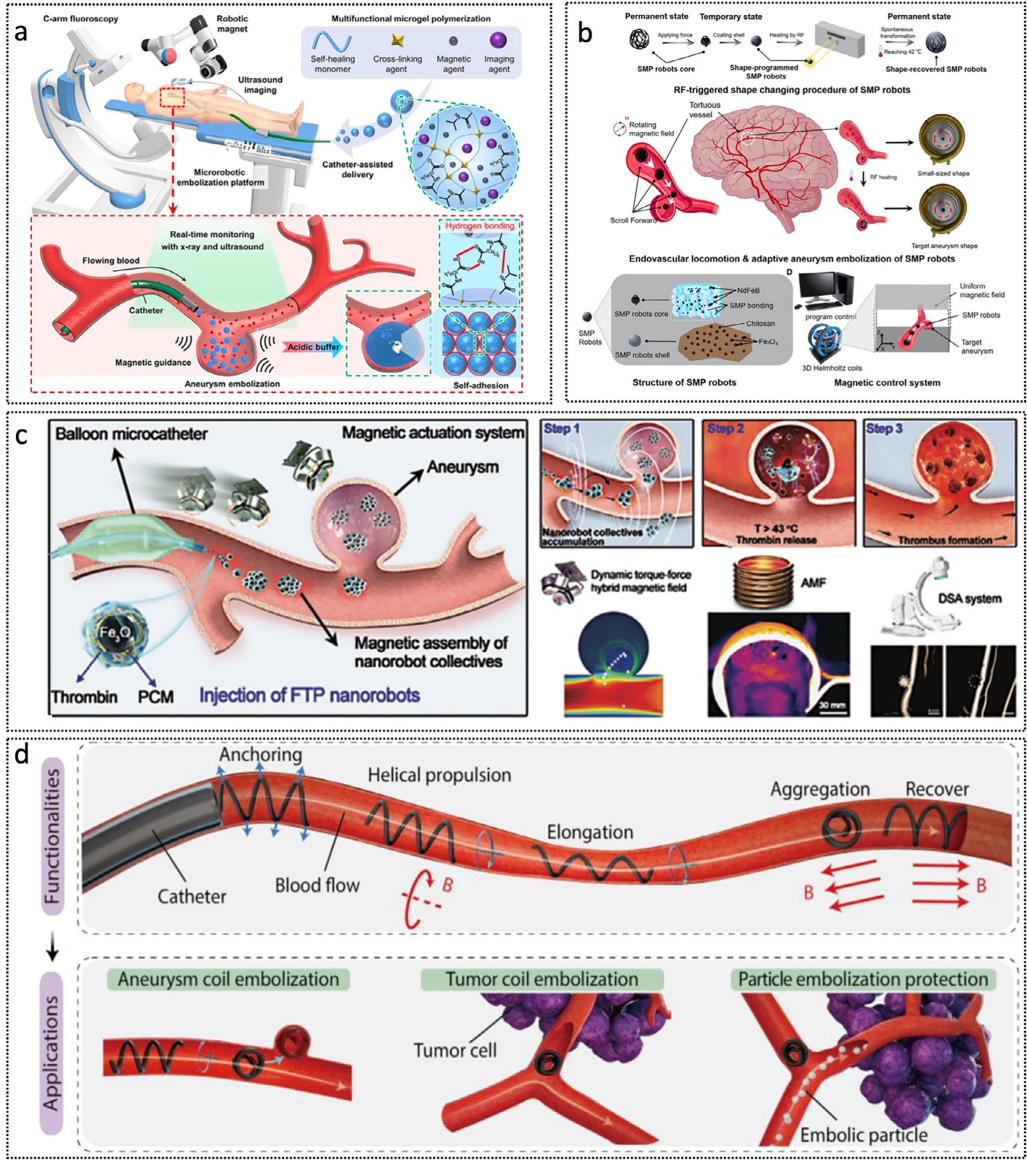
Micro/nanorobots for combating aneurysms. a) Swarming self‐adhesive microgels. Reproduced with permission from ref. [169] Copyright 2023, American Association for the Advancement of Science. b) Dual‐responsive shape‐programmable robots for adaptive aneurysm embolization. Reproduced with permission from ref. [170] Copyright 2023, ScienceDirect. c) Thermal‐responsive FTP nanorobot collectives to induce rapid embolization in vivo. Reproduced with permission from ref. [171] Copyright 2024, Wiley‐VCH. (d) Magnetic soft microfiberbots for robotic embolization. Reproduced with permission from ref. [172] Copyright 2024, American Association for the Advancement of Science.

Jin et al. presented an advanced micro‐robotic platform (**Figure** **14**a) specifically designed for targeted aneurysm embolization, combining catheter‐assisted delivery with magnetic swarm control technology.^[^ ^169^ ^]^ This platform utilizes pH‐responsive, self‐healing hydrogel‐based microgels embedded with MNPs and imaging agents, enabling rapid and stable self‐adhesion under mildly acidic conditions. Guided by real‐time ultrasound and fluoroscopic imaging, these microgels can precisely aggregate within aneurysm sacs under an external magnetic field, achieving targeted embolization. Their triggered self‐adhesive mechanism effectively overcomes common drawbacks associated with traditional embolic agents, such as leakage, fragility, and poor controllability. The experimental results demonstrated excellent embolization performance under simulated physiological flow conditions, along with multiple advantages including controllable deployment, precise targeting, good biocompatibility and hemocompatibility, and long‐term stability.

Building upon this, researchers further developed a novel aneurysm treatment strategy on the basis of shape‐programmable microrobots constructed from magnetic‐ and radiofrequency (RF)‐responsive shape memory polymers (SMPs, Figure 14b).^[^ ^170^ ^]^ This SMP‐based microrobot addresses key challenges encountered by conventional metallic coil embolization, particularly navigation through tortuous vasculature and adaptability to aneurysm conformation. Under programmable magnetic field guidance, the robot autonomously navigates narrow blood vessels and undergoes shape reconfiguration triggered by RF stimulation, adapting precisely to the geometry of aneurysms. Exhibiting superior mechanical properties with tensile and bending moduli of 276 and 321 MPa, respectively, the robot demonstrated high durability and a low risk of fracture. It achieved rapid and precise locomotion in both 2D and 3D spaces, possesses a curing rate exceeding 93%, and can expand its diameter by over twofold upon RF activation. These attributes highlight its significant potential for precise localization, efficient filling, and personalized deployment, underscoring its clinical value in enhancing the efficacy of aneurysm embolization.

Addressing key challenges in treating intracranial aneurysms, researchers proposed a stent‐ and microcatheter‐free intravascular intervention strategy using untethered magnetically actuated nanorobots (Fe_3_O_4_‐Th@PCM) for targeted thrombin delivery (Figure 14c).^[^ ^171^ ^]^ These nanorobots, coated with phase‐change materials, achieved precise navigation within the vascular system and targeted aggregation within aneurysm sacs under synergistic guidance by dynamic magnetic fields and real‐time ultrasound imaging. Upon magnetothermal stimulation, the nanorobots release thrombin, initiating rapid and stable embolization of the aneurysm. Imaging and histological analyses further validated the efficacy of this strategy. Compared with traditional stent‐assisted embolization, this approach avoids long‐term complications associated with implanted foreign materials, reduces dependence on antiplatelet medications, enhances therapeutic delivery efficiency, and mitigates risks of drug leakage.

Notably, traditional embolization techniques often face limitations in navigating complex submillimeter vascular networks due to their limited maneuverability, and manual catheter navigation carries significant risks of radiation exposure. Magnetic soft microfiber robotic technologies have emerged to overcome these challenges (Figure 14d).^[^ ^172^ ^]^ Driven by a six‐degree‐of‐freedom robotic arm and propelled using helical magnetic propulsion, these robots offer exceptional maneuverability and on‐demand aggregation capability, effectively interrupting blood flow at targeted pathological areas. This system has been validated preliminarily in neurovascular models and in vivo rabbit studies, demonstrating its potential for robot‐assisted embolization therapy in cerebral aneurysms and brain tumors.

### 4.2 Brain Tumor Treatment

Brain tumors remain among the most challenging malignancies to treat, due to the restrictive nature of the BBB, which significantly limits the delivery of therapeutic agents. Recent advances in MNR systems offer promising strategies to overcome this obstacle. These systems can either bypass the BBB via localized delivery approaches or actively cross it through controlled navigation and penetration mechanisms. Such capabilities position MNRs as a transformative platform for targeted and minimally invasive brain tumor therapy.

#### 4.2.1 Bypassing BBB

In recent years, a diverse array of innovative, minimally invasive, and targeted therapeutic strategies has emerged for the treatment of GBM, an aggressive and highly lethal primary brain tumor. These strategies are designed to overcome the inherent limitations of conventional treatments, including surgical resection, radiotherapy, and systemic chemotherapy. A central focus has been the development of techniques to bypass the BBB, thereby enabling more effective intracranial drug delivery.

**Figure 15:**
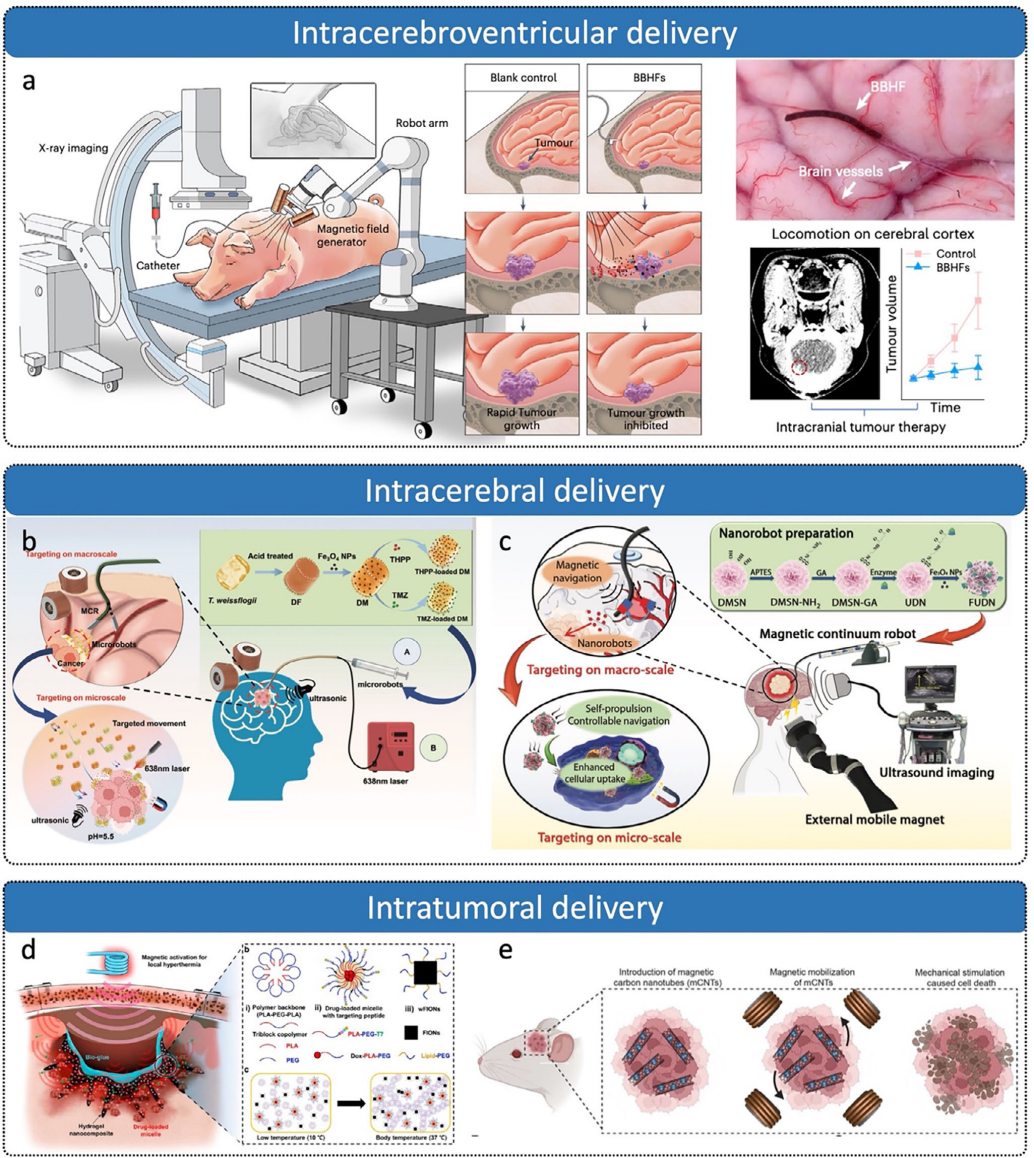
Micro/nanorobots bypass BBB to combat brain tumors. a) Magnetically driven BBHFs for intracranial tumor therapy. Reproduced with permission from ref. [173] Copyright 2025, Springer Nature. b) MCR‐based microrobot cross‐scale delivery method. Reproduced with permission from ref. [174] Copyright 2024, Wiley‐VCH. c) Marsupial robotic system for intracranial cross‐scale drug delivery. Reproduced with permission from ref. [175] Copyright 2024, Wiley‐VCH. d) Penetrative drug delivery to the deep brain tumor. Reproduced with permission from ref. [176] Copyright 2023, American Chemical Society. e) Nanosurgery using the mCNTs. Reproduced with permission from ref. [186] Copyright 2023, American Association for the Advancement of Science.

Wang et al. introduced a biodegradable, magnetically responsive biohybrid hydrogel fiber (BBHF) designed for the minimally invasive and targeted treatment of intracranial tumors (**Figure** **15**a).^[^ ^173^ ^]^ This system is rapidly fabricated (∼15 min) by combining autologous blood with magnetic particles and designed for precise navigation using external magnetic fields. BBHF exhibits exceptional immune evasion, X‐ray fluoroscopic traceability, and multimodal locomotion capabilities (e.g., swinging, crawling, and rolling), facilitating accurate navigation and payload delivery within the complex brain microenvironment. Upon tumor‐site localization, chemotherapeutic agents such as DOX can be released on demand via high‐frequency magnetic triggering. In vivo studies in miniature pigs confirmed excellent biocompatibility and therapeutic efficacy, with no long‐term toxicity observed in major organs. Notably, BBHF generated from allogeneic blood induced significant inflammatory responses, underscoring the critical importance of autologous materials in personalized neurotherapeutics. Collectively, this platform integrates biodegradability, immune invisibility, and precise controllability, offering a promising and safe strategy for personalized intracranial drug delivery.

In the realm of cross‐scale delivery, researchers have proposed a synergistic strategy combining a magnetically controlled continuum robot (MCR) with microrobots for GBM treatment (Figure 15b).^[^ ^174^ ^]^ Diatom‐inspired microrobots are loaded with temozolomide (TMZ) and the photosensitizer THPP via vacuum adsorption. These microrobots demonstrated upstream locomotion and collective behavior in physiological fluids. The drug release is pH‐sensitive and temporally controllable, and therapeutic activation is achieved through ultrasound‐triggered photoactivation. MCR enables the precise deployment of microrobots and optical fibers deep into brain tissue, overcoming the dual challenges of BBB impermeability and limited light penetration. In vitro assays revealed significant enhancements in GBM cell‐killing efficiency, coupled with reduced drug dosage and laser power requirements, highlighting the system's potent synergistic effects. Efficacy was further validated in 3D‐printed physiological models. However, clinical translation requires refinement of MCR control precision and operational robustness.

A complementary cross‐scale strategy integrates MCR navigation with magnetochemically responsive nanorobots encapsulated in a “mother‐to‐child” delivery system (Figure 15c).^[^ ^175^ ^]^ The MCR performs macroscopic intracranial navigation and releases nanorobots at the tumor site, enabling microscale, high‐precision drug delivery. This hierarchical paradigm addresses key challenges, including impaired nanorobot motility, BBB obstruction, and nonspecific drug diffusion. Validation in patient‐derived GBM cell models and ex vivo porcine brain tissues demonstrated enhanced targeting precision and therapeutic efficacy. However, real‐time monitoring, stable control mechanisms, and long‐term biosafety remain critical hurdles for clinical application.

For postoperative management, an injectable, thermosensitive hydrogel nanocomposite has been developed to eradicate residual tumor cells that evade conventional treatment (Figure 15d).^[^ ^176^ ^]^ This system consists of drug‐loaded micelles and water‐dispersible ferrimagnetic iron oxide nanocubes, which undergo a sol–gel transition at physiological temperatures to form a soft drug reservoir within the resection cavity. The hydrogel enables pH‐responsive drug release tailored to TME, and the alternating magnetic fields induced localized hyperthermia, enhancing drug diffusion and tissue penetration. In orthotopic mouse models, this approach significantly inhibited tumor recurrence and extended survival, demonstrating high potential for localized, postoperative therapy.

A mechanical nanosurgical approach employing rotating magnetic carbon nanotubes (mCNTs) has recently garnered increasing attention (Figure 15e).^[^ ^186^ ^]^ Upon cellular internalization into GBM cells, the mCNTs can be actuated by external magnetic fields to disrupt intracellular structures, thereby inducing cytotoxic effects physically. Further functionalization of mCNTs with anti‐CD44 antibodies enhances their specificity toward tumor cells and improves their retention within the targeted regions. This strategy has demonstrated promising efficacy in chemoresistant GBM models, offering a novel physical modality that complements biochemical therapies.

In summary, these emerging cross‐scale delivery systems and physical intervention strategies represent significant advances in the precision treatment of GBM. By bypassing the BBB, enhancing therapeutic specificity, and enabling personalized interventions, such technologies are accelerating the clinical translation of minimally invasive neurotherapeutics. Future research efforts should focus on enhancing system stability, refining image‐guided navigation, improving immunocompatibility, and validating efficacy in large animal models to fully translate these advanced approaches into clinical practice fully.

#### 4.2.2 Crossing BBB

Another strategy for treating brain tumors by using MNR systems involves crossing the BBB. Recent studies have significantly enhanced the efficiency of targeted drug delivery by leveraging carriers such as cells, exosomes, and nanoparticles with intrinsic BBB‐penetrating capabilities.

**Figure 16:**
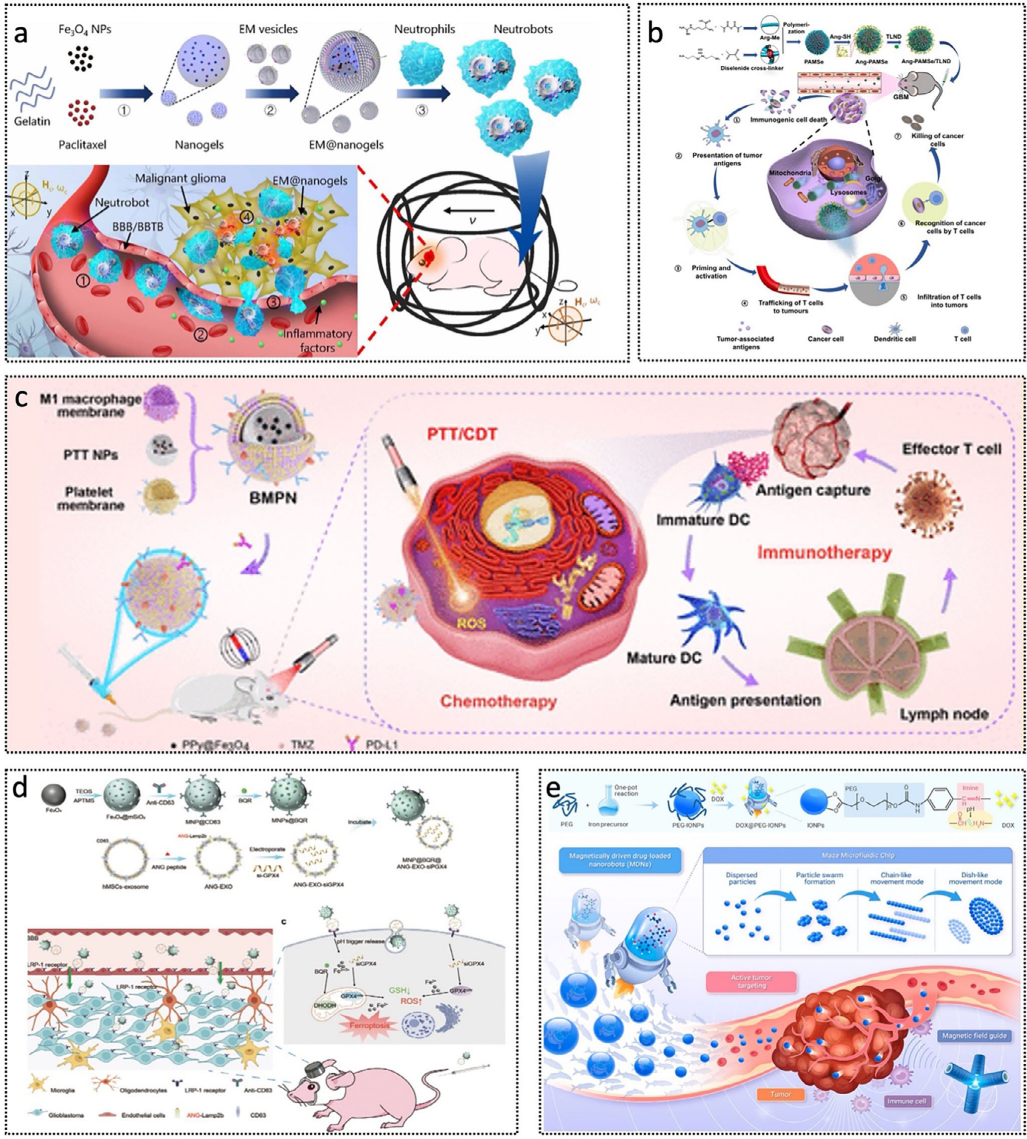
Micro/nanorobots cross BBB to combat brain tumors. a) Dual‐responsive biohybrid neutrobots. Reproduced with permission from ref. [145] Copyright 2021, American Association for the Advancement of Science. b) Nitric‐oxide‐driven chemotactic nanomotor. Reproduced with permission from ref. [36] Copyright 2023, Springer Nature. c) Hybrid membrane biomimetic photothermal nanorobots. Reproduced with permission from ref. [177] Copyright 2025, American Chemical Society. d) Engineered exosome‐conjugated MNPs for GBM therapy. Reproduced with permission from ref. [178] Copyright 2022, Wiley‐VCH. e) Magnetically driven bionic nanorobots. Reproduced with permission from ref. [179] Copyright 2025, ScienceDirect.

In 2021, researchers developed a dual‐responsive neutrophil‐based microrobotic system, termed the “neutrobot” (**Figure** **16**a), which integrates the natural chemotaxis and immune evasion capabilities of neutrophils with magnetic actuation.^[^ ^145^ ^]^ This system was constructed by enabling neutrophils to phagocytose drug‐loaded magnetic nanogels cloaked with E. coli membranes, significantly enhancing drug encapsulation efficiency while minimizing premature drug leakage. Under the influence of an RMF, the neutrobots exhibited controllable locomotion and autonomously crossed the BBB, guided by inflammatory signals, to achieve precise drug delivery to GBM sites.

Building on this concept, a chemotaxis‐responsive nanomotor system (Ang‐PAMSe/TLND, Figure 16b) was developed, integrating the brain endothelium‐targeting peptide Angiopep‐2 and the mitochondrion‐targeting agent clindamycin (TLND).^[^ ^36^ ^]^ This nanomotor responds to GBM‐specific chemotactic cues, such as reactive oxygen species (ROS) and inducible nitric oxide synthase, to achieve efficient delivery and immune activation. Through in situ covalent modification, the nanomotors exhibit enhanced motility, sustained nitric oxide (NO) release, and excellent BBB penetration. Once across the BBB, they further target tumor mitochondria, inducing immunogenic cell death, activating dendritic cells, promoting T cell infiltration and macrophage polarization, and downregulating immunosuppressive markers, ultimately establishing immune memory to prevent tumor recurrence.

Song et al. proposed a multifunctional biomimetic magnetic nanorobot (BMPN) for GBM therapy (Figure 16c).^[^ ^177^ ^]^ By fusing platelet and M1 macrophage membranes, BMPN demonstrated enhanced BBB permeability and tumor‐targeting capability. The nanorobot encapsulated a polypyrrole/Fe_3_O_4_ nanocomposite for NIR‐triggered photothermal therapy (PTT) and CDT, and co‐delivered TMZ and anti–PD‐L1 antibodies, enabling a combination of chemotherapy and immune checkpoint blockade. The BMPN system effectively modulated tumor immunity through PTT/CDT‐induced immune activation, transforming immunologically “cold” tumors into “hot” ones and enhancing the efficacy of immunotherapy. The platform exhibited excellent biocompatibility, high photothermal conversion efficiency, and superior photoacoustic imaging performance and demonstrated significant tumor suppression under magnetic guidance in GBM models.

Li et al. developed an engineered exosome–magnetic nanoparticle hybrid platform for GBM treatment (Figure 16d) to enhance ferroptosis induction.^[^ ^178^ ^]^ The system combines localized magnetic targeting, Angiopep‐2‐modified exosome‐mediated BBB penetration, and a synergistic ferroptosis mechanism via Fe^2^⁺ release, dihydroorotate dehydrogenase inhibition, and glutathione peroxidase 4 (GPX4) silencing. This platform offers efficient BBB traversal, low‐density lipoprotein receptor‐related protein 1‐mediated GBM cell targeting, and excellent biocompatibility and supports external control via wearable magnetically actuated devices.

Additionally, Wang et al. designed a magnetically driven biomimetic drug‐loaded nanorobot (MDN) on the basis of ultrasmall iron oxide nanoparticles for precise tumor targeting and chemotherapy delivery (Figure 16e).^[^ ^179^ ^]^ Navigated by a custom‐designed 3D magnetic manipulation platform, the MDNs mimic coordinated fish‐like swarming behavior to achieve efficient navigation through complex physiological environments. The DOX‐loaded MDNs achieved a over tenfold increase in tumor accumulation, significantly reduced systemic toxicity, enhanced immune activation, and prolonged survival in tumor‐bearing mice. This strategy, combining magnetic resonance imaging (MRI)‐guided navigation and targeted delivery, demonstrated substantial potential for precision oncology applications.

Overall, MNRs hold great promise for brain tumor therapy by overcoming the BBB and enabling precise drug delivery, with each design offering distinct strengths. Cross‐scale systems that bypass the BBB provide spatial precision and personalized treatment but face challenges in control accuracy and long‐term safety, whereas BBB‐crossing platforms achieve superior efficacy through immune evasion and microenvironmental responsiveness, yet remain limited by scalability and immune evaluation. Future efforts should focus on multimodal actuation integration, personalized material design, and imaging‐guided monitoring, while addressing stability, controllability, and translational validation to advance clinical application.

### 4.3 Neurodegenerative Disease Treatment

Neurodegenerative diseases such as AD remain challenging to treat effectively at present due to their complex pathological mechanisms and difficulties associated with drug delivery to the brain. In recent years, micro‐ and nanorobotic systems have emerged as promising therapeutic tools, offering precise navigation and targeted treatment capabilities that hold great potential for advancing the treatment of these diseases.

**Figure 17:**
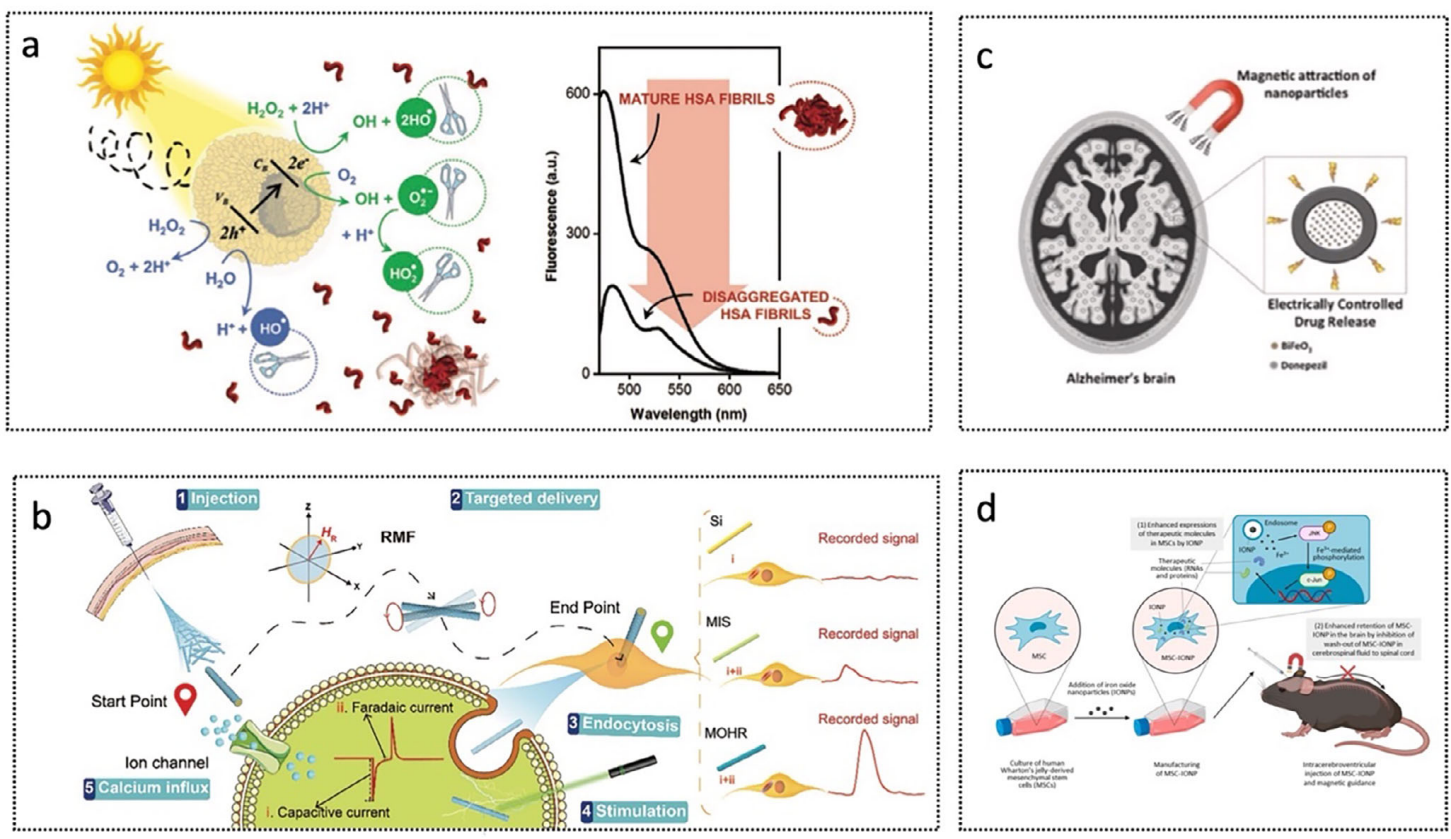
Micro/nanorobots for combating neurodegenerative diseases. a) Light‐driven micromotors to dissociate protein aggregates causing the NDs. Reproduced with permission from ref. [180] Copyright 2022, Wiley‐VCH. b) Targeted optically non‐genetic neuromodulation by the MOHR. Reproduced with permission from ref. [181] Copyright 2024, Wiley‐VCH. c) Donepezil and BiFeO_3_ magnetic nanoparticle‐loaded PVA microbubbles/nanoparticles. Reproduced with permission from ref. [182] Copyright 2022, Elsevier. d) Iron oxide nanoparticle‐incorporated MSCs. Reproduced with permission from ref. [11] Copyright 2023, American Chemical Society.

Microrobots have become prominent candidates in addressing AD, representing a typical example of treatments for neurodegenerative disorders. Mayorga‐Burrezo et al. developed an autonomous, single‐component, light‐driven micromotor on the basis of concave BiVO_4_ microspheres, specifically designed to disassemble mature protein fibrils associated with neurodegenerative diseases (**Figure** **17**a).^[^ ^180^ ^]^ The micromotor efficiently achieved fibril dissociation through its intrinsic photocatalytic generation of ROS. Furthermore, its distinctive helical trajectory promoted uniform distribution of ROS, thereby enhancing therapeutic efficacy. This study highlighted the substantial potential of micromotors as targeted, minimally invasive therapeutic strategies for currently incurable neurodegenerative disorders. The approach presented could be extended to other ROS‐based photodynamic therapies, such as treatments for lung or skin cancers.

Gao et al. proposed an innovative magnetically controlled optoelectronic hybrid microrobot (MOHR), designed for precise, non‐invasive, and non‐genetic optical neuromodulation at the single‐cell level (Figure 17b).^[^ ^181^ ^]^ The MOHR system integrates a metal‐insulator‐semiconductor junction with embedded magnetic nickel components, demonstrating outstanding controllability, efficient optoelectronic responses, and versatile magnetic manipulation modes (including axial rolling, radial rolling, and wobbling) suitable for various biological environments. This microrobot exhibited robust capacitive currents, excellent biocompatibility, and low cytotoxicity, enabling effective cellular internalization and precise modulation of neuronal excitability upon visible‐light stimulation. Remarkably, the system successfully restored neuronal activity impaired by β‐amyloid peptides, underscoring its potential for treating neurodegenerative diseases such as AD.

The development of nanorobots has provided novel strategies for treating neurodegenerative disorders. In 2022, Cesur developed polyvinyl alcohol‐based nanoparticles loaded with magnetic bismuth ferrite (BiFeO_3_) nanoparticles and donepezil hydrochloride (DO), synthesized via a microfluidic T‐junction device utilizing explosive microbubbles (Figure 17c).^[^ ^182^ ^]^ This platform was specifically designed for controlled drug delivery in AD therapy. The system demonstrated excellent biocompatibility, with no cytotoxic effects on healthy cells. A notable advantage of this system is its electrically tunable drug release capability: electrical stimulation effectively modulates DO release, achieving up to 68.9% cumulative release at −1.0 V after 15 cycles, following a non‐Fickian diffusion mechanism.

Cell‐based therapeutic approaches for neurodegenerative diseases have also emerged as promising alternatives. Jung et al. enhanced the therapeutic efficacy of mesenchymal stem cells (MSCs) derived from human Wharton's jelly (MSC‐IONP) in AD models by incorporating iron oxide nanoparticles (IONPs, Figure 17d).^[^ ^11^ ^]^ This strategy overcame traditional MSC therapy limitations such as poor brain retention and suboptimal therapeutic outcomes. Under magnetic guidance, MSC‐IONP exhibited significantly improved brain retention, increased expression of therapeutic molecules, marked reduction in amyloid‐β plaques, and enhanced cognitive recovery in a 5xFAD mouse model compared with conventional MSCs. The use of allogeneic MSCs derived from Wharton's jelly addressed potential limitations associated with autologous MSCs obtained from elderly patients with AD.

### 4.4 Other Brain Disease Treatment

In addition to using MNRs to treat the abovementioned brain diseases, the treatment of other brain diseases, such as neuroinflammation and TBI, can be optimized using the developed innovative MNR systems.

**Figure 18:**
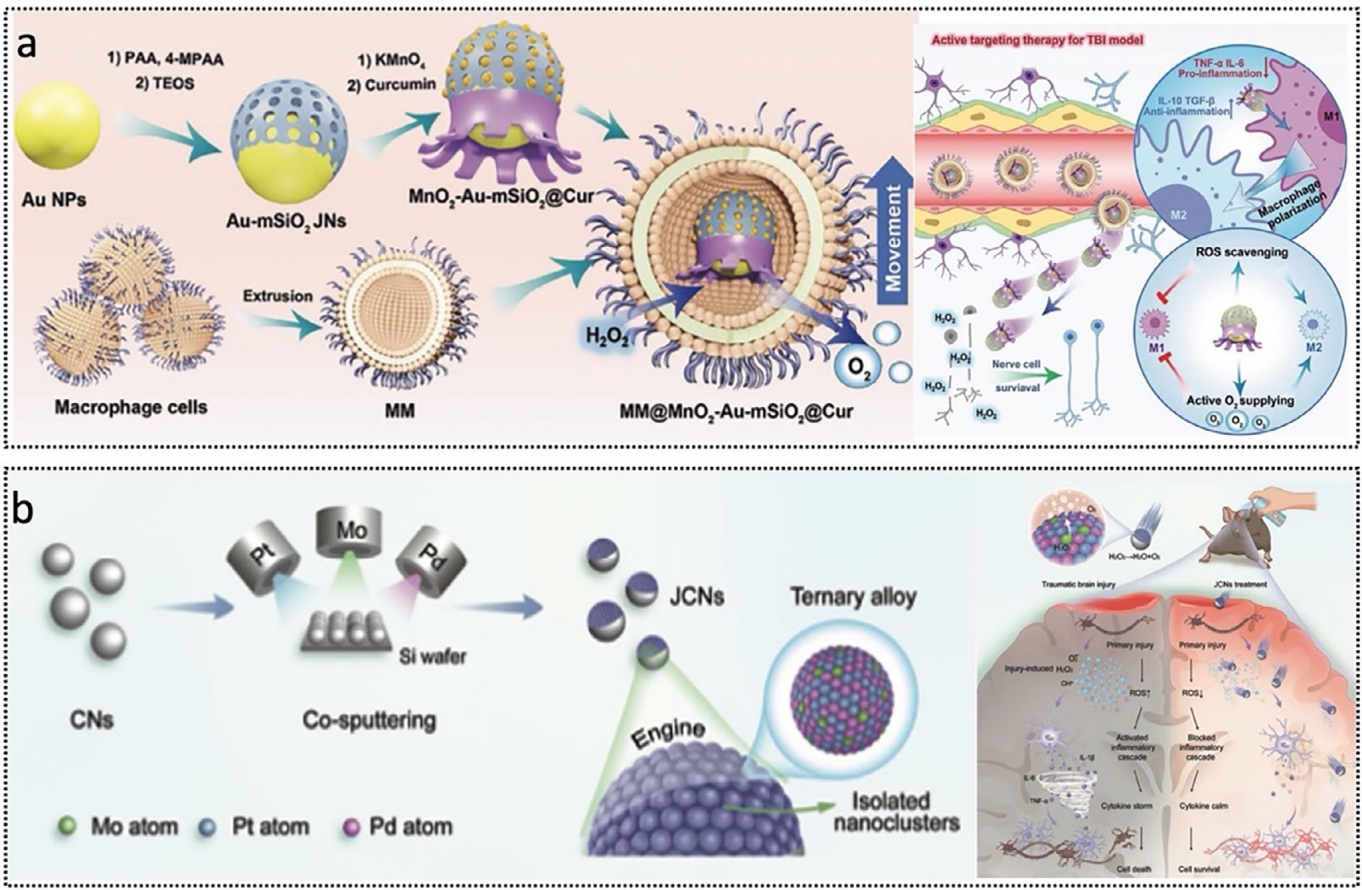
Micro/nanorobots for combating other brain diseases. a) Self‐propelled asymmetric nanomotors. Reproduced with permission from ref. [183] Copyright 2024, Wiley‐VCH. b) Self‐propelled nanomotor with an alloyed engine for treatment of TBI. Reproduced with permission from ref. [184] Copyright 2022, Wiley‐VCH.

Ye et al. reported a biomimetic self‐propelled nanomotor designed for precise anti‐inflammatory therapy in CNS inflammatory diseases, as illustrated in **Figure** **18**a.^[^ ^183^ ^]^ Inspired by the asymmetric structure of an octopus, this nanomotor comprises a mesoporous silica (SiO_2_) head and several manganese dioxide (MnO_2_) tentacles. Coated with macrophage‐derived membranes, these nanomotors effectively cross the BBB and actively target inflammatory sites deep within the brain tissue. The MnO_2_ tentacles catalyze the decomposition of hydrogen peroxide (H_2_O_2_), generating oxygen bubbles that not only relieve inflammation and scavenge ROS but also serve as a driving force, thereby enhancing tissue penetration. Concurrently, the curcumin‐loaded silica head modulates macrophage polarization, shifting macrophages from a pro‐inflammatory M1 phenotype toward an anti‐inflammatory M2 phenotype. Extensive in vitro, organoid, and animal studies have demonstrated the efficacy of nanomotors in alleviating inflammation, restoring neurological function, and improving cognitive outcomes in TBI models.

Furthermore, a novel self‐propelled Janus catalytic nanomotor (JCN) platform has been developed for rapid emergency pretreatment of severe traumatic brain injury (sTBI), as illustrated in Figure 18b.^[^ ^184^ ^]^ This platform achieved superior catalytic activity via plasma‐induced alloying combined with a sputtering‐based semi‐coating strategy. By utilizing endogenous hydrogen peroxide generated during oxidative stress as fuel, these nanomotors efficiently penetrate brain tissue, swiftly scavenging ROS and nitrogen species and significantly suppressing inflammatory cascades. The experimental results indicated that JCNs remarkably reduced behavioral deficits and mortality rates in mouse models of sTBI, highlighting their potential for immediate therapeutic intervention without reliance on external equipment.

## 5 MNR Imaging and Tracking

MNRs offer great potential for targeted brain disorder therapies owing to their precise controllability and delivery capabilities. However, effective imaging and tracking are crucial to realize their diagnostic and therapeutic functions fully. Given the brain's complex anatomy, dynamic environment, and sensitivity to operational errors, imaging systems must combine high spatiotemporal resolution with compatibility with MNRs’ actuation and navigation. Various imaging modalities have been explored, including single‐modality techniques such as fluorescence imaging,^[^ ^135^, ^145^ ^]^ MRI,^[^ ^187^ ^]^ positron emission tomography (PET),^[^ ^188^ ^]^ X‐ray,^[^ ^189^ ^]^ computed tomography (CT), fluoroscopy,^[^ ^165^, ^169^, ^172^, ^190^ ^]^ and ultrasound (US),^[^ ^191^ ^]^ as well as multimodal systems like photoacoustic imaging (PAI) and MR‐guided focused ultrasound (MRgFUS).^[^ ^192^, ^193^, ^194^ ^]^ Each imaging technology brings unique strengths and inherent limitations, including spatiotemporal resolution.

**Figure 19:**
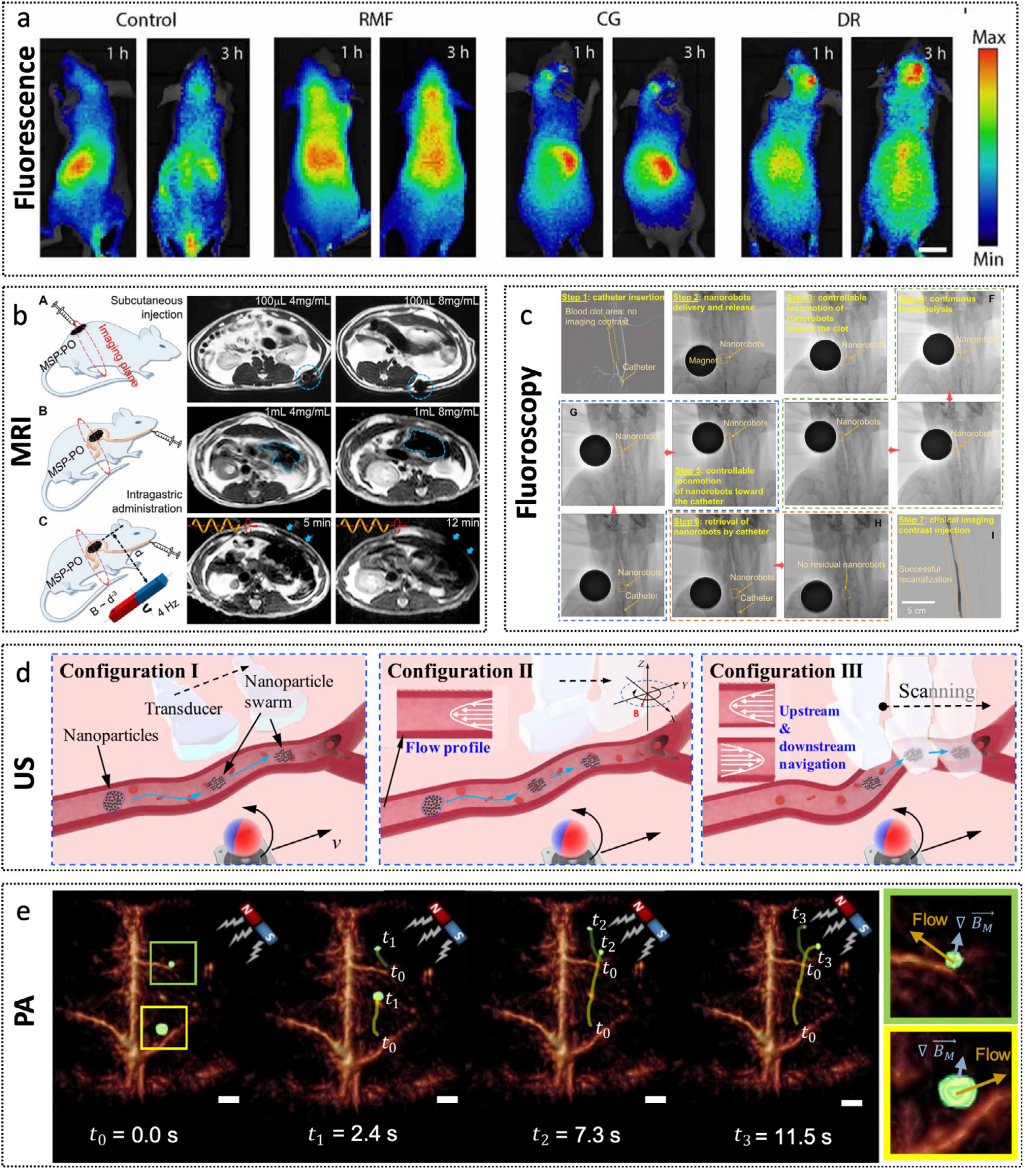
Micro/nanorobot imaging and tracking. a) Fluorescence images of mice treated with the neutrobots. Reproduced with permission from ref. [145] Copyright 2021, American Association for the Advancement of Science. b) T_2_‐weighted cross‐sectional MRI of microrobots inside rats. Reproduced with permission from ref. [187] Copyright 2017, American Association for the Advancement of Science. c) Fluoroscopy‐guided thrombolysis using tPA‐nbots. Reproduced with permission from ref. [165] Copyright 2024, American Association for the Advancement of Science. d) US Doppler imaging–guided swarm navigation in blood vessels. Reproduced with permission from ref. [198] Copyright 2021, American Association for the Advancement of Science. e) PAI of microrobots circulating in mouse brain vasculature. Reproduced with permission from ref. [202] Copyright 2022, American Association for the Advancement of Science.

In fluorescence imaging, the fluorescent dyes embedded within MNRs absorb specific excitation wavelengths, subsequently emitting light that is captured and processed by the optical system to generate an image (**Figure** **19**a).^[^ ^145^ ^]^ However, the limited tissue penetration depth and interference from tissue autofluorescence remain significant challenges in the application of MNR‐based fluorescence imaging.^[^ ^135^ ^]^ MRI, with its superior soft‐tissue contrast and deep‐tissue penetration capabilities, has been widely adopted for in vivo tracking of MNRs. Yan et al. integrated ferromagnetic or MNPs into the structure of MNRs to enhance imaging contrast and signal responsiveness, as illustrated in Figure 19b.^[^ ^187^ ^]^ However, MRI is limited by relatively slow imaging speeds and insufficient temporal resolution, making real‐time monitoring of MNRs challenging. Furthermore, interference between the magnetic actuation methods used for MNRs and the MRI imaging modalities used for real‐time navigation can occur. For example, the strong magnetic fields required for propulsion can interfere with MRI image acquisition, while conversely, MRI ultrahigh fields can disrupt robot trajectory.^[^ ^30^, ^195^, ^196^ ^]^

Magnetic particle imaging (MPI), characterized by higher sensitivity and improved spatial resolution, has emerged as a robust complementary technique to MRI.^[^ ^197^ ^]^ Clinically established radiological imaging modalities, such as X‐ray, CT, PET, and fluoroscopy, provide excellent tissue penetration and high spatial resolution. For instance, Jeong et al. successfully demonstrated real‐time navigation and therapeutic application of miniature robots in a porcine model of arterial thromboembolism by using a biplane X‐ray imaging platform.^[^ ^189^ ^]^ However, the lengthy reconstruction times required by CT and PET limit their utility in real‐time intraoperative guidance scenarios, thereby favoring fluoroscopy for clinical applications. Zhang et al. demonstrated the feasibility of real‐time intraoperative tracking using tPA‐functionalized nanorobots delivered precisely via fluoroscopic imaging in a rabbit model (Figure 19c).^[^ ^165^ ^]^ US imaging, characterized by its non‐invasiveness, cost‐effectiveness, and favorable spatiotemporal resolution, has emerged as another compelling modality for MNR tracking. B‐mode ultrasound imaging can effectively illustrate structural echo features of MNRs, whereas Doppler ultrasound provides precise measurements of their motion velocity (Figure 19d).^[^ ^198^ ^]^ However, acoustic shadowing effects resulting from cranial bone structures and physical constraints posed by ultrasound probe operation significantly hinder its applicability in deep intracranial structures.^[^ ^199^ ^]^

In recent years, hybrid imaging techniques such as MRgFUS and PAI have garnered considerable attention.^[^ ^200^, ^201^, ^202^ ^]^ MRgFUS not only facilitates precise image‐guided targeting but also enables the transient opening of the BBB to enhance the efficacy of drug delivery.^[^ ^193^ ^]^ PAI combines the high‐resolution imaging advantages of optical techniques with the deep penetration capabilities afforded by ultrasound, enabling multiscale visualization from micrometer to millimeter‐level resolution. Sitti et al. demonstrated real‐time tracking of individual microrobots as small as 5 µm within mouse cerebral vasculature by using photoacoustic computed tomography (PACT), as shown in Figure 19e.^[^ ^202^ ^]^ Li et al. employed optical‐resolution photoacoustic microscopy to enhance tracking resolution further, highlighting the significant potential of this technology for imaging within the intricate brain microenvironment.^[^ ^203^ ^]^

Imaging and tracking technologies for MNRs are evolving toward multimodal integration, combining high spatiotemporal resolution, deep tissue penetration, and clinical compatibility, to meet the complex requirements of brain disorder treatment. Intraoperative imaging systems such as MRI, CT, fluoroscopy, and fluorescence‐guided techniques enhance navigation accuracy and support precise MNR deployment. Looking ahead, advances in AI and intelligent navigation are expected to enable autonomous operation and multiscale imaging, thereby accelerating the clinical translation of MNRs.

## 6 Conclusion and Future Perspectives

**Figure 20:**
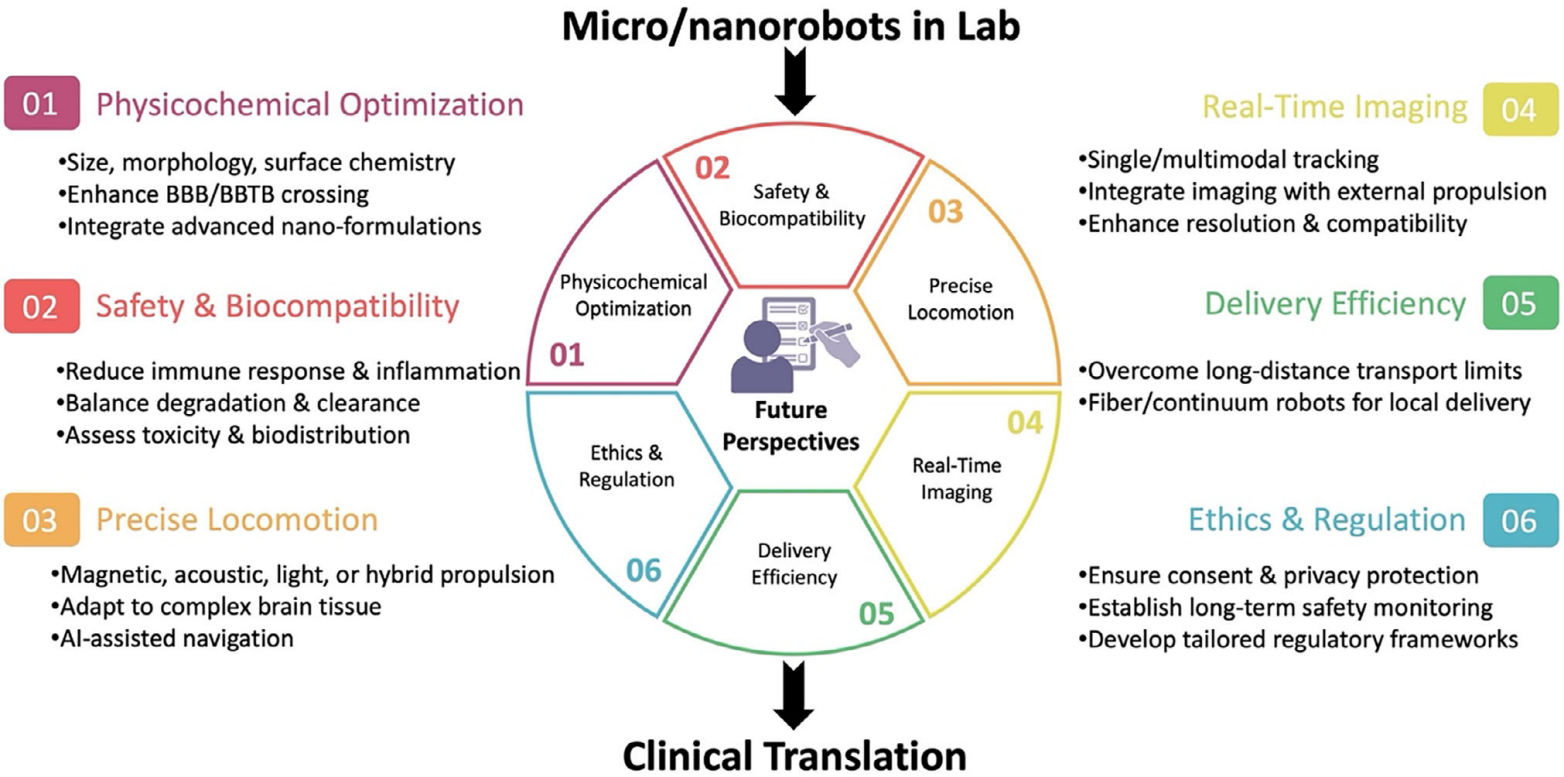
Future perspectives of micro/nanorobots for combating brain disorders.

As a transformative and cutting‐edge technology, MNRs offer unprecedented opportunities for precise treatment and minimally invasive interventions in brain disorders. However, despite significant advancements in recent years, numerous critical challenges, as illustrated in **Figure** **20**, remain before achieving clinical translation and widespread adoption:

### 6.1 Physicochemical Optimization

Optimization of the physicochemical properties of MNRs for treating brain disorders is essential. Parameters such as size, morphology, propulsion mechanisms, surface chemistry, and drug‐functionalization strategies critically influence their ability to traverse the BBB and BBTB, propulsion efficiency, and potential post‐treatment risks. Special emphasis should be placed on strategies to overcome the BBB, such as utilizing stimuli‐responsive coatings or externally triggered transient opening methods (e.g., focused ultrasound), which can significantly improve the efficiency of drug delivery across this critical biological barrier. Advanced nanofabrication technologies should be employed to develop functional nanomaterials with enhanced capabilities for crossing the BBB and BBTB, facilitating the integration of advanced nano‐formulations with MNRs, thereby significantly improving targeted brain delivery efficiency.

### 6.2 Safety and Biocompatibility

Safety and biocompatibility are major challenges limiting the clinical translation of MNRs for brain disorders. The BBB restricts peripheral immune cell entry, but microglia, as resident immune cells, may recognize MNRs and trigger localized inflammation. To mitigate immune clearance, strategies such as polyethylene glycol, biomimetic membrane coatings (e.g., exosome or red blood cell membranes), or autologous blood‐derived materials have been employed, along with optimization of nanoparticle size and morphology or the use of immunosuppressants. Biodegradation kinetics further influence safety. Ideally, MNRs should degrade into small molecules or ions cleared via brain fluid circulation and metabolism. Rapid degradation may reduce efficacy, while slow degradation risks prolonged retention, chronic inflammation, neuronal dysfunction, and protein aggregation (e.g., α‐synuclein), potentially exacerbating neurodegenerative diseases. Residual high‐density or magnetic particles may also disrupt local electric fields and signal transduction. Comprehensive toxicological studies are needed to evaluate degradation products, inflammatory responses, pharmacokinetics, biodistribution, and off‐target accumulation. Interactions between transient BBB‐opening technologies and MNRs also require careful evaluation due to potential immune activation or off‐target risks. Future research should prioritize advanced in vitro brain barrier models and large‐animal studies mimicking human pathophysiology, complemented by real‐time imaging technologies (e.g., MRI) to track MNR biodistribution, degradation, and clearance dynamics, facilitating safer clinical translation.

### 6.3 Precise Locomotion

Precise spatiotemporal control and efficient locomotion of MNRs within complex brain structures remain significant technical challenges. Developing propulsion systems based on magnetic, acoustic, optical, or combined physical fields represents innovative avenues to address these issues. Particularly, magnetic propulsion systems, with their superior remote control capabilities, deep tissue penetration, and excellent controllability, have garnered extensive attention. However, achieving targeted locomotion within the highly heterogeneous and viscoelastic brain parenchyma remains unresolved. Hybrid propulsion approaches, such as combining magnetic navigation with acoustically induced local steering, may improve adaptability to the complex brain environment. Integrating artificial intelligence techniques (such as machine learning and deep learning algorithms) into magnetic navigation systems holds great potential for significantly improving navigation precision and responsiveness in dynamically complex brain environments.

### 6.4 Real‐Time Imaging

Real‐time imaging guidance is critical for achieving precise drug delivery and therapeutic monitoring. Single‐modality and multimodal imaging platforms enable comprehensive tracking of MNR positions, movement trajectories, and dynamic payload release. However, interference between external propulsion systems and imaging platforms urgently requires in‐depth evaluation and resolution. A pressing focus is to integrate propulsion and imaging into a unified system, such as combining MRI with magnetic field actuation, which can simultaneously achieve high‐resolution monitoring and precise navigation of MNRs. Future research should further enhance spatial‐temporal resolution and sensitivity of imaging devices while optimizing compatibility and synergy between propulsion platforms and imaging systems to ensure precision and safety throughout the therapeutic delivery process.

### 6.5 Delivery Efficiency

Significant bottlenecks remain in the long‐distance, highly efficient transport of MNRs within cerebral vasculature and brain parenchyma. Direct systemic administration often results in substantial payload losses, severely limiting therapeutic efficiency. Emerging delivery technologies, such as fiber robots or continuum robots, offer forward‐looking solutions through minimally invasive, catheter‐mediated precise interventions. Fiber robotic technology can precisely deliver MNRs close to targeted cerebral vessels or lesion regions, enabling localized release and subsequent short‐range propulsion, thus significantly reducing transport losses and improving delivery accessibility. However, practical clinical translation requires addressing numerous complex engineering challenges, including the development of advanced control systems, the selection and optimization of biocompatible materials, and the refinement and implementation of real‐time monitoring strategies.

### 6.6 Ethics and Regulation

The clinical translation of MNR technologies for the treatment of brain disorders entails a wide array of ethical, regulatory, and technical challenges that must be carefully addressed. In the context of such advanced but relatively unfamiliar technologies, ensuring informed consent requires clear and transparent communication of risks, benefits, and uncertainties. Long‐term safety monitoring is essential, as the small size of micro‐ and nanomaterials, combined with the application of external control fields (e.g., ultrasound or magnetic fields), may introduce unforeseen risks, including unintended neurological effects or interactions with surrounding tissues. Privacy protection constitutes another critical consideration, particularly when MNR technologies are integrated with data‐driven diagnostic or real‐time monitoring systems. In addition, the societal implications—such as equitable access to treatment and public perception of micro‐ and nanotechnologies—must be carefully evaluated.

Ethical frameworks should therefore be designed to address these issues comprehensively, ensuring that the deployment of MNRs in clinical settings adheres to rigorous safety and ethical standards. Regulatory agencies play a pivotal role in refining approval standards and procedures specific to MNR applications. This includes the development of guidelines for preclinical testing, risk assessment, and post‐market surveillance, to responsibly foster innovation while safeguarding patient safety. Collaboration among researchers, clinicians, regulatory authorities, and ethicists is indispensable for addressing the complexities associated with the deployment of microrobotics and for promoting their safe and effective integration into clinical practice.

Looking forward, establishing MNRs as reliable clinical tools necessitates close interdisciplinary collaboration. Clinicians must clearly define therapeutic needs, clinical scenarios, and feasibility of treatment protocols. Engineers and materials scientists should continuously innovate propulsion technologies and develop scalable, efficient manufacturing processes and biocompatible materials. Biologists need to thoroughly assess interactions between MNRs and biological environments, evaluating therapeutic effectiveness and safety. Imaging specialists should focus on optimizing real‐time imaging technologies for precise monitoring of MNRs. Systematic comparative studies between micro‐ and nanorobotic therapies and conventional treatments are critical to clarify the clinical advantages and cost‐effectiveness of this emerging therapeutic platform. Specifically, future research may explore the dual‐function strategy of acoustic fields to both temporarily open the BBB and drive MNRs through localized acoustic streaming. Similarly, MRI‐based systems hold promise for next‐generation diagnostics and therapeutics by simultaneously utilizing magnetic field gradients to drive MNRs and monitoring their distribution through high‐resolution imaging. These potential research directions could greatly accelerate the clinical feasibility of MNRs in brain medicine.

In conclusion, although numerous challenges still exist in MNR application to treat brain disorders, this promising platform possesses substantial scientific value and development potential. Through persistent interdisciplinary collaboration, technological innovation, rigorous safety and efficacy evaluation, and meticulous ethical and regulatory management, MNR technology is poised to profoundly reshape clinical treatment paradigms for neurological diseases, brain tumors, and various other brain disorders in the future.

## Conflict of Interest

The authors declare no conflict of interest.

## References

[1] J. Olesen , M. Leonardi , Eur. J. Neurol. 2003, 10, 471. 12940825 10.1046/j.1468-1331.2003.00682.x

[2] K. Aho , P. Harmsen , S. Hatano , J. Marquardsen , V. E. Smirnov , T. Strasser , Bull W. H O. 1980, 58, 113. 6966542 PMC2395897

[3] M. Portegies , P. Koudstaal , M. Ikram , Handbook Clin. Neurol. 2016, 138, 239. 10.1016/B978-0-12-802973-2.00014-8 27637962

[4] C. W. Tsao , A. W. Aday , Z. I. Almarzooq , C. A. Anderson , P. Arora , C. L. Avery , C. M. Baker‐Smith , A. Z. Beaton , A. K. Boehme , A. E. Buxton , Y. Commodore‐Mensah , M. S. Elkind , K. R. Evenson , C. Eze‐Nliam , S. Fugar , G. Generoso , D. G. Heard , S. Hiremath , J. E. Ho , R. Kalani , D. S. Kazi , D. Ko , D. A. Levine , J. Liu , J. Ma , J. W. Magnani , E. D. Michos , M. E. Mussolino , S. D. Navaneethan , N. I. Parikh , Circulation 2023, 147, 93. 10.1161/CIR.0000000000001123 PMC12135016 36695182

[5] W. J. Powers , A. A. Rabinstein , T. Ackerson , O. M. Adeoye , N. C. Bambakidis , K. Becker , J. Biller , M. Brown , B. M. Demaerschalk , B. Hoh , E. C. Jauch , C. S. Kidwell , T. M. Leslie‐Mazwi , B. Ovbiagele , P. A. Scott , K. N. Sheth , A. M. Southerland , D. V. Summers , D. L. Tirschwell , Stroke 2019, 50, 344. 31662037 10.1161/STR.0000000000000211

[6] F. Herpich , F. Rincon , Crit. Care Med. 2020, 48, 1654. 32947473 10.1097/CCM.0000000000004597 PMC7540624

[7] C.‐W. Hung , Y.‐C. Chen , W.‐L. Hsieh , S.‐H. Chiou , C.‐L. Kao , Ageing Res. Rev. 2010, 9, S36. 20732460 10.1016/j.arr.2010.08.006

[8] E. L. Feldman , S. A. Goutman , S. Petri , L. Mazzini , M. G. Savelieff , P. J. Shaw , G. Sobue , Lancet 2022, 400, 1363. 36116464 10.1016/S0140-6736(22)01272-7 PMC10089700

[9] J. S. Graves , K. M. Krysko , L. H. Hua , M. Absinta , R. J. M. Franklin , B. M. Segal , Lancet Neurol. 2023, 22, 66. 36216015 10.1016/S1474-4422(22)00184-3

[10] T. Foltynie , V. Bruno , S. Fox , A. A. Kühn , F. Lindop , A. J. Lees , Lancet 2024, 403, 305. 38245250 10.1016/S0140-6736(23)01429-0

[11] M. Jung , H. Kim , J. W. Hwang , Y. Choi , M. Kang , C. Kim , J. Hong , N. K. Lee , S. Moon , J. W. Chang , S.‐J. Choi , S.‐Y. Oh , H. Jang , D. L. Na , B.‐S. Kim , Nano Lett. 2023, 23, 476. 36638236 10.1021/acs.nanolett.2c03682

[12] T. B. Stoker , S. L. Mason , J. C. Greenland , S. T. Holden , H. Santini , R. A. Barker , Practical Neurol. 2022, 22, 32. 10.1136/practneurol-2021-003074 34413240

[13] M. Goldsmith , L. Abramovitz , D. Peer , ACS Nano 2014, 8, 1958. 24660817 10.1021/nn501292z

[14] R. N. L. Lamptey , B. Chaulagain , R. Trivedi , A. Gothwal , B. Layek , J. Singh , Int. J. Mol. Sci. 2022, 23, 1851. 35163773 10.3390/ijms23031851 PMC8837071

[15] G. Livingston , J. Huntley , A. Sommerlad , D. Ames , C. Ballard , S. Banerjee , C. Brayne , A. Burns , J. Cohen‐Mansfield , C. Cooper , S. G. Costafreda , A. Dias , N. Fox , L. N. Gitlin , R. Howard , H. C. Kales , M. Kivimäki , E. B. Larson , A. Ogunniyi , V. Orgeta , K. Ritchie , K. Rockwood , E. L. Sampson , Q. Samus , L. S. Schneider , G. Selbæk , L. Teri , N. Mukadam , Lancet 2020, 396, 413. 32738937 10.1016/S0140-6736(20)30367-6 PMC7392084

[16] W. H. Organization , Global status report on the public health response to dementia 2021.

[17] Y. Ale , N. Nainwal , Mol. Pharmaceutics 2023, 20, 4893. 10.1021/acs.molpharmaceut.3c00554 37647568

[18] H. Sung , J. Ferlay , R. L. Siegel , M. Laversanne , I. Soerjomataram , A. Jemal , F. Bray , Ca‐Cancer J. Clin. 2021, 71, 209. 33538338 10.3322/caac.21660

[19] K. D. Miller , Q. T. Ostrom , C. Kruchko , N. Patil , T. Tihan , G. Cioffi , H. E. Fuchs , K. A. Waite , A. Jemal , R. L. Siegel , J. S. Barnholtz‐Sloan , Ca‐Cancer J. Clin. 2021, 71, 381. 34427324 10.3322/caac.21693

[20] A. C. Tan , D. M. Ashley , G. Y. López , M. Malinzak , H. S. Friedman , M. Khasraw , Ca‐Cancer J. Clin. 2020, 70, 299. 32478924 10.3322/caac.21613

[21] M. Mao , Y. Wu , Q. He , Nanoscale 2024, 16, 8689. 38606460 10.1039/d4nr01056f

[22] A. S. Achrol , R. C. Rennert , C. Anders , R. Soffietti , M. S. Ahluwalia , L. Nayak , S. Peters , N. D. Arvold , G. R. Harsh , P. S. Steeg , S. D. Chang , Nat. Rev. Dis. Primers 2019, 5, 5. 30655533 10.1038/s41572-018-0055-y

[23] R. J. Bystritsky , F. C. Chow , Neurolog. Clin. 2022, 40, 77. 10.1016/j.ncl.2021.08.006 34798976

[24] B. Garber , J. Glauser , Curr. Emergency Hospital Med. Rep. 2024, 12, 95. 10.1007/s40138-020-00218-1 PMC7296288 32837804

[25] GBD 2016 Traumatic Brain Injury and Spinal Cord Injury Collaborators , Lancet Neurol. 2019, 18, 56.30497965

[26] A. A. Hyder , C. A. Wunderlich , P. Puvanachandra , G. Gururaj , O. C. Kobusingye , NeuroRehabilitation 2007, 22, 341. 18162698

[27] J. H. Krystal , Cell 2014, 157, 201.24679536

[28] S. Zha , H. Liu , H. Li , H. Li , K.‐L. Wong , A. H. All , ACS Nano 2024, 18, 1820. 38193927 10.1021/acsnano.3c10674 PMC10811692

[29] K. Mnich , S. Lhomond , E. Wallace , P.‐J. Le Reste , A. Pandit , E. Chevet , C. Reidy , A. Samali , G. Duffy , A. M. Gorman , Device 2025, 3, 100685.

[30] M. Tian , Z. Ma , G.‐Z. Yang , The Innovation 2024, 5, 100548. 38161522 10.1016/j.xinn.2023.100548 PMC10757293

[31] B. Engelhardt , P. Vajkoczy , R. O. Weller , Nat. Immunol. 2017, 18, 123. 28092374 10.1038/ni.3666

[32] D. Furtado , M. Björnmalm , S. Ayton , A. I. Bush , K. Kempe , F. Caruso , Adv. Mater. 2018, 30, 1801362. 10.1002/adma.201801362 30066406

[33] S. H. Lim , G. T. Yee , D. Khang , Int. J. Nanomed. 2024, 2529. 10.2147/IJN.S450853 PMC10949308 38505170

[34] P. Sharma , A. Aaroe , J. Liang , V. K. Puduvalli , Neuro‐Oncol. Adv. 2023, 5, vdad009. 10.1093/noajnl/vdad009 PMC10034917 36968288

[35] Y. Liu , H. Wu , G. Liang , Biomater. Res. 2025, 29, 0133. 39911305 10.34133/bmr.0133 PMC11794768

[36] H. Chen , T. Li , Z. Liu , S. Tang , J. Tong , Y. Tao , Z. Zhao , N. Li , C. Mao , J. Shen , M. Wan , Nat. Commun. 2023, 14, 941. 36804924 10.1038/s41467-022-35709-0 PMC9941476

[37] X. Dong , Theranostics 2018, 8, 1481. 29556336 10.7150/thno.21254 PMC5858162

[38] D. Wu , Q. Chen , X. Chen , F. Han , Z. Chen , Y. Wang , Signal Transduct. Target. Ther. 2023, 8, 217. 37231000 10.1038/s41392-023-01481-w PMC10212980

[39] Y. Liu , K. M. C. Bravo , J. Liu , Nanoscale Horiz. 2021, 6, 78. 33400747 10.1039/d0nh00605j

[40] D. Hwang , J. D. Ramsey , A. V. Kabanov , Adv. Drug Delivery Rev. 2020, 156, 80. 10.1016/j.addr.2020.09.009 PMC8173698 32980449

[41] M. J. Mitchell , M. M. Billingsley , R. M. Haley , M. E. Wechsler , N. A. Peppas , R. Langer , Nat. Rev. Drug Discovery 2021, 20, 101. 33277608 10.1038/s41573-020-0090-8 PMC7717100

[42] F. Wu , J. Liu , Adv. Drug Delivery Rev. 2022, 188, 114443. 10.1016/j.addr.2022.114443 35817214

[43] V. Sharma , C. D. Mukhopadhyay , Extracellular Vesicle 2024, 3, 100032.

[44] S. Dutta , S. Noh , R. S. Gual , X. Chen , S. Pané , B. J. Nelson , H. Choi , Nano‐Micro Lett. 2023, 16, 41. 10.1007/s40820-023-01259-3 PMC10689718 38032424

[45] T. Qiu , T.‐C. Lee , A. G. Mark , K. I. Morozov , R. Münster , O. Mierka , S. Turek , A. M. Leshansky , P. Fischer , Nat. Commun. 2014, 5, 5119, 10.1038/ncomms6119. 25369018 PMC4241991

[46] C. Chen , S. Ding , J. Wang , Nat. Rev. Mater. 2024, 9, 159, 10.1038/s41578-023-00641-2.

[47] E. Lauga , Soft Matter 2011, 7, 3060.

[48] M. Safdar , S. U. Khan , J. Jänis , Adv. Mater. 2018, 30, 1703660. 10.1002/adma.201703660 29411445

[49] S. J. Ebbens , J. R. Howse , Langmuir 2011, 27, 12293. 21928845 10.1021/la2033127

[50] L. Soler , V. Magdanz , V. M. Fomin , S. Sanchez , O. G. Schmidt , ACS Nano 2013, 7, 9611. 24180623 10.1021/nn405075d PMC3872448

[51] R. Liu , A. Sen , J. Am. Chem. Soc. 2011, 133, 20064. 21961523 10.1021/ja2082735

[52] J. G. Gibbs , Y.‐P. Zhao , Appl. Phys. Lett. 2009, 94, 163104.

[53] A. A. Solovev , Y. Mei , E. Bermúdez Ureña , G. Huang , O. G. Schmidt , Small 2009, 5, 1688. 19373828 10.1002/smll.200900021

[54] M. E. Ibele , Y. Wang , T. R. Kline , T. E. Mallouk , A. Sen , J. Am. Chem. Soc. 2007, 129, 7762. 17550256 10.1021/ja0726512

[55] W. F. Paxton , A. Sen , T. E. Mallouk , Chem.– A Eur. J. 2005, 11, 6462. 10.1002/chem.200500167 16052651

[56] S. Sánchez , L. Soler , J. Katuri , Angew. Chem., Int. Ed. 2015, 54, 1414. 10.1002/anie.201406096 25504117

[57] H. Li , Z. Sun , S. Jiang , X. Lai , A. Böckler , H. Huang , F. Peng , L. Liu , Y. Chen , Nano Lett. 2019, 19, 8749. 31671944 10.1021/acs.nanolett.9b03456

[58] H. Yuan , X. Liu , L. Wang , X. Ma , Bioact. Mater. 2021, 6, 1727. 33313451 10.1016/j.bioactmat.2020.11.022 PMC7711193

[59] J. Wu , S. Ma , M. Li , X. Hu , N. Jiao , S. Tung , L. Liu , ACS Appl. Mater. Interfaces 2021, 13, 31514. 34213305 10.1021/acsami.1c07593

[60] S. Cao , H. Wu , I. A. B. Pijpers , J. Shao , L. K. E. A. Abdelmohsen , D. S. Williams , J. C. M. van Hest , ACS Nano 2021, 15, 18270. 34668368 10.1021/acsnano.1c07343 PMC8613902

[61] S. Tang , F. Zhang , H. Gong , F. Wei , J. Zhuang , E. Karshalev , B. Esteban‐Fernández de Ávila , C. Huang , Z. Zhou , Z. Li , L. Yin , H. Dong , R. H. Fang , X. Zhang , L. Zhang , J. Wang , Sci. Rob. 2020, 5, aba6137. 10.1126/scirobotics.aba6137 33022613

[62] X. Arqué , A. Romero‐Rivera , F. Feixas , T. Patiño , S. Osuna , S. Sánchez , Nat. Commun. 2019, 10, 2826. 31249381 10.1038/s41467-019-10726-8 PMC6597730

[63] Y. Wu , Z. Song , G. Deng , K. Jiang , H. Wang , X. Zhang , H. Han , Small 2021, 17, 2006877. 10.1002/smll.202006877 33619851

[64] M. Zhou , T. Hou , J. Li , S. Yu , Z. Xu , M. Yin , J. Wang , X. Wang , ACS Nano 2019, 13, 1324. 30689352 10.1021/acsnano.8b06773

[65] W. Gao , R. Dong , S. Thamphiwatana , J. Li , W. Gao , L. Zhang , J. Wang , ACS Nano 2015, 9, 117. 25549040 10.1021/nn507097k PMC4310033

[66] J. Ou , H. Tian , J. Wu , J. Gao , J. Jiang , K. Liu , S. Wang , F. Wang , F. Tong , Y. Ye , L. Liu , B. Chen , X. Ma , X. Chen , F. Peng , Y. Tu , ACS Appl. Mater. Interfaces 2021, 13, 38050. 34369138 10.1021/acsami.1c08926

[67] W. Wang , C. Zhou , Adv. Healthcare Mater. 2021, 10, 2001236.

[68] C. K. Schmidt , M. Medina‐Sánchez , R. J. Edmondson , O. G. Schmidt , Nat. Commun. 2020, 11, 5618. 33154372 10.1038/s41467-020-19322-7 PMC7645678

[69] B. Fu , D. Luo , C. Li , Y. Feng , W. Liang , Front. Chem. 2025, 13, 1537917. 39981265 10.3389/fchem.2025.1537917 PMC11839623

[70] J. Wang , Y. Dong , P. Ma , Y. Wang , F. Zhang , B. Cai , P. Chen , B.‐F. Liu , Adv. Mater. 2022, 34, 2201051. 10.1002/adma.202201051 35385160

[71] V. Garcia‐Gradilla , S. Sattayasamitsathit , F. Soto , F. Kuralay , C. Yardimci , D. Wiitala , M. Galarnyk , J. Wang , Small 2014, 10, 4154. 24995778 10.1002/smll.201401013

[72] B. Esteban‐Fernández de Ávila , C. Angell , F. Soto , M. A. Lopez‐Ramirez , D. F. Báez , S. Xie , J. Wang , Y. Chen , ACS Nano 2016, 10, 4997. 27022755 10.1021/acsnano.6b01415

[73] B. Esteban‐Fernández de Ávila , D. E. Ramírez‐Herrera , S. Campuzano , P. Angsantikul , L. Zhang , J. Wang , ACS Nano 2017, 11, 5367. 28467853 10.1021/acsnano.7b01926 PMC5894870

[74] T. Xu , L.‐P. Xu , X. Zhang , Appl. Mater. Today 2017, 9, 493.

[75] W. Wang , L. A. Castro , M. Hoyos , T. E. Mallouk , ACS Nano 2012, 6, 6122. 22631222 10.1021/nn301312z

[76] C. Dillinger , J. Knipper , N. Nama , D. Ahmed , Nanoscale 2024, 16, 1125. 37946510 10.1039/d3nr03516f PMC10795801

[77] T. Xu , F. Soto , W. Gao , R. Dong , V. Garcia‐Gradilla , E. Magaña , X. Zhang , J. Wang , J. Am. Chem. Soc. 2015, 137, 2163. 25634724 10.1021/ja511012v

[78] D. Ahmed , T. Baasch , B. Jang , S. Pane , J. Dual , B. J. Nelson , Nano Lett. 2016, 16, 4968. 27459382 10.1021/acs.nanolett.6b01601

[79] M. Kaynak , A. Ozcelik , A. Nourhani , P. E. Lammert , V. H. Crespi , T. J. Huang , Lab Chip 2017, 17, 395. 27991641 10.1039/c6lc01272h PMC5465869

[80] D. Kagan , M. J. Benchimol , J. C. Claussen , E. Chuluun‐Erdene , S. Esener , J. Wang , Angew. Chem., Int. Ed. 2012, 51, 7519. 10.1002/anie.201201902 PMC3477603 22692791

[81] A. Aghakhani , O. Yasa , P. Wrede , M. Sitti , Proc. Natl. Acad. Sci. USA 2020, 117, 3469. 32015114 10.1073/pnas.1920099117 PMC7035478

[82] T. Xu , F. Soto , W. Gao , V. Garcia‐Gradilla , J. Li , X. Zhang , J. Wang , J. Am. Chem. Soc. 2014, 136, 8552. 24898345 10.1021/ja504150e

[83] Y. Hou , H. Wang , R. Fu , X. Wang , J. Yu , S. Zhang , Q. Huang , Y.u Sun , T. Fukuda , Lab Chip 2023, 23, 848. 36629004 10.1039/d2lc00573e

[84] A. I. Bunea , D. Martella , S. Nocentini , C. Parmeggiani , R. Taboryski , D. S. Wiersma , Adv. Intell. Syst. 2021, 3, 2000256.

[85] A. Neettiyath , M. Pumera , Adv. Funct. Mater. 2025, 35, 2415875.

[86] S. Zhang , B. Xu , M. Elsayed , F. Nan , W. Liang , J. K. Valley , L. Liu , Q. Huang , M. C. Wu , A. R. Wheeler , Chem. Soc. Rev. 2022, 51, 9203. 36285556 10.1039/d2cs00359g

[87] J. R. Moffitt , Y. R. Chemla , S. B. Smith , C. Bustamante , Annu. Rev. Biochem. 2008, 77, 205. 18307407 10.1146/annurev.biochem.77.043007.090225

[88] S. Zhang , E. Y. Scott , J. Singh , Y. Chen , Y. Zhang , M. Elsayed , M. D. Chamberlain , N. Shakiba , K. Adams , S. Yu , C. M. Morshead , P. W. Zandstra , A. R. Wheeler , Proc. Natl. Acad. Sci. USA 2019, 116, 14823. 31289234 10.1073/pnas.1903406116 PMC6660717

[89] G. T. Iványi , B. Nemes , I. Gróf , T. Fekete , J. Kubacková , Z. Tomori , G. Bánó , G. Vizsnyiczai , L. Kelemen , Adv. Mater. 2024, 36, 2401115. 10.1002/adma.202401115 38814436

[90] U. G. Būtaitė , G. M. Gibson , Y.‐L. D. Ho , M. Taverne , J. M. Taylor , D. B. Phillips , Nat. Commun. 2019, 10, 1215. 30872572 10.1038/s41467-019-08968-7 PMC6418258

[91] J. Qin , X. Wu , A. Krueger , B. Hecht , Nat. Commun. 2025, 16, 2570. 40089456 10.1038/s41467-025-57871-x PMC11910605

[92] Y. Gao , X. Wang , Y. Chen , RSC Adv. 2024, 14, 14278. 38694551 10.1039/d4ra00495g PMC11062240

[93] Q. Cheng , X. Lu , Y. Tai , T. Luo , R. Yang , ACS Biomater. Sci. Eng. 2024, 10, 5562. 39147594 10.1021/acsbiomaterials.4c01191

[94] M. Zhang , A. Pal , Z. Zheng , G. Gardi , E. Yildiz , M. Sitti , Nat. Mater. 2023, 22, 1243. 37604911 10.1038/s41563-023-01649-3 PMC10533409

[95] C. Ni , D. Chen , X. Wen , B. Jin , Y.i He , T. Xie , Q. Zhao , Nat. Commun. 2023, 14, 7672. 37996451 10.1038/s41467-023-43576-6 PMC10667353

[96] Z. Zhan , L. Chen , H. Duan , Y. Chen , M. He , Z. Wang , Int. J. Extreme Manufactur. 2022, 4, 015302.

[97] J. Liu , L. Jiang , S. He , J. Zhang , W. Shao , Chem. Eng. J. 2022, 433, 133496.

[98] H. Guo , T.‐P. Ruoko , H. Zeng , A. Priimagi , Adv. Funct. Mater. 2024, 34, 2312068.

[99] H. Zhang , A. Mourran , M. Möller , Nano Lett. 2017, 17, 2010. 28181437 10.1021/acs.nanolett.7b00015 PMC6291182

[100] H. Zhang , L. Koens , E. Lauga , A. Mourran , M. Möller , Small 2019, 15, 1903379. 10.1002/smll.201903379 31553139

[101] C. Xin , Z. Ren , L. Zhang , L. Yang , D. Wang , Y. Hu , J. Li , J. Chu , L.i Zhang , D. Wu , Nat. Commun. 2023, 14, 4273. 37460571 10.1038/s41467-023-40038-x PMC10352372

[102] M. Sitti , D. S. Wiersma , Adv. Mater. 2020, 32, 1906766. 10.1002/adma.201906766 32053227

[103] M.‐S. Kim , H.‐T. Lee , S.‐H. Ahn , Adv. Mater. Technol. 2019, 4, 1900583.

[104] H. Xin , N. Zhao , Y. Wang , X. Zhao , T. Pan , Y. Shi , B. Li , Nano Lett. 2020, 20, 7177. 32935992 10.1021/acs.nanolett.0c02501

[105] S. Du , H. Wang , C. Zhou , W. Wang , Z. Zhang , J. Am. Chem. Soc. 2020, 142, 2213. 31957432 10.1021/jacs.9b13093

[106] W. Li , W. Wang , X. Dong , Y. Sun , ACS Appl. Mater. Interfaces 2020, 12, 12618. 32105446 10.1021/acsami.0c02342

[107] S. Palagi , D. P. Singh , P. Fischer , Adv. Opt. Mater. 2019, 7, 1900370.

[108] B. Wang , K. Kostarelos , B. J. Nelson , L. Zhang , Adv. Mater. 2021, 33, 2002047. 10.1002/adma.202002047 33617105

[109] T. Yamanaka , F. Arai , IEEE Robot. Automat. Lett. 2024, 9, 747.

[110] Z. Liang , H. Joh , B. Lian , D. E. Fan , Sci. Adv. 2023, 9, adi9932. 10.1126/sciadv.adi9932 PMC10599615 37878697

[111] F. Katzmeier , F. C. Simmel , Nat. Commun. 2023, 14, 6247. 37802992 10.1038/s41467-023-41923-1 PMC10558450

[112] T. Michálek , A. Bolopion , Z. Hurák , M. Gauthier , IEEE/ASME Trans. Mechatron. 2020, 25, 828.

[113] X. Liang , F. Mou , Z. Huang , J. Zhang , M. You , L. Xu , M. Luo , J. Guan , Adv. Funct. Mater. 2020, 30, 1908602.

[114] L. Zhang , Z. Xiao , X. Chen , J. Chen , W. Wang , ACS Nano 2019, 13, 8842. 31265246 10.1021/acsnano.9b02100

[115] T. Yamanaka , F. Arai , IEEE Robot. Automat. Lett. 2018, 3, 1787.

[116] J. Guo , J. J. Gallegos , A. R. Tom , D. Fan , ACS Nano 2018, 12, 1179. 29303550 10.1021/acsnano.7b06824

[117] G. Loget , A. Kuhn , Nat. Commun. 2011, 2, 535. 22086336 10.1038/ncomms1550

[118] Y. Dong , L. Wang , J. Wang , S. Wang , Y. Wang , D. Jin , P. Chen , W. Du , L.i Zhang , B.‐F. Liu , ACS Nano 2020, 14, 16600. 33119265 10.1021/acsnano.0c07067

[119] Z. Wu , Y. Chen , D. Mukasa , O. S. Pak , W. Gao , Chem. Soc. Rev. 2020, 49, 8088. 32596700 10.1039/d0cs00309c

[120] L. Zhang , J. J. Abbott , L. Dong , B. E. Kratochvil , D. Bell , B. J. Nelson , Appl. Phys. Lett. 2009, 94, 064107.

[121] H. Xie , M. Sun , X. Fan , Z. Lin , W. Chen , L. Wang , L. Dong , Q. He , Sci. Rob. 2019, 4, aav8006. 10.1126/scirobotics.aav8006 33137748

[122] K. Villa , L. Krejčová , F. Novotný , Z. Heger , Z. Sofer , M. Pumera , Adv. Funct. Mater. 2018, 28, 1804343.

[123] A. Ghosh , P. Fischer , Nano Lett. 2009, 9, 2243. 19413293 10.1021/nl900186w

[124] S. Tottori , L. Zhang , F. Qiu , K. K. Krawczyk , A. Franco‐Obregón , B. J. Nelson , Adv. Mater. 2012, 24, 811. 22213276 10.1002/adma.201103818

[125] H. Zhou , C. C. Mayorga‐Martinez , S. Pané , L. Zhang , M. Pumera , Chem. Rev. 2021, 121, 4999. 33787235 10.1021/acs.chemrev.0c01234 PMC8154323

[126] X. Wu , J. Liu , C. Huang , M. Su , T. Xu , IEEE Trans. Automat. Sci. Eng. 2019, 17, 823.

[127] Y. Hou , H. Wang , S. Zhong , Y. Qiu , Q. Shi , T. Sun , Q. Huang , T. Fukuda , IEEE/ASME Trans. Mechatron. 2022, 28, 429.

[128] T. Li , J. Li , H. Zhang , X. Chang , W. Song , Y. Hu , G. Shao , E. Sandraz , G. Zhang , L. Li , J. Wang , Small 2016, 12, 6098. 27600373 10.1002/smll.201601846

[129] P. Liao , L. Xing , S. Zhang , D. Sun , Small 2019, 15, 1901197. 10.1002/smll.201901197 31314164

[130] S. Kim , S. Lee , J. Lee , B. J. Nelson , L. Zhang , H. Choi , Sci. Rep. 2016, 6, 30713, 10.1038/srep30713. 27470077 PMC4965827

[131] T. Li , J. Li , K. I. Morozov , Z. Wu , T. Xu , I. Rozen , A. M. Leshansky , L. Li , J. Wang , Nano Lett. 2017, 17, 5092. 28677387 10.1021/acs.nanolett.7b02383

[132] Y. Jia , P. Liao , Y. Wang , D. Sun , Adv. Intell. Syst. 2022, 4, 2200118.

[133] T. Li , A. Zhang , G. Shao , M. Wei , B. Guo , G. Zhang , L. Li , W. Wang , Adv. Funct. Mater. 2018, 28, 1706066.

[134] E. Diller , M. Sitti , Adv. Funct. Mater. 2014, 24, 4397.

[135] Q.i Zhang , Y. Qu , H. Zhao , S. Chen , Z. Liu , J. Li , Y. Li , J. Li , D. Sun , ACS Appl. Mater. Interfaces 2024, 16, 50344. 39265074 10.1021/acsami.4c10301

[136] Y. Li , D. Dong , Y. Qu , J. Li , S. Chen , H. Zhao , Q.i Zhang , Y. Jiao , L. Fan , D. Sun , Small 2023, 19, 2301889. 10.1002/smll.202301889 37423966

[137] J. Li , X. Li , T. Luo , R. Wang , C. Liu , S. Chen , D. Li , J. Yue , S.‐H. Cheng , D. Sun , Sci. Rob. 2018, 3, aat8829. 10.1126/scirobotics.aat8829 33141689

[138] S. Chen , Z. Tan , P. Liao , Y. Li , Y. Qu , Q.i Zhang , M. Yang , K. W. Y. Chan , L.i Zhang , K. Man , Z. Chen , D. Sun , Adv. Healthcare Mater. 2023, 12, 2202921. 10.1002/adhm.202202921 37156574

[139] T. Wei , J. Li , L. Zheng , F. Li , H. Tian , D. Sun , Adv. Intell. Syst. 2021, 3, 2100052.

[140] J. J. Abbott , O. Ergeneman , M. P. Kummer , A. M. Hirt , B. J. Nelson , IEEE Trans. Robot. 2007, 23, 1247.

[141] N. Murali , S. B. Das , S. Yadav , S. K. Rainu , N. Singh , S. Betal , Adv. Mater. Technol. 2024, 9, 2400239.

[142] Y. Alapan , O. Yasa , B. Yigit , I. C. Yasa , P. Erkoc , M. Sitti , Ann. Rev. Control, Robot. Autono. Syst. 2019, 2, 205.

[143] L. Xie , Z. Cong , S. Tang , M. Yang , Y. Li , C. Ren , Q. Chen , D. Lu , F. Wan , X. Zhang , S. Wu , Mater. Today Chem. 2023, 30, 101560.

[144] Z. Cong , S. Tang , L. Xie , M. Yang , Y. Li , D. Lu , J. Li , Q. Yang , Q. Chen , Z. Zhang , X. Zhang , S. Wu , Adv. Mater. 2022, 34, 2201042. 10.1002/adma.202201042 35452560

[145] H. Zhang , Z. Li , C. Gao , X. Fan , Y. Pang , T. Li , Z. Wu , H. Xie , Q. He , Sci. Rob. 2021, 6, aaz9519. 10.1126/scirobotics.aaz9519 34043546

[146] F. Zhang , R. Mundaca‐Uribe , H. Gong , B. Esteban‐Fernández de Ávila , M. Beltrán‐Gastélum , E. Karshalev , A. Nourhani , Y. Tong , B. Nguyen , M. Gallot , Y. Zhang , L. Zhang , J. Wang , Adv. Mater. 2019, 31, 1901828. 10.1002/adma.201901828 31070278

[147] Y. Alapan , O. Yasa , O. Schauer , J. Giltinan , A. F. Tabak , V. Sourjik , M. Sitti , Sci. Rob. 2018, 3, aar4423. 10.1126/scirobotics.aar4423 33141741

[148] O. Felfoul , M. Mohammadi , S. Taherkhani , D. de Lanauze , Y. Zhong Xu , D. Loghin , S. Essa , S. Jancik , D. Houle , M. Lafleur , L. Gaboury , M. Tabrizian , N. Kaou , M. Atkin , T.é Vuong , G. Batist , N. Beauchemin , D. Radzioch , S. Martel , Nat. Nanotechnol. 2016, 11, 941. 27525475 10.1038/nnano.2016.137 PMC6094936

[149] C. C. Mayorga‐Martinez , J. Zelenka , J. Grmela , H. Michalkova , T. Ruml , J. Mares , M. Pumera , Adv. Sci. 2021, 8, 2101301. 10.1002/advs.202101301 PMC8498868 34369099

[150] Q. Chen , S. Tang , Y. Li , Z. Cong , D. Lu , Q. Yang , X. Zhang , S. Wu , ACS Appl. Mater. Interfaces 2021, 13, 58382. 34860489 10.1021/acsami.1c18597

[151] V. Magdanz , I. S. M. Khalil , J. Simmchen , G. P. Furtado , S. Mohanty , J. Gebauer , H. Xu , A. Klingner , A. Aziz , M. Medina‐Sánchez , O. G. Schmidt , S. Misra , Sci. Adv. 2020, 6, aba5855. 10.1126/sciadv.aba5855 PMC7450605 32923590

[152] Z. Li , Z. Guo , F. Zhang , L. Sun , H. Luan , Z. Fang , J. L. Dedrick , Y. Zhang , C. Tang , A. Zhu , Y. Yu , S. Ding , D. Wang , A.n‐Y.i Chang , L.u Yin , L. M. Russell , W. Gao , R. H. Fang , L. Zhang , J. Wang , Nat. Commun. 2025, 16, 666. 39809831 10.1038/s41467-025-56032-4 PMC11733022

[153] F. Zhang , Z. Li , C. Chen , H. Luan , R. H. Fang , L. Zhang , J. Wang , Adv. Mater. 2024, 36, 2303714. 10.1002/adma.202303714 PMC10799182 37471001

[154] F. Zhang , Z. Guo , Z. Li , H. Luan , Y. Yu , A. T. Zhu , S. Ding , W. Gao , R. H. Fang , L. Zhang , J. Wang , Sci. Adv. 2024, 10, adn6157. 10.1126/sciadv.adn6157 PMC11168470 38865468

[155] Z. Li , Y. Duan , F. Zhang , H. Luan , W.‐T. Shen , Y. Yu , N. Xian , Z. Guo , E. Zhang , L. Yin , R. H. Fang , W. Gao , L. Zhang , J. Wang , Sci. Rob. 2024, 9, adl2007. 10.1126/scirobotics.adl2007 38924422

[156] M. B. Akolpoglu , N. O. Dogan , U. Bozuyuk , H. Ceylan , S. Kizilel , M. Sitti , Adv. Sci. 2020, 7, 2001256. 10.1002/advs.202001256 PMC7435244 32832367

[157] M. B. Akolpoglu , S. F. Baltaci , U. Bozuyuk , S. Karaz , M. Sitti , Matter 2025, 8, 102052.

[158] D. Gong , N. Celi , D. Zhang , J. Cai , ACS Appl. Mater. Interfaces 2022, 14, 6320. 35020358 10.1021/acsami.1c16859

[159] B. Li , J. Yang , S. Lu , J. Zhao , Y. Du , Y. Cai , R. Dong , Nano Lett. 2024, 25, 48. 39680918 10.1021/acs.nanolett.4c03870

[160] N. Celi , J. Cai , H. Sun , L. Feng , D. Zhang , D. Gong , ACS Appl. Mater. Interfaces 2024, 16, 24341. 38687629 10.1021/acsami.4c02836

[161] C. Yan , K. Feng , B. Bao , J. Chen , X. Xu , G. Jiang , Y. Wang , J. Guo , T. Jiang , Y.u Kang , C. Wang , C. Li , C. Zhang , P. Nie , S. Liu , H.‐G. Machens , L. Zhu , X. Yang , R. Niu , Z. Chen , Adv. Sci. 2024, 11, 2404456. 10.1002/advs.202404456 PMC11336935 38894569

[162] A. Zarepour , A. Khosravi , S. Iravani , A. Zarrabi , Adv. Healthcare Mater. 2024, 13, 2402102. 10.1002/adhm.202402102 PMC11650542 39373299

[163] J. Zheng , R. Qi , C. Dai , G. Li , M. Sang , ACS Nano 2022, 16, 2330. 35138084 10.1021/acsnano.1c08538

[164] H. Bao , S. Zhang , J. Luo , J. Meng , S. Wang , Angew. Chem. 2025, 137, 202503221. 10.1002/anie.202503221 40304594

[165] B. Wang , Q. Wang , K. F. Chan , Z. Ning , Q. Wang , F. Ji , H. Yang , S. Jiang , Z. Zhang , B. Y. M. Ip , H. Ko , J. P. W. Chung , M. Qiu , J. Han , P. W. Y. Chiu , J. J. Y. Sung , S. Du , T. W. H. Leung , S. C. H. Yu , L. Zhang , Sci. Adv. 2024, 10, adk8970. 10.1126/sciadv.adk8970 PMC10830105 38295172

[166] M. Yang , Y. Zhang , F. Mou , C. Cao , L. Yu , Z. Li , J. Guan , Sci. Adv. 2023, 9, adk7251. 10.1126/sciadv.adk7251 PMC10686566 38019908

[167] J. Wu , W. Zou , Q. Lu , T. Zheng , Y. Li , T. Ying , Y. Li , Y. Zheng , L. Wang , Adv. Sci. 2025, 12, 2410351. 10.1002/advs.202410351 PMC11831500 39731361

[168] L. Wang , J. Wang , J. Hao , Z. Dong , J. Wu , G. Shen , T. Ying , L. Feng , X. Cai , Z. Liu , Y. Zheng , Adv. Mater. 2021, 33, 2105351. 10.1002/adma.202105351 34647345

[169] D. Jin , Q. Wang , K. F. Chan , N. Xia , H. Yang , Q. Wang , S. C. H. Yu , L. Zhang , Sci. Adv. 2023, 9, adf9278. 10.1126/sciadv.adf9278 PMC10181194 37172097

[170] J. Liu , S. Wang , S. Huang , K.e Zhang , Yulu li , Z. Chen , C. Huang , Y. Zhang , S. Du , T. Xu , Cell Rep. Phys. Sci. 2024, 5, 102160.

[171] J. Wang , Q. Zhou , Q.i Dong , J. Shen , J. Hao , D. Li , T. Xu , X. Cai , W. Bai , T. Ying , Y. Li , L. Zhang , Y. Zhu , L. Wang , J. Wu , Y. Zheng , Small 2024, 20, 2400408. 10.1002/smll.202400408 38709208

[172] X. Liu , L. Wang , Y. Xiang , F. Liao , N.a Li , J. Li , J. Wang , Q. Wu , C. Zhou , Y. Yang , Y. Kou , Y. Yang , H. Tang , N. Zhou , C. Wan , Z. Yin , G.‐Z. Yang , G. Tao , J. Zang , Sci. Rob. 2024, 9, adh2479. 10.1126/scirobotics.adh2479 38381840

[173] B. Wang , J. Shen , C. Huang , Z. Ye , J. He , X. Wu , Z. Guo , L.i Zhang , T. Xu , Nat. Biomed. Eng. 2025, 9, 1471. 40312457 10.1038/s41551-025-01382-z

[174] M. Li , J. Wu , N. Li , J. Zhou , W. Cheng , A. Wu , L. Liu , N. Jiao , Adv. Funct. Mater. 2024, 34, 2402333.

[175] J. Wu , N. Jiao , D. Lin , N. Li , T. Ma , S. Tung , W. Cheng , A. Wu , L. Liu , Adv. Mater. 2024, 36, 2306876. 10.1002/adma.202306876 37899660

[176] T. Kang , G. D. Cha , O. K. Park , H. R. Cho , M. Kim , J. Lee , D. Kim , B. Lee , J. Chu , S. Koo , T. Hyeon , D.‐H. Kim , S. H. Choi , ACS Nano 2023, 17, 5435. 36926815 10.1021/acsnano.2c10094

[177] X. Song , A. Gul , H. Zhao , R. Qian , L. Fang , C. Huang , L. Xi , L. Wang , U. K. Cheang , ACS Appl. Mater. Interfaces 2025, 17, 5784. 39818731 10.1021/acsami.4c16960

[178] B. Li , X. Chen , W. Qiu , R. Zhao , J. Duan , S. Zhang , Z. Pan , S. Zhao , Q. Guo , Y. Qi , W. Wang , L. Deng , S. Ni , Y. Sang , H. Xue , H. Liu , G. Li , Adv. Sci. 2022, 9, 2105451. 10.1002/advs.202105451 PMC9189685 35508804

[179] Z. Wang , C. Wang , Y. Ji , M. Yang , C. Li , M. Li , J. Yang , H. Tang , X. Luo , H. Hao , Z. Liu , K. Chen , Y. Chang , H. Yuan , L. Feng , G. Xing , J. Li , The Innovation 2025, 6, 100777. 39991478 10.1016/j.xinn.2024.100777 PMC11846086

[180] P. Mayorga‐Burrezo , C. C. Mayorga‐Martinez , M. Pumera , Adv. Funct. Mater. 2022, 32, 2106699.

[181] Y. Gao , Y. Guo , Y. Yang , Y. Tang , B. Wang , Q. Yan , X. Chen , J. Cai , L. Fang , Z. Xiong , F. Gao , C. Wu , J. Wang , J. Tang , L. Shi , D. Li , Adv. Mater. 2024, 36, 2305632. 10.1002/adma.202305632 37805826

[182] S. Cesur , M. E. Cam , F. S. Sayin , O. Gunduz , J. Drug Deliv. Sci. Technol. 2022, 67, 102977.

[183] J. Ye , Y. Fan , Y. She , J. Shi , Y. Yang , X. Yuan , R. Li , J. Han , L. Liu , Y. Kang , X. Ji , Adv. Sci. 2024, 11, 2310211. 10.1002/advs.202310211 PMC11165487 38460166

[184] H. Wang , X. Chen , Y. Qi , C. Wang , L. Huang , R. Wang , J. Li , X. Xu , Y. Zhou , Y. Liu , X. Xue , Adv. Mater. 2022, 34, 2206779. 10.1002/adma.202206779 36189876

[185] M. Zhang , L. Yang , H. Yang , L. Su , J. Xue , Q. Wang , B. Hao , Y. Jiang , K. F. Chan , J. J. Y. Sung , H.o Ko , X. Liu , L. Wang , B. Y. M. Ip , T. W. H. Leung , L.i Zhang , Sci. Adv. 2025, 11, adv1682. 10.1126/sciadv.adv1682 PMC13108821 40540555

[186] X. Wang , Z. Gong , T. Wang , J. Law , X. Chen , S. Wanggou , J. Wang , B. Ying , M. Francisco , W. Dong , Y. Xiong , J. J. Fan , G. MacLeod , S. Angers , X. Li , P. B. Dirks , X. Liu , X. Huang , Y. Sun , Sci. Adv. 2023, 9, ade5321. 10.1126/sciadv.ade5321 PMC10058241 36989359

[187] X. Yan , Q.i Zhou , M. Vincent , Y. Deng , J. Yu , J. Xu , T. Xu , T. Tang , L. Bian , Y.‐X. J. Wang , K. Kostarelos , L.i Zhang , Sci. Rob. 2017, 2, aaq1155. 10.1126/scirobotics.aaq1155 33157904

[188] D. Vilela , U. Cossío , J. Parmar , A. M. Martínez‐Villacorta , V. Gómez‐Vallejo , J. Llop , S. Sánchez , ACS Nano 2018, 12, 1220. 29361216 10.1021/acsnano.7b07220

[189] S. Jeong , H. Choi , G. Go , C. Lee , K. S. Lim , D. S. Sim , M. H. Jeong , S. Y. Ko , J.‐O. Park , S. Park , Med. Eng. Phys. 2016, 38, 403. 26857290 10.1016/j.medengphy.2016.01.001

[190] E. Ren , J. Hu , Z. Mei , L. Lin , Q. Zhang , P. He , J. Wang , T. Sheng , H. Chen , H. Cheng , T. Xu , S. Pang , Y. Zhang , Q. Dai , X. Gao , H. Liu , H. Li , Y. Zhao , Z. Gu , X. Yan , G. Liu , Adv. Mater. 2025, 37, 2412187. 10.1002/adma.202412187 39538994

[191] J. Yu , D. Jin , K.‐F. Chan , Q. Wang , K. Yuan , L. Zhang , Nat. Commun. 2019, 10, 5631. 31822669 10.1038/s41467-019-13576-6 PMC6904566

[192] L. Xie , X. Pang , X. Yan , Q. Dai , H. Lin , J. Ye , Y. Cheng , Q. Zhao , X. Ma , X. Zhang , G. Liu , X. Chen , ACS Nano 2020, 14, 2880. 32125820 10.1021/acsnano.9b06731

[193] S. Alli , C. A. Figueiredo , B. Golbourn , N. Sabha , M. Y. Wu , A. Bondoc , A. Luck , D. Coluccia , C. Maslink , C. Smith , H. Wurdak , K. Hynynen , M. O'Reilly , J. T. Rutka , J. Controlled Release 2018, 281, 29. 10.1016/j.jconrel.2018.05.005 PMC6026028 29753957

[194] D. W. Kim , P. Wrede , A. Rodríguez‐Camargo , Y. Chen , N. O. Dogan , C. Glück , B. V. Lotsch , D. Razansky , M. Sitti , Adv. Mater. 2025, 2418425. 10.1002/adma.202418425 PMC12691905 40052638

[195] N. Li , P. Fei , C. Tous , M. Rezaei Adariani , M.‐L. Hautot , I. Ouedraogo , A. Hadjadj , I. P. Dimov , Q. Zhang , S. Lessard , Z. Nosrati , C. N. Ng , K. Saatchi , U. O. Häfeli , C. Tremblay , S. Kadoury , A. Tang , S. Martel , G. Soulez , Sci. Rob. 2024, 9, adh8702. 10.1126/scirobotics.adh8702 38354257

[196] M. E. Tiryaki , Y. G. Elmacıoğlu , M. Sitti , Sci. Adv. 2023, 9, adg6438. 10.1126/sciadv.adg6438 PMC10132757 37126547

[197] F. Griese , T. Knopp , C. Gruettner , F. Thieben , K. Müller , S. Loges , P. Ludewig , N. Gdaniec , J. Magn. Magn. Mater. 2020, 498, 166206.

[198] Q. Wang , K. F. Chan , K. Schweizer , X. Du , D. Jin , S. C. H. Yu , B. J. Nelson , L. Zhang , Sci. Adv. 2021, 7, abe5914. 10.1126/sciadv.abe5914 PMC7909881 33637532

[199] Q. Wang , F. Zhao , B. Wang , K. F. Chan , B. Y. M. Ip , T. W. H. Leung , X. Song , L. Zhang , J. Xie , The Innovation 2025, 6, 100874. 40528888 10.1016/j.xinn.2025.100874 PMC12169274

[200] C. Gasca‐Salas , B. Fernández‐Rodríguez , J. A. Pineda‐Pardo , R. Rodríguez‐Rojas , I. Obeso , F. Hernández‐Fernández , M. del Álamo , D. Mata , P. Guida , C. Ordás‐Bandera , J. I. Montero‐Roblas , R. Martínez‐Fernández , G. Foffani , I. Rachmilevitch , J. A. Obeso , Nat. Commun. 2021, 12, 779. 33536430 10.1038/s41467-021-21022-9 PMC7859400

[201] A. Abrahao , Y. Meng , M. Llinas , Y. Huang , C. Hamani , T. Mainprize , I. Aubert , C. Heyn , S. E. Black , K. Hynynen , N. Lipsman , L. Zinman , Nat. Commun. 2019, 10, 4373. 31558719 10.1038/s41467-019-12426-9 PMC6763482

[202] P. Wrede , O. Degtyaruk , S. K. Kalva , X. L. Deán‐Ben , U. Bozuyuk , A. Aghakhani , B. Akolpoglu , M. Sitti , D. Razansky , Sci. Adv. 2022, 8, abm9132. 10.1126/sciadv.abm9132 PMC9094653 35544570

[203] D. Li , C. Liu , Y. Yang , L. Wang , Y. Shen , Light: Sci. Appl. 2020, 9, 84. 32411369 10.1038/s41377-020-0323-y PMC7214411

